# Fibroblast growth factor receptor inhibition for succinate dehydrogenase-deficient gastrointestinal stromal tumors: a phase 2 trial

**DOI:** 10.1038/s41591-026-04376-9

**Published:** 2026-05-26

**Authors:** Priscilla Merriam, James J. Morrow, Emanuele Mazzola, Nicole L. Solimini, Prafulla C. Gokhale, Ping Chi, Alice P. Chen, Mark Agulnik, Melissa Burgess, Scott M. Schuetze, Neeta Somaiah, Brian A. Van Tine, Seth M. Pollack, Gabriel Tinoco, Jonathan Trent, Breelyn A. Wilky, Nicola Bothwick, Benjamin K. Eschle, Vivian Nguyen, Jan H. Beumer, Noushin Rastkari, Shahanawaz Jiwani, Peter I-Fan Wu, Lorraine Pelosof, Matthew L. Hemming, Geoffrey I. Shapiro, George Demetri, Bradley Bernstein, Suzanne George

**Affiliations:** 1 Dana-Farber Cancer Institute, Boston, MA USA; 2 Department of Pediatric Oncology, Dana-Farber Cancer Institute, Boston, MA USA; 3 Division of Hematology/Oncology, Boston Children’s Hospital, Boston, MA USA; 4 Harvard Medical School, Boston, MA USA; 5 Ludwig Center at Harvard Medical School, Boston, MA USA; 6 Memorial Sloan Kettering Cancer Center, New York City, NY USA; 7 Weill Cornell Medicine, New York, NY USA; 8 Division of Cancer Treatment and Diagnosis, National Cancer Institute, Bethesda, MD USA; 9 City of Hope Comprehensive Cancer Center, Duarte, CA USA; 10 UPMC Hillman Cancer Center, Pittsburgh, PA USA; 11 University of Michigan Comprehensive Cancer Center, Ann Arbor, MI USA; 12 The University of Texas MD Anderson Cancer Center, Houston, TX USA; 13 Washington University School of Medicine, St. Louis, MO USA; 14 Northwestern Memorial Hospital, Chicago, IL USA; 15 The Ohio State University Comprehensive Cancer Center, Columbus, OH USA; 16 Sylvester Comprehensive Cancer Center, University of Miami, Miami, FL USA; 17 University of Colorado School of Medicine, Aurora, CO USA; 18 Department of Oncology, Johns Hopkins University School of Medicine and Johns Hopkins Sidney Kimmel Cancer Center, Baltimore, MD USA; 19 Molecular Characterization Laboratory, Frederick National Laboratory for Cancer Research, Frederick, MD USA; 20 UMass Chan Medical School, Worcester, MA USA

**Keywords:** Sarcoma, Cancer epigenetics, Targeted therapies

## Abstract

While most gastrointestinal stromal tumors are driven by oncogenic mutations in *KIT* or *PDGFRA*, 10–15% exhibit functional loss of the succinate dehydrogenase (SDH) complex and genome-wide DNA hypermethylation. Excess methylation in SDH-deficient gastrointestinal stromal tumors disrupts genomic insulators, inducing aberrant expression of oncogenic ligands *FGF3*, *FGF4*, and activating an autocrine signaling loop mediated through FGFR1. We conducted a phase 2 trial of pan-fibroblast growth factor receptor inhibitor rogaratinib in patients with sarcoma and report here on the cohort of patients with advanced SDH-deficient GIST. The primary objective was to estimate objective response rate. Secondary objectives were to estimate progression-free survival (PFS) and assess safety and tolerability. Exploratory objectives were to evaluate serial measurements of *FGF3* and *FGF4* and fibroblast growth factor receptors in serial biopsies, to perform whole-exome sequencing in serial biopsies and to explore rogaratinib exposure with pharmacodynamic effects. Twenty-four patients received rogaratinib and ten experienced partial responses for an objective response rate of 41.7%. Median PFS was 31.0 months (95% confidence interval 20.2–not reached), and 1-year PFS was 77.4% (95% confidence interval 61.7–97.1). Toxicities were manageable and included hyperphosphatemia, fatigue and diarrhea. Elevations in phosphorous were seen across the cohort, consistent with target engagement of FGFR1. Whole-exome and next-generation sequencing revealed alterations in the SDH subunit coding genes (*SDHx*) as expected. This trial illustrates a successful demonstration of targeted cancer therapy predicated on an epigenetic mechanism of oncogene activation. Clinicaltrials.gov identifier: NCT04595747.

## Abstract (web summary)

In a multicenter phase 2 trial, the fibroblast growth factor receptor inhibitor rogaratinib showed encouraging clinical efficacy in patients with succinate dehydrogenase-deficient gastrointestinal stromal tumors, suggesting a potential new treatment option for this patient population and demonstrating that an epigenetic mechanism of oncogene activation can be successfully targeted with a tyrosine kinase inhibitor.

## Main

Targeted cancer therapy has been underpinned by the identification of genetic alterations that drive oncogenesis. However, epigenetic alterations, including aberrant DNA methylation, are widespread and increasingly recognized for their potential to silence tumor suppressors and activate oncogenes^1^. Despite extensive preclinical research, the degree to which epigenetic alterations can direct effective targeted treatments in the clinic remains an open question.

Gastrointestinal stromal tumors (GISTs) are the most common sarcoma arising in the gastrointestinal tract^2^. Although the majority are driven by gain-of-function mutations in *KIT* or *PDGFRA*, about 10–15% of adult and most pediatric GISTs lack genetic alteration of these or other canonical oncogenes. Rather, these tumors are characterized by functional loss of the mitochondrial SDH complex due to mutations in an SDH subunit gene or hypermethylation of the *SDHC* promoter without underlying subunit mutation^3,4^. Because functional loss of any individual subunit results in disruption of the entire complex, SDH-deficient (SDHd) GISTs are typically diagnosed on the basis of a lack of SDHB protein expression by immunohistochemistry (IHC) and an absence of canonical oncogene mutations^3^. In contrast to other GIST subtypes, historical approaches to the management of advanced and metastatic tumors have resulted in limited objective radiographic responses^4–10^ (Extended Data Table 1).

SDHd tumors exhibit profound DNA hypermethylation because DNA demethylases are inhibited by the excess succinate associated with SDH loss^11^. DNA hypermethylation has been described in a wide range of tumor types, where it has been associated with tumor suppressor gene silencing, suppression of interferon signaling and deregulation of proto-oncogenes^12–19^. In SDHd GIST, aberrant methylation disrupts a ‘boundary element’ that insulates the *FGF3* and *FGF4* genes. This aberrant methylation event reorganizes the locus and causes a nearby enhancer to drive expression of these oncogenic ligands, which drive autocrine signaling in GIST cells that already express multiple fibroblast growth factor receptors (FGFRs), including FGFR1^15^. FGFR inhibitors (FGFRi) have shown clinical efficacy in other tumors harboring FGFR fusions or genetic alterations that drive aberrant signaling, including cholangiocarcinoma (pemigatinib, futibatinib, infigratinib and erdafitinib) and urothelial cancer (erdafitinib)^20–25^.

Given preclinical evidence that FGFR signaling represents a defining epigenetically driven oncogenic program in SDHd GIST, we evaluated the efficacy of an FGFR1–4 inhibitor, rogaratinib (BAY 1163877), in people with advanced SDHd GIST. In addition, we present in vivo evaluations of multiple FGFRi that support a potential class effect of this approach.

## Results

We conducted a multicenter phase 2 trial of pan-FGFRi rogaratinib in patients with sarcoma and report here on the cohort with advanced SDHd GIST. Eligible patients were 18 years of age or older with an Eastern Cooperative Oncology Group Performance Status (ECOG) of 2 or better, and measurable disease by Response Evaluation Criteria in Solid Tumors 1.1 (RECIST). The study team reviewed pathology reports that documented the diagnosis of SDHd GIST for each patient for approval before registration. The primary objective was to estimate the objective response rate. Secondary objectives were to estimate PFS and assess safety and tolerability. Exploratory objectives were to evaluate serial measurements of *FGF3* and *FGF4* and FGFR in serial biopsies, to perform whole-exome sequencing in serial biopsies, and to explore rogaratinib exposure with pharmacodynamic effects.

### Patients

**Fig. 1:**
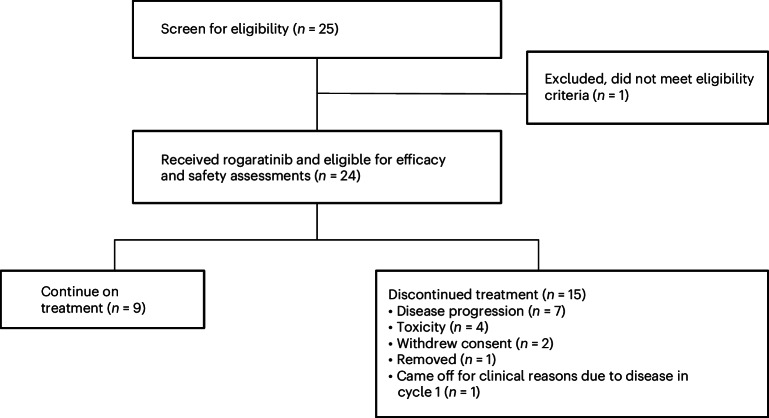
CONSORT diagram. Patient disposition throughout the study.

From 4 May 2021 to 21 August 2023, 24 eligible adult patients were enrolled from 11 centers in the USA (Fig. 1). Demographic data^26^ are summarized in Table 1. Median age was 52 (range: 22–88) years, and 54% of patients were women. The baseline ECOG for 79% of patients was 0, and the remaining patients had an ECOG of 1. All tumors showed an absence of SDHB per IHC, with 50% having documented *SDHA* subunit mutations or loss of SDHA protein expression by IHC, which predicts *SDHA* mutation^27^. Most patients were previously treated (67%), and tyrosine kinase inhibitor was the most common type of prior systemic therapy (58%). Eight patients (33%) had received prior treatment with a multi-targeted tyrosine kinase inhibitor, including sunitinib and regorafenib.

**Table 1:** Demographics

Characteristic	
Age (median, range)	52 (22–88)
Sex	
Female	13 (54%)
Male	11 (46%)
ECOG	
0	19 (79%)
1	5 (21%)
2	0 (0%)
SDH complex subunit alteration	
*SDHA* mutation^a^	12 (50%)
*SDHB* mutation^b^	5 (20.8%)
*SDHC* mutation	2 (8%)
*SDHD* mutation	0
Likely SDHC hypermethylation^c^	3 (12.5%)
Unknown	2 (8%)
Primary site	
Gastric	21 (87.5%)
Other^d^	3 (12.5%)
Prior gastrectomy	
Total	2 (8%)
Partial	17 (71%)
None	4 (17%)
Unknown	1 (4%)
Number of prior systemic therapies (median, range)	1 (0–5)
No prior systemic therapy	8 (33%)
Patients receiving at least one prior systemic therapy	16 (67%)
Patients receiving prior tyrosine kinase inhibitors	14 (58%)
Imatinib	11 (45%)
Sunitinib	8 (33%)
Regorafenib	2 (8%)
Other tyrosine kinase inhibitor	2 (8%)
Prior DNA alkylating agent	3 (12.5%)
Prior investigational agent	4 (17%)
Immunotherapy	1 (4%)

^a^One patient had loss of SDHA expression by IHC, which predicts *SDHA* mutation; Next-generation sequencing (NGS) unavailable.

^b^One patient had *SDHB* copy number loss without documented SDHB mutation.

^c^Lack of *SDHx* mutation on NGS but loss of SDHB expression by IHC.

^d^Other primary sites reported as small intestine (1), epigastrium (1), gastrointestinal tract (1).

### Efficacy

**Fig. 2:**
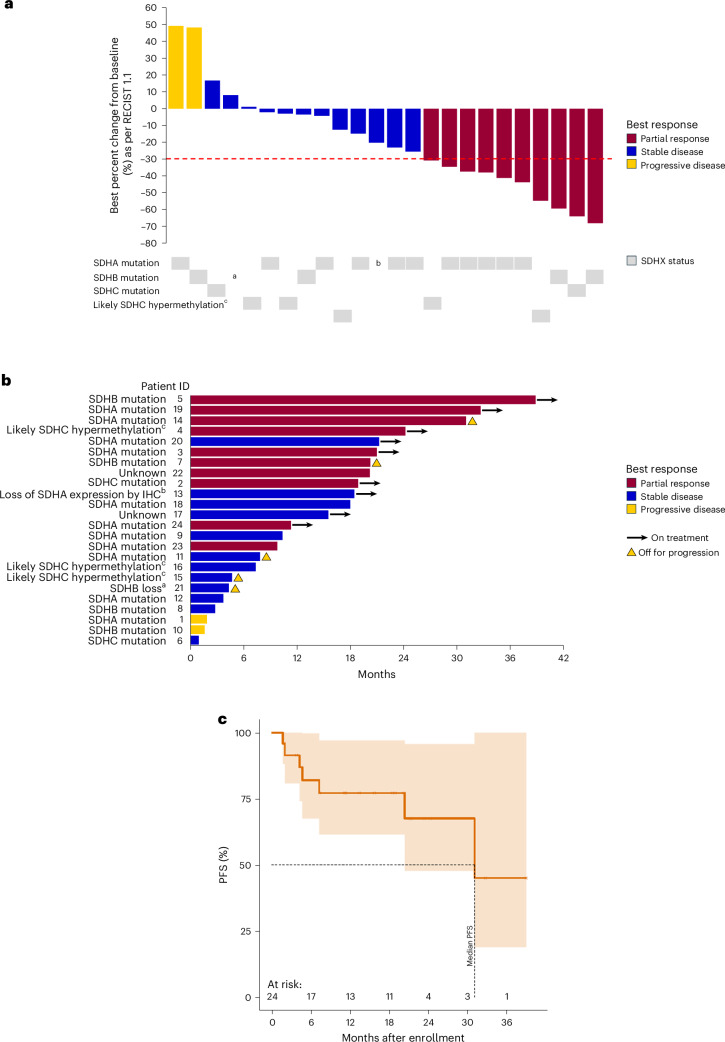
Tumor response. Efficacy of rogaratinib in SDHd GIST (*n* = 24). **a**, Waterfall plot demonstrating best response per patient treated annotated with SDH status. PR, 10 of 24 (41.7%); SD, 12 of 24 (50.0%); PS, 2 of 24 (8.3%). **b**, Swimmer plot demonstrating duration of treatment with rogaratinib per patient annotated with SDH status. **c**, Kaplan–Meier analysis of PFS for the entire cohort. The shaded area in **c** show the 95% CI interval. ^a^SDHB copy number loss. ^b^Loss of SDHA expression by IHC, which predicts SDHA mutation; NGS unavailable. ^c^Lack of SDH mutation on NGS but loss of SDHB expression by IHC. Source data

As of 1 August 2024, 10 of the 24 patients who received rogaratinib (41.7%) had a best objective response of partial response (PR) per RECIST 1.1, with at least 30% reduction in tumor RECIST measurements (range: −31% to −68%) (Fig. 2a). Twelve patients had stable disease (SD) as best response (range: +17% to −25%), and two had progressive disease (PD). PFS at 1 year was 77.4% (95% confidence interval (CI) 61.7–97.1), and median PFS was 31.0 months (95% CI 20.2–not reached) (Fig. 2c). Twelve of 24 patients (50%) were continuing on treatment at 1 year, 4 patients for at least 2 years, and 1 patient was on treatment for more than 3 years (Fig. 2b). Nine patients remained on treatment as of the data cut. Objective responses were seen in all SDH molecular subsets (Fig. 2a,b).

### Safety

Toxicities seen with rogaratinib were generally manageable and typical of those seen with other FGFRi^28^ (Extended Data Table 2). All patients experienced at least one adverse event (AE), with most toxicities being mild to moderate; there were no grade 4 or 5 AEs. The most frequently observed AEs were hyperphosphatemia (96%), alopecia (58%), fatigue (54%), nausea (54%) and diarrhea (50%). Grade 3 toxicities that occurred in two or more participants included hypertension, diarrhea and tumor hemorrhage. Dose reductions were common (63%) but did not definitively appear to impact efficacy (Extended Data Tables 3 and 4). For the 15 patients requiring dose reduction, the most common reasons were hyperphosphatemia (47%), nail changes (20%) and palmar–plantar erythrodysesthesia syndrome (20%).

The most common reason for treatment discontinuation was disease progression (*n* = 7). Other reasons for treatment discontinuation were due to toxicity (*n* = 4): one for grade 2 hyperphosphatemia, one for grade 3 leg pain, one for grade 2 erythema nodosum and one for worsening dry eye in a patient with underlying Sjogren’s syndrome. Two patients withdrew consent, unrelated to toxicity; one patient was removed from the study for nonadherence to the protocol; and one patient was removed for clinical reasons due to disease complications while in cycle 1.

### Exploratory endpoints

#### Pharmacokinetics

**Fig. 3:**
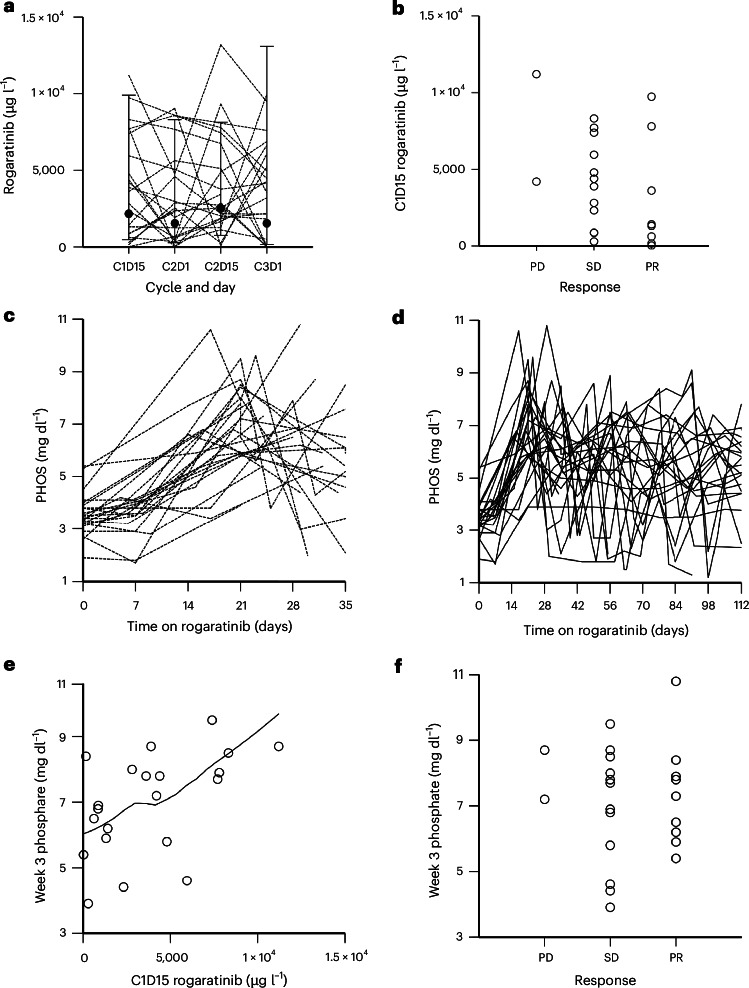
Rogaratinib pharmacokinetic exposure response assessment. **a**, Pre-dose concentrations of rogaratinib; individual patients (dashed lines) and geometric mean and s.d. (solid circles and error bars). C1D15, *n* = 22; C2D1, *n* = 22; C2D15, *n* = 21; C3D1, *n* = 21. **b**, Cycle 1 day 15 rogaratinib exposure by response group (Jonckheere Terpstra test; *P* = 0.119). **c**,**d**, Serum phosphate dynamics during the first 5 weeks (**c**) and first 4 months (**d**) of treatment. **e**, Serum phosphate versus cycle 1 day 15 pre-dose rogaratinib concentration (with LOESS regression; *P* = 0.0038 by linear regression). **f**, Week 3 serum phosphate concentrations by response group (Jonckheere Terpstra test; *P* = 0.656). All tests were two-sided and no CIs are applicable. PHOS, phosphate. Source data

To assess whether treatment response or toxicity was associated with rogaratinib exposure, we characterized the pharmacokinetics of the patients in the study. Data were available for 23 of 24 patients. Although drug exposure varied between patients, this did not correlate with treatment response (Fig. 3a,b). Hyperphosphatemia is a well-described on-target effect of FGFR1 inhibition^28^. It was the most common AE and the most common reason for dose reduction in our study. We therefore examined the relationship between rogaratinib exposure and serum phosphate levels. All patients in the study had increased serum phosphate levels over the first 28 days of treatment (Fig. 3c), but levels largely stabilized with ongoing treatment after 28 days (Fig. 3d). Serum phosphate levels were directly correlated with rogaratinib exposure (Fig. 3e), but also did not correlate with treatment response (Fig. 3f). These results are consistent with a direct, on-target effect of FGFR1 inhibition and effectively provide a pharmacodynamic proxy for rogaratinib in our study. The absence of a correlation between rogaratinib exposure and treatment response suggests that responses were likely caused by underlying differences in tumor biology rather than variation in drug exposure.

### RNA sequencing and whole-exome sequencing

**Fig. 4:**
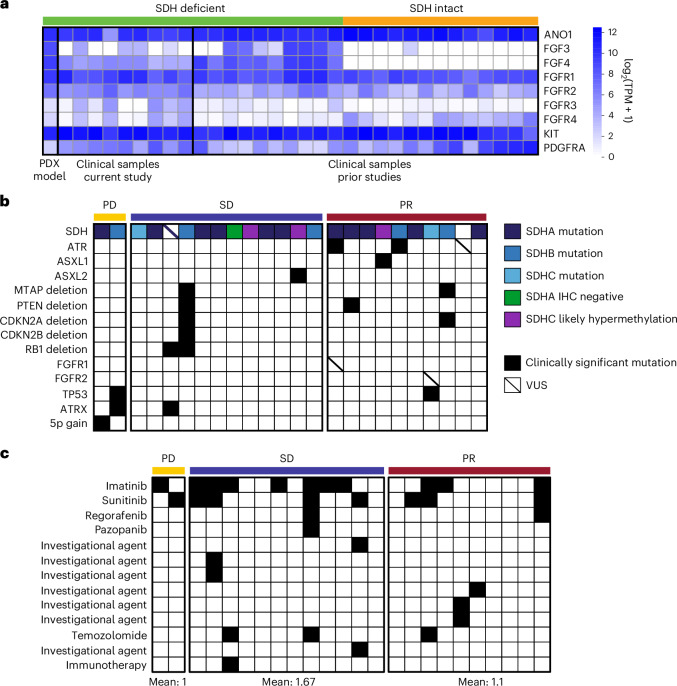
Molecular characterization of tumors and prior treatments of patients on study^26^. **a**, Heatmap of quantile normalized log_2_(transcripts per million) values of relevant genes in SDHd tumor specimens from this trial cohort compared to a previously published PDX model^15^ (left) and SDHd^15^ or intact GIST specimens from prior studies (right)^29^. **b**, Summary of mutations detected by whole-exome sequencing and/or clinical sequencing in tumor specimens stratified by response group. **c**, Summary of treatments before trial enrollment of patients stratified by response group. The mean number of prior treatments for each group is listed below. VUS, variant of uncertain significance.

To investigate determinants of response and potential markers of intrinsic treatment resistance, we performed RNA sequencing (RNA-seq) and whole-exome sequencing on nine pre-treatment tumor specimens and reviewed available clinical sequencing data from archival material (Fig. 4a,b). Serial measurements of FGFR and FGFR ligand, and post-progression whole-exome sequencing were not possible because of a lack of post-progression biopsies; however, we compared expression data for this cohort to RNA-seq profiles acquired previously for other SDHd, KIT- and PDGFRA-mutant GIST cohorts^29^. The GIST markers ANO1, KIT and PDGFRA were expressed across all tumors in these cohorts. *FGFR1* and *FGFR2* showed relatively consistent expression across the different GIST subtypes, while *FGFR3* and *FGFR4* were more variable. In contrast to these receptors, the fibroblast growth factor (FGF) ligands were expressed almost exclusively in the SDHd cohorts. In particular, *FGF4* was highly expressed in all tumors in the trial cohort and in all SDHd GISTs from the prior dataset. *FGF3* expression was more variable but also largely specific to the SDHd cohorts. These expression data are consistent with prior studies and provide support for an autocrine signaling loop in SDHd GIST cells mediated by concurrent expression of FGF ligands and FGFRs^15,30^.

**Extended Data Fig. 1:**
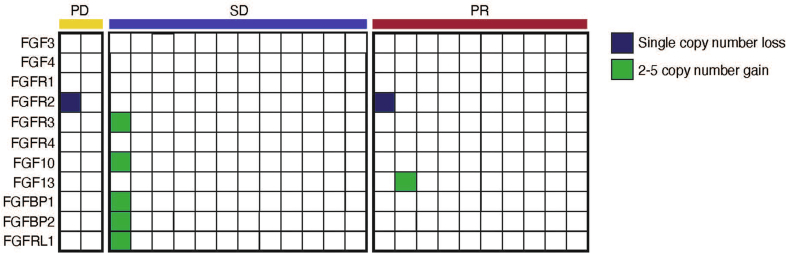
FGF Pathway Mutations in Patient Tumors. Summary of mutations detected by whole exome sequencing in tumor specimens stratified by response group. All identified point mutations and copy number alterations in FGF pathway genes are included.

We next examined the exome sequencing data for the nine tumor specimens, as well as any available clinical sequencing data (Fig. 4b). SDH subunit alterations could be confirmed or predicted in a majority of the tumors (12 *SDHA*, 5 *SDHB*, 2 *SDHC*). All but two of the confirmed SDH subunit alterations were SDH subunit mutations. One tumor had loss of SDHA expression by IHC, which predicts *SDHA* mutation^27^, and one tumor had *SDHB* copy number loss (SDHB sub-gene deep deletion of exons 6–8 with estimated copy number 0 by StrataNGS testing, unclear whether germline or somatic) without documented *SDHB* point mutation. Three of the tumors without identifiable SDH subunit alterations fit a profile of *SDHC* promoter hypermethylation. For two tumors, no genomic SDHX lesion of clinical relevance could be identified, despite loss of SDHB expression per IHC. One of these tumors had an *SDHA* G134V mutation, which is a variant of unknown clinical relevance. We could not identify any clear association between SDH alteration and response to rogaratinib, nor did we identify any other mutations outside of SDH that were predictive of primary response or primary resistance. Of note, we did not identify any copy number gains or point mutations in *FGFR1*, *FGFR2*, *FGF3* or *FGF4* (Extended Data Fig. 1). We did observe that one of the primary progressors had 5p gain, a genetic mutation associated with aggressive GIST disease biology^31,32^, while the second had co-occurring mutations in *TP53* and *ATRX*, which have been generally associated with aggressive tumor phenotypes^33–35^. Both mutation patterns were unique to patients with PD, but caution is warranted given the small number of primary progressors.

In addition, we looked at number of prior treatments across response groups. When compared to patients with PRs, those with SD had generally more prior treatments (mean prior treatments 1.67 versus 1.1) (Fig. 4c).

These results confirm that the SDHd GISTs in our trial cohort harbor expected biomarkers and expression patterns consistent with autocrine FGF signaling and nominate potential markers of aggressive tumor biology for future study.

### Potential class effect for FGFRi in SDHd GIST

**Fig. 5:**
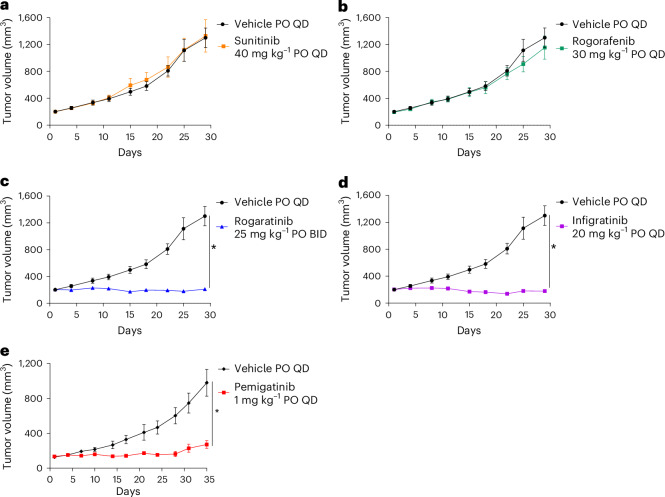
FGFR inhibition suppresses tumor growth in a PDX model of SDHd GIST^26^. The SDHd GIST PDX (PG20) was treated separately with either sunitinib (multi-targeted receptor tyrosine kinase inhibitor) (**a**), regorafenib (pan-kinase inhibitor) (**b**), rogaratinib (FGFRi) (**c**), infigratinib (FGFRi) (**d**) or pemigatinib (FGFRi) (**e**) and compared to vehicle control, with *n* = 8 mice per treatment group. Dosing was administered for 28–35 days with the indicated dosing schedules, and growth rates were evaluated by measuring tumor volume. Error bars represent s.e.m. *Significant growth rate difference with *P* < 0.001 as analyzed by a mixed-effect repeated-measurement linear model including a quadratic term for time and interaction between time and treatment. Statistical significance of the drug–time interaction terms was assessed using two-sided *t*-tests on fixed-effect coefficients from linear mixed-effects models (R package ImerTest, Satterthwaite degrees of freedom approximation), with mouse as a random effect (rogaratinib, *P* < 0.0001; infigratinib, *P* < 0.0001; pemigatinib, *P* < 0.0001). Panels without an asterisk denote a nonsignificant difference in growth rate (sunitinib, *P* = 0.8315; regorafenib, *P* = 0.0642). PO BID, by mouth twice daily; PO QD, by mouth once daily.

**Extended Data Fig. 2:**
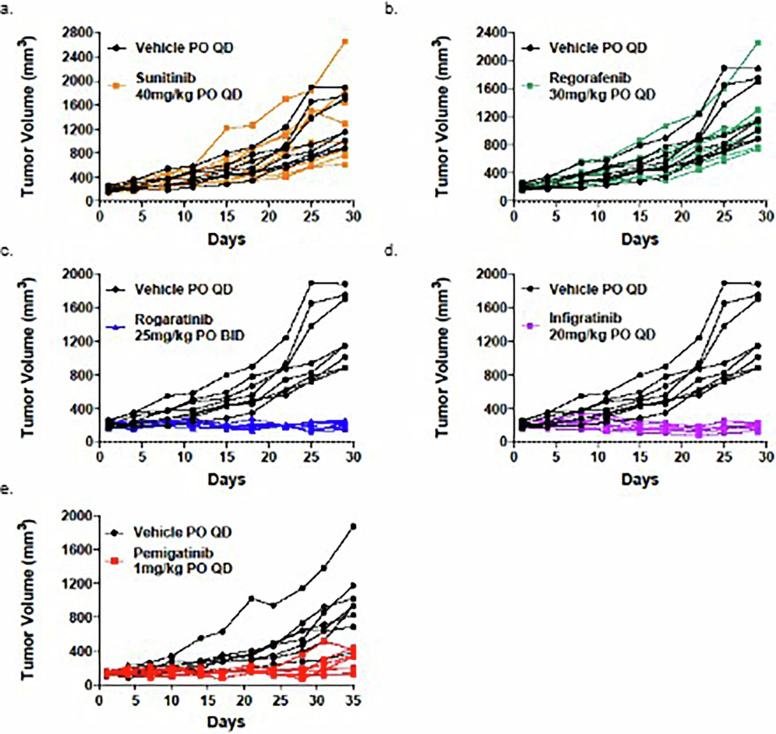
Spider plots of individual SDHd GIST PDX tumor growth from Fig. 5. The SDH-deficient GIST PDX (PG20) was treated separately with either **a**) sunitinib, **b**) regorafenib, **c**) rogaratinib, **d**) infigratinib, or **e**) pemigatinib and compared to vehicle control, with n=8 mice per treatment group. Dosing was administered for 28-35 days with the indicated dosing schedules, and growth rates were evaluated by measuring tumor volume. Tumor volumes were graphed over time, with each line representing a single mouse from Fig. 5.

**Extended Data Fig. 3:**
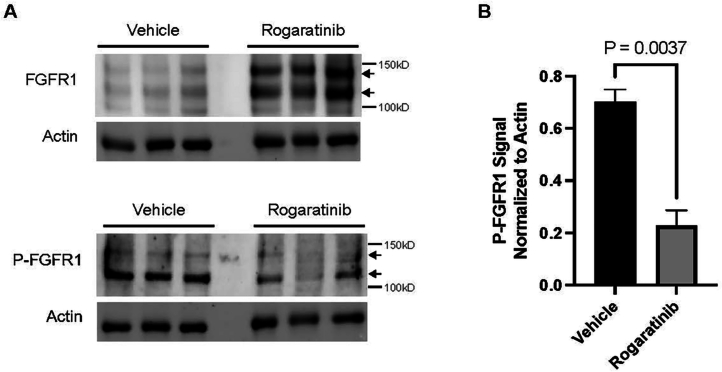
Pharmacodynamics of Rogaratinib in SDH-deficient GIST. **a**) Immunoblot of total FGFR1 (top) and phospho-FGFR1 (bottom) in SDH-deficient GIST PDX tumors 4hrs after last dose following 3 days of treatment with rogaratinib (50mg/kg BID) or vehicle. Each lane is protein lysate taken from a separate tumor. Arrows indicate FGFR1 isoforms at 145kD and 120kD. Immunoblots were repeated twice with similar results in each replicate. **b**) Quantification of phospho-FGFR1 signal intensity normalized to actin signal. Bars represent group means and standard errors. Source data

Our prior study suggests that FGFR dependency is a hallmark of SDHd GISTs due to their aberrant expression of FGF ligands^15^. Rogaratinib is a well-validated and highly selective FGFRi^36^. To evaluate FGFR inhibition as a class effect leading to tumor growth suppression in SDHd GIST, multiple FGFRi were evaluated in a patient-derived xenograft (PDX) model of SDHd GIST (Fig. 5, Extended Data Fig. 2). Pemigatinib (FGFR1–3 inhibitor), infigratinib (FGFR1–3 inhibitor) and rogaratinib (FGFR1–4 inhibitor) all demonstrated robust, statistically significant suppression of tumor growth (*P* < 0.0001). By contrast, the multi-targeted tyrosine kinase inhibitors sunitinib and regorafenib, which do not potently and selectively inhibit FGFRs, were comparable to vehicle control (Fig. 5). Rogaratinib-treated PDX tumors showed reduced phospho-FGFR1, confirming inhibition of FGFR signaling (Extended Data Fig. 3). These results provide strong evidence that aberrant FGF3/4–FGFR1/2 signaling in SDHd GISTs is the relevant target of rogaratinib in our trial. Our PDX studies further demonstrate that other small molecules targeting this signaling axis show preclinical efficacy and suggest likely clinical efficacy for the other FGFRi with potent activity against FGFR1 and FGFR2 that are approved or in clinical development.

## Discussion

Identification of the canonical gain-of-function *KIT* mutation in GIST led to the successful use of receptor tyrosine kinase inhibitors, representing one of the first targeted therapies for solid tumors^37,38^. However, KIT-directed targeted therapies have provided minimal clinical benefits in SDHd GISTs, with the exception of rare case reports and prolonged stable disease^4–7,38^. Efforts to target the unique biology of SDHd GISTs have also seen limited success. The insulin-like growth factor receptor 1 (IGF-1R) inhibitor linsitinib failed to show objective responses despite preclinical work suggesting high IGF-1R expression in SDHd GIST^8,39–41^. The DNA methyltransferase inhibitor guadecitabine, with potential to reverse the characteristic hypermethylation in SDHd GIST, also failed to yield objective responses^9^. The tyrosine kinase inhibitor imatinib was studied in combination with the MEK inhibitor binimetinib to target ETV1, a transcription factor required for growth and survival of GIST, and results showed one response of five patients with SDHd, with a median PFS of 45 months^10^. A recent ongoing study of temozolomide in SDHd GIST (NCT03556384) has completed accrual. Preliminary results reported an objective response of 17%, suggesting benefit in a subset of patients, although final results and correlative analysis have not yet been reported^42^. The objective responses observed with MEK inhibition and with temozolomide are interesting because they also may exploit epigenetic alterations in SDHd GIST.

The recognition that DNA methylation changes are widespread in human cancers and likely have causal roles in at least a subset of tumors has prompted extensive interest in epigenetic therapies that target or correct such lesions. DNA methyltransferase inhibitors have shown efficacy in subsets of hematologic malignancies^43^, but these agents, when used as monotherapies in trials in solid tumors, have yet to yield meaningful clinical benefits^44^. Inhibitors of mutant isocitrate dehydrogenase have shown benefit in certain leukemias and gliomas, potentially by moderating the excess methylation in these tumors^45–47^. The clinical utility of methylation biomarkers has also been explored, with the most prominent clinical examples being methylation-based silencing of the O^6^-methylguanine-DNA methyltransferase as an indication for temozolomide chemotherapy in glioblastoma^48^. In principle, methylation-based activation of proto-oncogenes—as described for *FGF* ligand genes in SDHd GIST and *PDGFRA* in hypermethylated isocitrate dehydrogenase-mutant gliomas^15,16^—could yield biomarkers for targeted therapy, but examples have not been forthcoming.

The current study helps address this gap by demonstrating the efficacy of targeted FGFR therapy in a tumor subset in which DNA hypermethylation and insulator disruption drive FGF ligand expression and autocrine signaling. Tumor sequencing confirmed that all available tumors in the trial cohort overexpressed FGF4 along with multiple FGFRs but lacked canonical oncogene mutations. In this phase 2 study, 10 of 24 patients with SDHd GIST treated with rogaratinib demonstrated confirmed PR, for an objective response of 41.7%. Disease control was remarkably prolonged, with nine patients remaining on treatment, seven of whom continue to have PR, and the upper limit of the CI for median PFS has not yet been reached. Although the PFS is based on a small number of events, 12 patients (50%) remained on study for at least 1 year, and 4 have remained on study beyond 2 years. Objective response rates and durability of responses in this study exceed outcomes previously reported in SDHd GIST^4–10,42,49^. Promising results from the report of a small number of patients with SDH deficiency in a phase 1 trial who were treated with the multi-targeted agent olverembatinib with activity against FGFR1 showed a PR of 23% in 6 of 26 participants^50^, and a retrospective report of 3 of 3 patients experiencing PR when treated with the FGFR1–4 inhibitor lenvatinib^51^ further validate the results of this current phase 2 trial. We further note that a fourth GIST subtype (quadruple wild-type) has been shown to overexpress *FGF4* due to focal duplication rather than epigenetic changes, further supporting the FGFR pathway as a relevant pathway in some subsets of GIST^52^.

Pharmacokinetic assays demonstrated drug exposure variability, which was much higher than in previous reports and may be more reflective of real-world exposure due to the prolonged nature of sampling in our study. FGFRi such as erdafitinib, pemigatinib and futibatinib have reported associations with diarrhea, hyperphosphatemia, nail toxicity and dry eyes, which we specifically evaluated in addition to overall toxicity grading and tumor response as a function of exposure^53^. None of the exposure toxicity relationships appeared to be detectable in our data (Fig. 3). However, a serum phosphate increase was clearly associated with rogaratinib treatment, and therefore demonstrated pharmacodynamic confirmation that rogaratinib treatment in our study engaged FGFR1. The increase in serum phosphate is indeed a class effect also seen with erdafitinib, pemigatinib and futibatinib^28^. In an erdafitinib study, baseline serum phosphate levels were higher in women, but this was not the case in our dataset^54^.

We note limitations of our study and opportunities for further investigation. First, we observed toxicities in this study, consistent with reports of other FGFRi^28,55^, although these were generally manageable through dose reductions and did not appear to compromise the ability of patients to remain on treatment long-term nor treatment efficacy. Rates of dose reduction and treatment discontinuation were comparable to other FGFR trials, where dose reduction rates >50% are common^24,55–57^. Second, we were unable to identify specific SDH subunit alterations, other mutations or markers that were predictive of a rogaratinib response, which may indicate that patients with SDHd GIST benefit from FGFR inhibition irrespective of specific mutational status (Fig. 2a,b). The two tumors in our study with primary PD harbored unique genetic alterations associated with aggressive tumor phenotypes, including one with chromosome 5p gain^32^ and another with concurrent *TP53* and *ATRX* loss-of-function mutations^33–35^, which may represent important features for future study. Also of note, patients with SD had received a slightly higher number of prior therapies (mean = 1.67) than patients achieving PR (mean = 1.1), although again conclusions are limited by sample size. Third, although we confirmed coincident expression of FGF ligands and receptors in all evaluable patients in our trial, we did not assess methylation patterns but rather relied on the standard SDHB IHC biomarker for inclusion. Finally, while these expression correlates and our preclinical PDX testing of a range of therapeutic agents strongly support a class effect for FGFRi in SDHd GIST, further clinical evaluations with commercially available FGFRi are essential to build on our findings for rogaratinib and evaluate other agents in the clinic. This is of particular importance because rogaratinib is no longer being developed for clinical use.

Our study represents a prospective multicenter therapeutic trial of patients with SDHd GIST. The results illustrate the importance of aberrantly activated FGFR signaling in this hypermethylated GIST subtype. Although direct comparisons across clinical trials are imperfect, the activity signal in this trial is favorable when viewed in the context of previously published trials of alternative treatment strategies in SDHd GIST. In addition to supporting FGFR inhibition as a promising approach for the management of SDHd GIST, our trial demonstrates that an epigenetic mechanism of oncogene activation can be effectively targeted by a receptor tyrosine kinase inhibitor. This approach is distinct from epigenetic therapies that directly target DNA methyltransferases or chromatin-modifying enzymes in hematologic cancers^1^. It is complementary to existing and emerging methylation-based indications predicated on DNA repair deficiencies or synthetic lethal interactions^1^. Given the frequency of methylation changes in human cancers, we anticipate that acquisition of methylation profiles for tumor cohorts at greater scale will identify and prioritize additional aberrations that activate targetable oncogenic programs or otherwise confer specific therapeutic vulnerabilities.

## Methods

### Inclusion and ethics

All patients provided written informed consent before enrollment. This protocol was conducted according to Good Clinical Practice and was consistent with the principles of the Declaration of Helsinki. The protocol, informed consent and subsequent amendments were reviewed and approved by the Central Institutional Review Board (IRB) of the National Cancer Institute (NCI IRB no. 3—Adult CIRB—Early Phase Emphasis, ID IRB00009430), with additional approvals at each site as required by local requirements. A complete list of IRB names and identifiers for all sites is available in the Supplementary Information. A data safety monitoring board was not involved in oversight of the trial. Trial monitoring and auditing was per NCI-Cancer Therapy Evaluation Program UM1 standard practice with Theradex. The principal investigator provided study oversight over all aspects of trial conduct.

### Eligibility criteria

Inclusion criteria were:

1. Patients must have locally advanced or metastatic disease that is not amenable to surgery.
2. Patients must have measurable disease.
3. Participant must be willing to undergo pre-treatment biopsy if disease site is amenable to biopsy and low risk for the biopsy procedure. If biopsy is not possible, eligibility may be approved after discussion with the study chair.
4. Age ≥18 years.
5. ECOG performance status ≤2.
6. Patients must have adequate organ and marrow function as defined below:
  Hemoglobin ≥ 8.0 g dl^−1^
  Absolute neutrophil count ≥1,000 per μl
  Platelets ≥100,000 per μl
  Total bilirubin ≤1.5× institutional upper limit of normal (ULN)
  AST(serum glutamic-oxaloacetic transaminase (SGOT))/ALT(serum glutamic-pyruvic transaminase (SGPT)) ≤ 3.0× institutional ULN (unless liver metastases are present in which case it must be ≤5× ULN)
  Glomerular filtration rate ≥60 ml per min per 1.73 m^2^.
7. Human immunodeficiency virus-infected patients on effective non-CYP3A4 interacting anti-retroviral therapy with undetectable viral load within 6 months are eligible for this trial.
8. For patients with evidence of chronic hepatitis B virus infection, the hepatitis B virus viral load must be undetectable on suppressive therapy, if indicated.
9. Patients with a history of hepatitis C virus (HCV) infection must have been treated and cured. For patients with HCV infection who are currently on treatment, they are eligible if they have an undetectable HCV viral load.
10. Patients with treated brain metastases are eligible if follow-up brain imaging after central nervous system-directed therapy shows no evidence of progression.
11. Patients must be disease-free of prior invasive malignancies for >5 years with the exception of curatively treated basal cell or squamous cell carcinoma of the skin or carcinoma in situ of the cervix. Note that if there is a history of prior malignancy, patients must not be receiving other specific treatment for that cancer.
12. Patients should have completed prior treatment for their cancer: chemotherapy or radiotherapy must have been completed for >2 weeks (6 weeks for nitrosoureas or mitomycin C) before entering the study.
13. Patients should have recovered from AEs due to prior anti-cancer therapy (that is, have residual toxicities > Grade 1) with the exception of alopecia.
14. Patients with known history or current symptoms of cardiac disease, or history of treatment with cardiotoxic agents, should have a clinical risk assessment of cardiac function using the New York Heart Association Functional Classification. To be eligible for this trial, patients should be class 2B or better.
15. Patients must have a corrected QT (QTc) interval length <450 ms.
16. Participant is willing to comply with the protocol for the duration of the study including undergoing treatment and scheduled visits and examinations including follow-up.
17. Participant must be able to swallow and maintain pills.
18. Women of childbearing potential must have a negative urine or serum pregnancy test within 28 days of initial dose of rogaratinib (BAY 1163877), and again within 7 days before treatment on day 1. If screening occurs within 7 days of day 1, only one pregnancy test is required.
19. The effects of rogaratinib (BAY 1163877) on the developing human fetus are unknown. For this reason and because kinase inhibitor agents are known to be teratogenic, women of childbearing potential and men must agree to use adequate contraception (hormonal or barrier method of birth control; abstinence) before study entry, for the duration of study participation, and 4 months after completion of rogaratinib (BAY 1163877). Should a woman become pregnant or suspect she is pregnant while she or her partner is participating in this study, she should inform her treating physician immediately. Men treated or enrolled on this protocol must also agree to use adequate contraception before the study, for the duration of study participation, and 4 months after completion of rogaratinib (BAY 1163877) administration.
20. Ability to understand and the willingness to sign a written informed consent document. Participants with impaired decision-making capacity who have a legally authorized representative and/or family member available will also be eligible.

Exclusion criteria included:

1. Patients who are receiving any other investigational agents.
2. History of allergic reactions attributed to compounds of similar chemical or biologic composition to rogaratinib (BAY 1163877).
3. Concomitant administration with sensitive substrates or narrow therapeutic index drugs of CYP3A4, P-glycoprotein BCRP, MATE1 and MATE2K, and strong inhibitors and inducers of CYP3A4 should be avoided. Use caution with strong inhibitors and inducers of P-glycoprotein. Because the lists of these agents are constantly changing, it is important to regularly consult a frequently updated medical reference. As part of the enrollment and informed consent procedures, the patient will be counseled on the risk of interactions with other agents, and what to do if new medications need to be prescribed or if the patient is considering a new over-the-counter medicine or herbal product.
4. Concomitant administration of medications that prolong the QT/QTc interval is prohibited in accordance with the published US Food and Drug Administration guidance ‘E14 Clinical Evaluation of QT/QTc Interval Prolongation and Proarrhythmic Potential for Non-Antiarrhythmic Drugs’.
5. Patients with disturbed calcium and/or phosphate metabolism are excluded from this study.
6. Patients with uncontrolled intercurrent illness.
7. Patients with psychiatric illness or social situations that would limit compliance with study requirements.
8. Pregnant women are excluded from this study because rogaratinib (BAY 1163877) is kinase inhibitor agent with the potential for teratogenic or abortifacient effects. Because there is an unknown but potential risk for AEs in nursing infants secondary to treatment of the mother with rogaratinib (BAY 1163877), breastfeeding should be discontinued if the mother is treated with rogaratinib (BAY 1163877).

### Study design, treatment and endpoints

This is a multicenter, open-label, two-stage phase 2 study to evaluate the efficacy and safety of rogaratinib in patients with sarcoma. Two cohorts were defined: cohort A, patients with advanced sarcoma that harbored an alteration in FGFR1–4; and cohort B, patients with SDHd GIST (Clinicaltrials.gov identifier: NCT04595747). The cohorts were enrolled in parallel with separate analysis. This paper reports cohort B the SDHd GIST cohort. The trial was conducted at 11 centers in the USA participating in the NCI-Cancer Therapy Evaluation Program Experimental Therapeutics Clinical Trials Network (ETCTN). Patients received rogaratinib (BAY 1163877) 800 mg by mouth twice daily continuously in 28-day cycles.

The primary objective was to estimate objective response rate. Secondary objectives were to estimate PFS, and safety and tolerability. Exploratory objectives were to evaluate serial measurements of *FGF3* and *FGF4* and FGFR in serial biopsies, to perform whole-exome sequencing in serial biopsies, and to explore rogaratinib with pharmacodynamic effects.

Dose reductions were permitted by one dose level to 600 mg twice daily or up to two dose levels to 400 mg twice daily based on toxicities. Patients unable to tolerate the lowest dose level or who required a dose interruption of more than 21 days due to toxicities were taken off protocol therapy.

Patients were followed for 30 days after removal from study until disease progression, unacceptable AE or change in clinical condition, patient noncompliance or withdrawal from the trial. Patients removed from study for unacceptable AE(s) were followed until resolution or stabilization of the AE.

The primary endpoint was radiographic response by RECIST 1.1 to single-agent rogaratinib. Tumor assessments were performed using either computed tomography scan or magnetic resonance imaging at baseline, then every 8 weeks for the first year, then every 12 weeks thereafter. Confirmatory scans were required 8 weeks (at a minimum of 4 weeks) following first documentation of objective response by RECIST 1.1.

Secondary endpoints were PFS and safety and tolerability. AEs were graded using the NCI Common Terminology Criteria for Adverse Events v.5.0.

Exploratory objectives were to evaluate the relationship between genomic, transcriptomic, proteomic or epigenomic features of archival, pre-treatment and any post-progression samples to outcomes.

Across our studies, sex was not considered to be a biological variable impacting SDHd GIST cancer. The study design did not limit participants based on sex. Both male and female participants were included. Sex was reported by the study team into the central database. Data were not disaggregated based on sex and gender. No sex- or gender-based analyses were planned a priori.

### Statistical design

The trial had a Simon two-stage optimal design, targeting at least a 20% improvement in objective response rate, from a historical control rate of 5% to 25% or higher, with parameters including a one-sided type 1 error of 10%, and a power of 90%. The two-stage structure required one or more responses in the first nine patients to proceed to the second stage, serving as an early futility checkpoint to halt the study if the treatment showed no early signs of activity. If this initial threshold was met, enrollment would continue to a total accrual of 24 patients in the second stage. The final efficacy endpoint required responses in 3 or more of the total 24 enrolled patients to declare the trial positive. The actual power achieved by this setting was 90.3%, with an actual type 1 error of 9.3% and a probability of stopping at the first stage equal to 63%. Time on treatment was defined as time from enrollment until date off treatment or data cut. All patients initiated study drug within 7 days of the enrollment date, except one patient who started study drug 10 days after enrollment. A standard Kaplan–Meier estimator was calculated to plot the PFS curve, including 95% CI. Correlative and biomarker objectives were considered exploratory.

### Protocol amendments

- Amendment 1 (v.02Aug2021): clarified that timing of all visits and assessments tied to C1D1, and added a window for scheduling assessments. Height was removed as a screening parameter.
- Amendment 2 (v.18Jan2022): revised protocol to broaden the eligibility criteria to allow a subset of participants to enroll without a disease site considered accessible and low risk for biopsy.
- Amendment 3 (v.30Jun2022): revised consent form to include additional drug risk of hyperphosphatemia (increased levels of phosphorous in the blood).
- Amendment 4 (v.25Oct2022): eliminated the limit of 24 cycles of treatment and removed the secondary objective to estimate overall survival.
- Amendment 5 (v.07Mar2023): decreased frequency of labs beyond cycle 4.
- Amendment 6 (v.26Jul2023): permitted use of archival tissue if pre-treatment biopsy unavailable and revised consent form to explain the use of archival tissue.

### Correlatives studies

A pre-treatment (before C1D1) biopsy, if the disease site was amenable to biopsy, and post-progression (optional) tissue biopsy cores per patient were collected, flash frozen and sent to the Early Phase and Experimental Clinical Trials Biospecimen Bank (EET Biobank) for nucleic acid extraction and subsequent genomic and transcriptomic analysis at the National Clinical Laboratory Network genomics laboratory in the Molecular Characterization and Clinical Assay Development Laboratory, Frederick National Laboratory for Cancer Research. Nine of 13 pre-treatment biopsy samples met the >70% tumor content requirement necessary to proceed with genomic and transcriptomic analysis.

### Biospecimen nucleic acid extraction

DNA and RNA were extracted, and quality was assessed at the Biopathology Center at EET Biobank. RNA and DNA were extracted from tumors using a modification of the Qiagen AllPrep DNA/RNA Mini kit (cat. no. 80204). Homogenized and lysed tissue utilizing RLT buffer tissue was passed through the AllPrep DNA spin column. The flow-through from the Qiagen DNA column was processed using the Ambion mirVana microRNA isolation kit (cat. no. AM1560). This latter step generated RNA preparations that included RNA of <200 nucleotides suitable for miRNA analysis. The DNA spin column continued utilizing the Qiagen AllPrep kit for DNA isolation. For germline DNA extraction from 10 ml of whole blood, the Qiagen QIAAmp DNA Blood Maxi kit (cat. no. 51192) was utilized.

RNA samples were quantified by measuring fluorescence by Qubit Fluorometer utilizing the Qubit RNA BR assay kit (cat. no. Q10210), and DNA quantified by Quant-iT PicoGreen assay (cat. no. P7589). DNA specimens were resolved by 1% agarose gel electrophoresis to confirm high molecular weight fragments. RNA was analyzed via the RNA6000 Nano assay (Agilent, cat. no. 5067-1529) for determination of the RNA Integrity Number.

### Genomic analysis

Mutations and fusions in *FGFR1–4* were examined pre-enrollment by clinical sequencing. Tumor DNA samples from pre-treatment biopsies of nine patients were analyzed by whole-exome sequencing. Local standard of care genomic testing on archival tissue was included in the genomic analysis, where available.

### Whole-exome sequencing

Exome analysis used a minimum of 50 ng of DNA from tumor and germline collections at the pre-treatment collection time point. The NLCN whole-exome sequencing assay began with initial library preparation using the Kapa HyperPrep Plus protocol. After a pre-hybridization polymerase chain reaction (PCR) and bead-based library purification, the Agilent SureSelect v6 + CoSMiC exome workflow was used for exome hybridization and capture, followed by enrichment PCR. A custom exome panel was used, which covers ~42 Mb of the human exome and focuses on clinically actionable cancer-associated genes. The custom panel includes higher coverage in selected genes to provide increased sensitivity for single-nucleotide variants and insertions and deletions in actionable genes. The panel detects single-nucleotide variants, insertions and deletions, and copy number variations.

### RNA sequencing

Bulk RNA-seq was performed with pre-treatment tumor biopsies from nine patients. RNA-seq libraries were prepared by the NLCN RNA-seq assay with 100 ng of total RNA input using the Illumina TruSeq RNA Exome Dual Index library preparation kit, which covers ~45 Mb of the coding transcriptome. Libraries were quantitated with the Thermo Fisher Quant-iT DNA fluorescence-based quantitation kit before pooling and by Bio-Rad Droplet Digital PCR subsequently before being loaded on to an Illumina NovaSeq S4 flow cell. The resulting data were then demultiplexed and analyzed via the Molecular Characterization and Clinical Assay Development Laboratory RNA-seq bioinformatics pipeline. The RNA-seq pipeline detects RNA fusions and filters out small noncoding RNA and residual ribosomal RNA, leaving only reads from messenger RNA.

### Bioinformatics

For bioinformatics data processing, the hg19 reference genome was used. From whole-exome sequencing, BWA was used for sequence alignment, and the SDHA and other mutations were identified by the MuTect2 software^58^. The copy number calls were inferred by Sequenza^59^. For the gene expression analysis, alignment using STAR was performed, and normalized counts were obtained from RSEM^60^ and DESeq2^61^ results.

### SDH status

SDH subunit IHC was performed pre-enrollment. Pathology reports were analyzed for evidence of SDHB or SDHA expression using IHC. Genomic mutation or copy number loss of SDH complex genes were identified from standard of care genomic sequencing or whole-exome sequencing of pre-treatment biopsies.

### Pharmacokinetics

Patients were sampled for EDTA plasma pharmacokinetics of rogaratinib pharmacokinetic before treatment and before their daily dose on cycle 1 day 15, cycle 2 day 1 and day 15, and cycle 3 day 1. Rogaratinib concentrations were quantitated with a validated liquid chromatography coupled with tandem mass spectrometry method over a range of 50–10,000 ng ml^−1^. Each blood sample was centrifuged at approximately 1,000*g*, and plasma was stored at −70 °C or colder until analysis.

Rogaratinib was purchased from MedChemExpress and [D4]-rogaratinib was obtained from Bayer AG. Acetonitrile and water (both high-performance liquid chromatography grade) were purchased from Fisher Scientific. Formic acid was purchased from Sigma-Aldrich. Control EDTA human plasma was purchased from BioIVT. Nitrogen for mass spectrometric applications was purified using a nitrogen generator (Parker Balston). The liquid chromatography system consisted of a Waters ACQUITY UPLC system and an Acquity UPLC BEH C18 (2.1 × 50 mm, 1.7 μm) column, kept at ambient temperature. The autosampler temperature was maintained at 5 °C. Mobile phase solvent A was 0.1% formic acid in water, and mobile phase solvent B was 0.1% formic acid in acetonitrile. A flow rate of 0.2 ml min^−1^ was maintained throughout the run. The initial mobile phase composition was 85% solvent A, which decreased linearly to 0% over 1 min. Between 1 and 2 min, the mobile phase composition was held constant, and at 2.1 min, solvent A was increased to 85%, where it was held constant until 4 min, followed by two injections of the solvent (50% methanol in water (v/v)). The total run time was 4 min, with an injection volume of 5 μl. The approximate retention time of rogaratinib was 1.52 min, with a corresponding capacity factor of 2.37, at a void time of 0.45 min. Mass spectrometric detection was carried out using an AB SCIEX TRIPLE QUAD 5500 tandem mass spectrometer with electrospray ionization in positive multiple reaction monitoring mode. The scanning parameters for the mass spectrometer in positive mode were as follows: curtain gas 20, collision gas 7, IonSpray voltage 5,500 V, probe temperature 450 °C, GS1 30, GS2 50, DP of 60 V, EP of 10 V, and CXP of 16 V. The CE was set at 15 V, except for the qualifier transition of rogaratinib (33 V). The multiple reaction monitoring transitions monitored were: *m*/*z* 467.1 > 367.1 for rogaratinib; 471.1 > 367.1 for rogaratinib internal standard (IS). The standard curve of ratio response (analyte peak area to IS peak area) versus concentration was plotted using quadratic regression with 1/*x*^2^ weighting. A 2 mg ml^−1^ rogaratinib stock solution was used to prepare a human plasma calibration range for rogaratinib from 0.05 µg ml^−1^ to 10 µg ml^−1^. Quality control (QC) samples were prepared at QC low, mid and high concentrations of 0.15, 4 and 8 µg ml^−1^, respectively. A volume of 25 µl of the standard, QC, or sample plasma was pipetted into a 13 × 100 mm disposable borosilicate glass culture tube. Then, 200 μl of acetonitrile extraction solution with 150 ng ml^−1^ IS was added to each, followed by vortexing for 30 s. Samples were centrifuged at ~1,000*g* for 10 min. Supernatants were transferred to autosampler vials, and 5 µl was injected into the liquid chromatography–tandem mass spectrometry system. QC-based accuracies ranged from 94.9% to 114.5%. Intra- and inter-assay precisions were between 99.7% and 105.9%. Incurred sample reanalysis of 20 samples yielded the following results: 5% of samples had a difference larger than 20%, with a median difference of 4.7% and a median absolute difference of 4.7%.

Data were processed with WinNonlin (Certara). For exploration of exposure response relationships, cycle 1 day 15, and patient-level geometric mean concentration values were plotted as a function of the presence of: (1) at least possibly related grade ≥2 hyperphopsphatemia in cycle 1 or 2; (2) at least possibly related nail toxicity through cycle 4; (3) at least possibly related eye toxicity through cycle 4; (4) at least possibly related diarrhea through cycle 3; (5) at least possibly related maximum grade toxicity in cycle 1 or 2; (6) any grade 3 or four times grade 2 toxicities; and (7) best response. Week 3 serum phosphate concentration was defined as the earliest value available from day 20 and onwards. Statistical testing was performed with RStudio v.2024.12.1.

### PDX experiments

All animal experiments were conducted at Dana-Farber Cancer Institute with the approval of the Institutional Animal Care and Use Committee in an AAALAC accredited vivarium. Mice were housed in a pathogen-free facility under a 12-h light and 12-h dark cycle, with ambient temperature maintained at 22.2 ± 1.1 °C and relative humidity at 35–55%, with food and water provided ad libitum. The PDX model, PG20, was generated from surgical resection tissue of a patient with SDHd GIST as described previously^15^. For efficacy studies, tumor fragments were implanted into 8-week-old female NOD.Cg-*Prkdc*^scid^ *Il2rg*^t^^m1Wjl^/SzJ (NSG) mice purchased from The Jackson Laboratory (stock no. 005557). Tumors were allowed to establish to 176.2 ± 45.3 mm^3^ in size before randomization into various treatment groups (Studylog software) with *n* = 8 per group as: vehicle control; 40 mg kg^−1^ once daily sunitinib (LC Laboratories; 0.1 M citrate buffer, pH 4.5); 30 mg kg^−1^ once daily regorafenib (LC Laboratories; 34% propylene glycol, 34% polyethylene glycol 400, 12% Fluka F-68 and 20% water); 25 mg kg^−1^ twice daily rogaratinib (Bayer AG; 10% ethanol, 40% Solutol HS15, 50% water); 20 mg kg^−1^ once daily infigratinib (LC Laboratories; 50% of acetic acid/acetate buffer, pH 4.6 and 50% polyethylene glycol 300); or 1 mg kg^−1^ once daily pemigatinib (MedChemExpress, 5% N,N-dimethyl acetamide, 95% of 0.5% methyl cellulose in water). Mice were treated orally for 28–35 days with these agents. Growth rates were evaluated by measuring tumor volumes every 3–4 days. To evaluate whether treatment with an agent led to a significant growth rate difference (*P* < 0.001) compared to vehicle control, a mixed-effect, repeated-measurement linear model was used, including a quadratic term for time and an interaction term between time and drug. All animal experiments were conducted at Dana-Farber Cancer Institute with the approval of the Institutional Animal Care and Use Committee in an AAALAC accredited vivarium.

### Reporting summary

Further information on research design is available in the Nature Portfolio Reporting Summary linked to this article.

## Online content

Any methods, additional references, Nature Portfolio reporting summaries, source data, extended data, supplementary information, acknowledgements, peer review information; details of author contributions and competing interests; and statements of data and code availability are available at 10.1038/s41591-026-04376-9.

## Supplementary information

Supplementary Information Supplementary Tables 1 and 2 and redacted protocol active as of the data cut.

Reporting Summary

## Source data

Source Data Fig. 2a RECIST response: waterfall.

Source Data Fig. 2b Treatment duration: swimmer.

Source Data Fig. 2c Kaplan–Meier analysis of PFS.

Source Data Fig. 3 Pharmacokinetic exposure response assessment.

Source Data Extended Data Table 2 Treatment-related adverse events.

Source Data Extended Data Fig. 3 Uncropped gels.

## Figures and Tables

**Extended Data Table 1:**
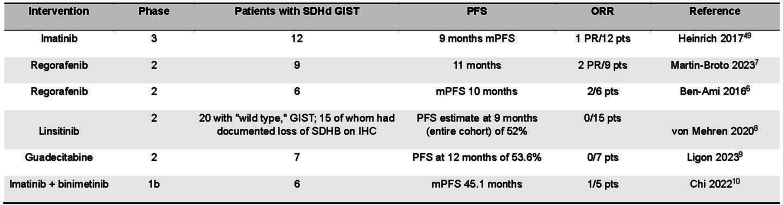
Comparison of progression-free survival (PFS) and objective response rate (ORR) across prior trials including SDHd GIST patients

**Extended Data Table 2:**
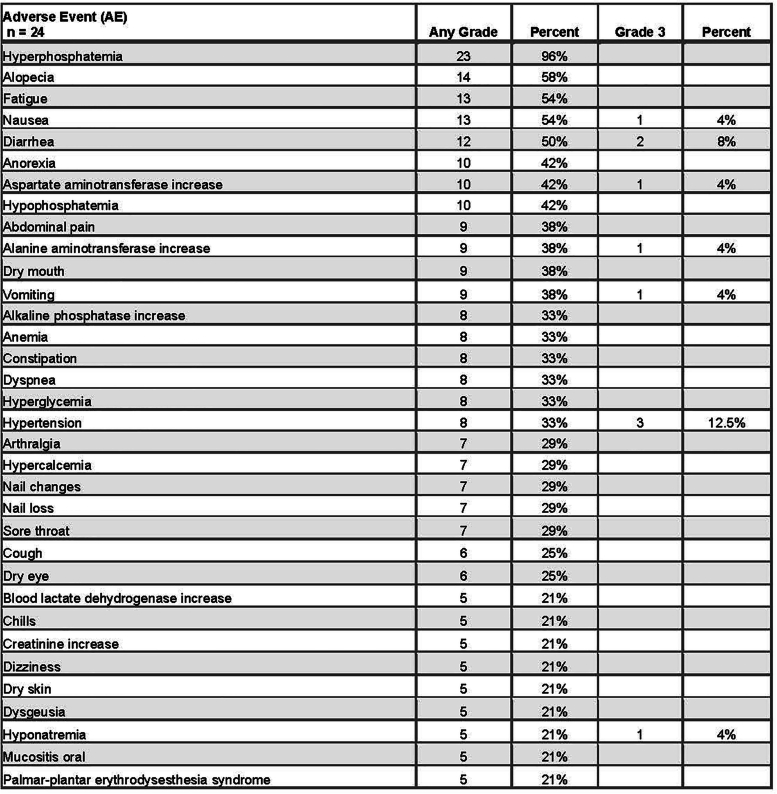
Treatment related adverse events in at least 20% of patients^7^

Source data

**Extended Data Table 3:**
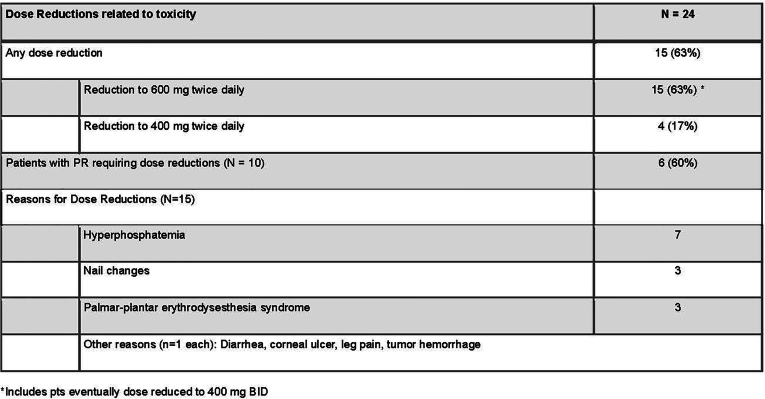
Drug Reductions and Response

**Extended Data Table 4:**
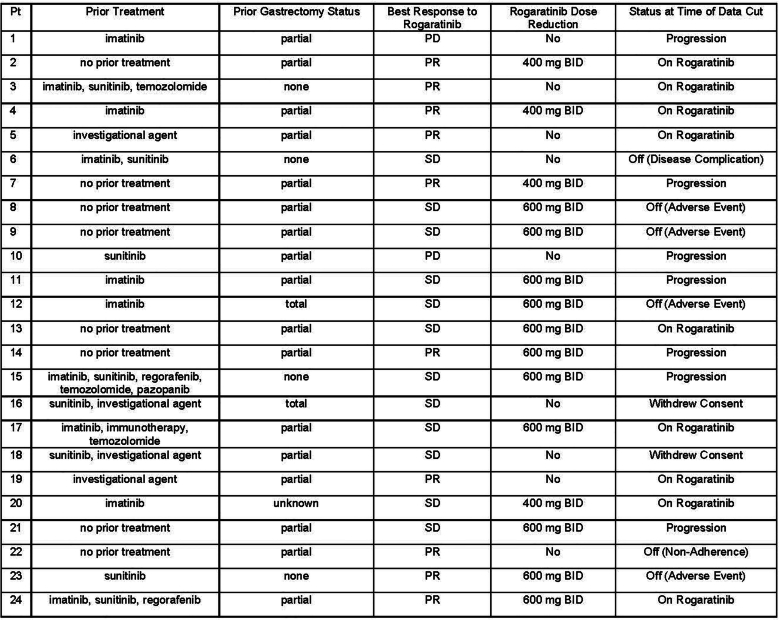
Clinical Patient Detail

## Data Availability

The study protocol can be found in the Supplementary Information. Original RNA-seq and whole-exome sequencing data generated from tumor specimens for patients on this clinical trial have been deposited in the database of Genotypes and Phenotypes (dbGaP) under accession phs004352. Identifiable patient data cannot be openly available due to confidentiality, but de-identified data requests may be directed to the corresponding authors (suzanne_george@dfci.harvard.edu and bradley_bernstein@dfci.harvard.edu). Requests will be reviewed for compliance with confidentiality restrictions with an estimated response time within 3 weeks. Clinical trial results will be submitted to clinicaltrials.gov. Source data are provided with this paper.
