# Supplementary material for: Fibroblast growth factor receptor inhibition for succinate dehydrogenase-deficient gastrointestinal stromal tumors: a phase 2 trial
Source: Nat Med. 2026 May 26;32(6):2191–200. doi: 10.1038/s41591-026-04376-9 (PMC13279270; doi:10.1038/s41591-026-04376-9)
Supplement: Supplementary file 1 — Supplementary Tables 1 and 2 and redacted protocol active as of the data cut. [file 41591_2026_4376_MOESM1_ESM.pdf]

nature medicine

Supplementary information https://doi.org/10.1038/s41591-026-04376-9

Fibroblast growth factor receptor inhibition for succinate dehydrogenase-deficient gastrointestinal stromal tumors: a phase 2 trial

In the format provided by the authors and unedited

Supplementary Table 1. All treatment related adverse events

Adverse Event (AE) N = 24	Any Grade	Percent	Grade 3	Percent Grade 3
**hyperphosphatasemia**	23	96%	0	0%
**alopecia**	14	58%	0	0%
**fatigue**	13	54%	0	0%
**nausea**	13	54%	1	4%
**diarrhea**	12	50%	2	8%
**anorexia**	10	42%	0	0%
**aspartate aminotransferase increase**	10	42%	1	4%
**hypophosphatasemia**	10	42%	0	0%
**abdominal pain**	9	38%	0	0%
**alanine aminotransferase increase**	9	38%	1	4%
**dry mouth**	9	38%	0	0%
**vomiting**	9	38%	1	4%
**alkaline phosphatase increase**	8	33%	0	0%
**anemia**	8	33%	0	0%
**constipation**	8	33%	0	0%
**dyspnea**	8	33%	0	0%
**hyperglycemia**	8	33%	0	0%
**hypertension**	8	33%	3	12.5%
**arthralgia**	7	29%	0	0%
**hypercalcemia**	7	29%	0	0%
**nail changes**	7	29%	0	0%
**nail loss**	7	29%	0	0%
**sore throat**	7	29%	0	0%
**cough**	6	25%	0	0%
**dry eye**	6	25%	0	0%
**blood lactate dehydrogenase increase**	5	21%	0	0%
**chills**	5	21%	0	0%
**creatinine increase**	5	21%	0	0%
**dizziness**	5	21%	0	0%
**dry skin**	5	21%	0	0%
**dysgeusia**	5	21%	0	0%
**hyponatremia**	5	21%	1	4%
**mucositis oral**	5	21%	0	0%
**palmar-plantar erythrodysesthesia syndrome**	5	21%	0	0%
**back pain**	4	17%	0	0%
**epistaxis**	4	17%	0	0%
**lymphocyte count decrease**	4	17%	1	4%

**memory impairment**	4	17%	0	0%
**muscle cramps**	4	17%	0	0%
**nail discoloration**	4	17%	0	0%
**non-cardiac chest pain**	4	17%	0	0%
**weight loss**	4	17%	0	0%
**blurred vision**	3	12%	0	0%
**covid 19**	3	12%	0	0%
**dehydration**	3	12%	0	0%
**dyspepsia**	3	12%	0	0%
**elevated alt**	3	12%	0	0%
**fever**	3	12%	0	0%
**flu-like symptoms**	3	12%	0	0%
**headache**	3	12%	0	0%
**hypokalemia**	3	12%	0	0%
**hypomagnesemia**	3	12%	0	0%
**investigations**	3	12%	0	0%
**nail ridging**	3	12%	0	0%
**neutrophil count decrease**	3	12%	0	0%
**pain**	3	12%	0	0%
**paronychia**	3	12%	1	4%
**peripheral sensory neuropathy**	3	12%	0	0%
**rhinorrhea**	3	12%	0	0%
**white blood cell decrease**	3	12%	0	0%
**alt elevation**	2	8%	0	0%
**arthritis**	2	8%	1	4%
**bone pain**	2	8%	0	0%
**chest pain**	2	8%	0	0%
**cognitive disturbance**	2	8%	0	0%
**constipation intermittent**	2	8%	0	0%
**diarrhea intermittent**	2	8%	0	0%
**dry eye/eyes**	2	8%	0	0%
**elevated ast**	2	8%	0	0%
**eye infection**	2	8%	0	0%
**floaters**	2	8%	0	0%
**gastroesophageal reflux disease**	2	8%	0	0%
**gastrointestinal disorders**	2	8%	0	0%
**hair color changes**	2	8%	0	0%
**hemorrhoids**	2	8%	0	0%
**hip pain**	2	8%	0	0%
**hyperkalemia**	2	8%	0	0%
**hypertension intermittent**	2	8%	0	0%

hypertriglyceridemia	2	8%	0	0%
hypocalcemia	2	8%	0	0%
hypoglycemia	2	8%	0	0%
hypotension	2	8%	0	0%
insomnia	2	8%	0	0%
localized edema	2	8%	0	0%
mucositis	2	8%	0	0%
myalgias	2	8%	0	0%
neck pain	2	8%	0	0%
oral mucositis	2	8%	0	0%
peripheral motor neuropathy	2	8%	0	0%
pruritus	2	8%	0	0%
rash acneiform	2	8%	0	0%
rash maculo-papular	2	8%	0	0%
skin and subcutaneous tissue disorders	2	8%	0	0%
strep throat	2	8%	0	0%
tinnitus	2	8%	0	0%
tumor hemorrhage	2	8%	2	8%
upper respiratory infection	2	8%	0	0%
urinary tract infection	2	8%	0	0%
weight gain	2	8%	0	0%
	1	4%	0	0%
back pain	1	4%	0	0%
abdominal pain - cramping intermittent	1	4%	0	0%
abdominal pain (acute)	1	4%	0	0%
acneiform	1	4%	0	0%
acneiform rash	1	4%	0	0%
alkaline phosphatase	1	4%	0	0%
allergic rhinitis	1	4%	0	0%
amnesia (mental fogginess)	1	4%	0	0%
ankle injury	1	4%	0	0%
ankle swelling	1	4%	0	0%
anxiety	1	4%	0	0%
arthralgia (elbow pain and plantar fascitis)	1	4%	0	0%
ast elevation	1	4%	0	0%
atelectasis	1	4%	0	0%
autoimmune disorder - erythema nodosum	1	4%	0	0%
bilater leg pain	1	4%	0	0%
bilateral age-related cataracts	1	4%	0	0%
bilateral eye discomfort	1	4%	0	0%
bilateral le edema	1	4%	0	0%

**bilateral leg pain**	1	4%	1	4%
**bilateral sholder/proximal humerus pain**	1	4%	0	0%
**bloating**	1	4%	0	0%
**bloating / early satiety**	1	4%	0	0%
**blood and lymphatic system disorders**	1	4%	0	0%
**blood bilirubin increase**	1	4%	0	0%
**blood in stool**	1	4%	0	0%
**bloodshot eye/eyes**	1	4%	0	0%
**blurred eye vision**	1	4%	0	0%
**blurred vision intermittent**	1	4%	0	0%
**bone pain (bilateral shin pain)**	1	4%	0	0%
**bone pain intermittent pain of ribs)**	1	4%	0	0%
**bradycardia**	1	4%	0	0%
**bruising**	1	4%	0	0%
**bug bites**	1	4%	0	0%
**chest pressure**	1	4%	0	0%
**chills intermittent**	1	4%	0	0%
**cholesterol high**	1	4%	0	0%
**choroidal folds**	1	4%	0	0%
**cold sore**	1	4%	0	0%
**cold symptoms**	1	4%	0	0%
**concentration impairment**	1	4%	0	0%
**corneal ulcer**	1	4%	1	4%
**creatinine increase (v5.0)**	1	4%	0	0%
**cystitis**	1	4%	0	0%
**decrease wbc**	1	4%	0	0%
**depressed mood**	1	4%	0	0%
**depression**	1	4%	0	0%
**depth perception changes**	1	4%	0	0%
**diplopia**	1	4%	0	0%
**dry nasal passages**	1	4%	0	0%
**dry skin to hands and feet**	1	4%	0	0%
**dry skin-hands**	1	4%	0	0%
**dysguesia, intermittent**	1	4%	0	0%
**dyspareunia**	1	4%	0	0%
**dyspenia**	1	4%	0	0%
**dysphagia**	1	4%	0	0%
**ear pain**	1	4%	0	0%
**edema (trunk)**	1	4%	0	0%
**edema limbs(rt middle finger edema)**	1	4%	0	0%
**elevated ldh**	1	4%	0	0%

**elevated serum creatinine**	1	4%	0	0%
**elevated total bilirubin**	1	4%	0	0%
**erectile dysfunction**	1	4%	0	0%
**erythema of hands**	1	4%	0	0%
**esophagitis**	1	4%	0	0%
**eye pain**	1	4%	0	0%
**eye redness**	1	4%	0	0%
**facial pain**	1	4%	0	0%
**fall**	1	4%	0	0%
**fatigue intermittent**	1	4%	0	0%
**feet and hand sensitivity**	1	4%	0	0%
**flank pain**	1	4%	0	0%
**flu**	1	4%	0	0%
**flu-like symptoms (documented + covid pcr)**	1	4%	0	0%
**folliculitis intermittent**	1	4%	0	0%
**gastro disorders**	1	4%	0	0%
**gastrointestinal disorders -other- blood in stool**	1	4%	0	0%
**general disorders and administration site conditions**	1	4%	0	0%
**gerd intermittent**	1	4%	0	0%
**hair color change**	1	4%	0	0%
**hair loss**	1	4%	0	0%
**hair texture abnormal**	1	4%	0	0%
**hair thinning**	1	4%	0	0%
**hand foot syndrome**	1	4%	0	0%
**headache intermittent**	1	4%	0	0%
**headaches**	1	4%	0	0%
**hematuria**	1	4%	0	0%
**hemorrhoidal hemorrhage**	1	4%	0	0%
**herpes simplex reactivation**	1	4%	0	0%
**high calcium**	1	4%	0	0%
**hip stiffness, achilles stiffness, pain in joints**	1	4%	0	0%
**hot flashes**	1	4%	0	0%
**hyperhidrosis**	1	4%	0	0%
**hyperphosphotemia**	1	4%	0	0%
**hypertrichosis**	1	4%	0	0%
**hypoalbuminemia**	1	4%	0	0%
**increase thirst**	1	4%	0	0%
**infection**	1	4%	0	0%
**intermittent elevated ast**	1	4%	0	0%
**irregular menstruation**	1	4%	0	0%

**joint aches**	1	4%	0	0%
**joint clicking**	1	4%	0	0%
**laceration left middle finger**	1	4%	0	0%
**ldh increase**	1	4%	0	0%
**left shoulder pain**	1	4%	0	0%
**libido decrease**	1	4%	0	0%
**light sensitivity**	1	4%	0	0%
**lip sensitivity**	1	4%	0	0%
**localized edema (ankles)**	1	4%	0	0%
**localized edema (right wrist)**	1	4%	0	0%
**lt middle finger injury**	1	4%	0	0%
**lymphocyte cell decrease**	1	4%	0	0%
**macular rash**	1	4%	0	0%
**macular-papular rash abdomen**	1	4%	0	0%
**memory impairement**	1	4%	0	0%
**mouth sores**	1	4%	0	0%
**mucositis oral mouth sores**	1	4%	0	0%
**mucositis oral- ulcer on tongue**	1	4%	0	0%
**mucositis oral/ mouth sores**	1	4%	0	0%
**muscle cramp intermittent**	1	4%	0	0%
**muscle cramping intermittent**	1	4%	0	0%
**muscle weakness**	1	4%	0	0%
**muscle weakness upper limb**	1	4%	0	0%
**myalgia**	1	4%	0	0%
**nail change**	1	4%	0	0%
**nail infection**	1	4%	0	0%
**nail lifting**	1	4%	0	0%
**nasal congestion**	1	4%	0	0%
**nausea intermittent**	1	4%	0	0%
**neuralgia**	1	4%	0	0%
**neuralgia (bilateral leg nerve pain)**	1	4%	0	0%
**neuralgia (feet)**	1	4%	0	0%
**neuropathy**	1	4%	0	0%
**neurosensory retinal detachmen**	1	4%	0	0%
**new palpable lesion to the left elbow**	1	4%	0	0%
**oral hemorrhage**	1	4%	0	0%
**oral pain**	1	4%	0	0%
**oral pain - tongue sensitivity with bump on tongue**	1	4%	0	0%
**otitis media**	1	4%	0	0%
**pain - secondary to renal calculi**	1	4%	0	0%

**pain (feet pain)**	1	4%	0	0%
**pain (right tibia)**	1	4%	0	0%
**pain in extremities (groin,hip knees)**	1	4%	0	0%
**pain in extremity**	1	4%	0	0%
**pain in extremity (r arm)**	1	4%	0	0%
**pain in extremity left hip**	1	4%	0	0%
**pain in extremity right arm**	1	4%	0	0%
**pain in extremity: left lower extremity**	1	4%	1	4%
**pain in extremity(foot)**	1	4%	0	0%
**partial gastric obstruction**	1	4%	1	4%
**periorbital edema**	1	4%	0	0%
**peripheral neuropathy**	1	4%	0	0%
**pharyngitis**	1	4%	0	0%
**photosensitivity**	1	4%	0	0%
**posterior vitreous detachment**	1	4%	0	0%
**presyncope**	1	4%	0	0%
**productive cough**	1	4%	0	0%
**psychaitric**	1	4%	0	0%
**rash**	1	4%	0	0%
**rash (bug bites)**	1	4%	0	0%
**rash-acneiform**	1	4%	0	0%
**rectal hemorrhage**	1	4%	0	0%
**red in corner of eye**	1	4%	0	0%
**reflux**	1	4%	0	0%
**renal calculi**	1	4%	0	0%
**renal calculi - left kidney**	1	4%	0	0%
**respiratory**	1	4%	0	0%
**retinopathy**	1	4%	0	0%
**right hip pain**	1	4%	0	0%
**right knee**	1	4%	0	0%
**right knee pain**	1	4%	0	0%
**right lower back pain**	1	4%	0	0%
**right tongue papule**	1	4%	0	0%
**scalp lesion**	1	4%	0	0%
**shingles**	1	4%	0	0%
**shortness of breath**	1	4%	0	0%
**sinus bradycardia**	1	4%	0	0%
**sinus bradycardio**	1	4%	0	0%
**sinusitis**	1	4%	0	0%
**skin infection**	1	4%	0	0%
**small bowel intusssusception**	1	4%	0	0%

**subconjunctival hemorrhage**	1	4%	0	0%
**sunburn - neck**	1	4%	0	0%
**surgical and medical procedure pain**	1	4%	0	0%
**taste alteration**	1	4%	0	0%
**taste changes**	1	4%	0	0%
**thrush**	1	4%	0	0%
**toe sensitivity**	1	4%	0	0%
**toe/fingernail sensitivity**	1	4%	0	0%
**tooth implant loose**	1	4%	0	0%
**toothache**	1	4%	0	0%
**tremor**	1	4%	0	0%
**uri**	1	4%	0	0%
**urinary tract pain**	1	4%	0	0%
**vaginal infection**	1	4%	0	0%
**vision decrease**	1	4%	0	0%
**vitamin d deficiency**	1	4%	0	0%
**vomiting (rel.to food poisoning)**	1	4%	0	0%
**watery eye/eyes**	1	4%	0	0%
**white blood cell count decrease**	1	4%	0	0%
**white blood cells decrease**	1	4%	0	0%
**worsened diarrhea**	1	4%	0	0%
**worsened dry eye syndrome, lacrimal ducts bilaterally**	1	4%	0	0%
**worsened dry eye/eyes**	1	4%	0	0%
**worsened elevated ast**	1	4%	1	4%
**worsened fatigue**	1	4%	0	0%
**worsened gerd with cough**	1	4%	0	0%
**worsened hair thinning**	1	4%	0	0%
**worsened muscle cramping - across lower back**	1	4%	0	0%

Supplementary Table 2. IRB Names and Identifiers of Participating Sites

CTEP_ID	IORG#	OHRP_ASSURANCE_NBR	IRB_NBR	NAME
CA043	IORG0000042	FWA00000692	IRB00009581	City of Hope National Med Ctr and the Beckman Rsch Inst IRB #2
			IRB00000060	City of Hope Natl Med Ctr & the Beckman Rsch Inst IRB#1
CO070	IORG0000433	FWA00023003	IRB00000648	Colorado- Panel A - Biomedical and Behavioral
			IRB00000650	Colorado Multiple Inst Review Board (COMIRB) - Panel B (Adult) - Biomedical and Behavioral
			IRB00000651	Colorado Multiple Inst Review Board (COMIRB) Panel C (Ped) - Biomedical and Behavioral
			IRB00002760	Colorado Multiple Inst Review Board (COMIRB) Panel D - Biomedical and Behavioral
			IRB00006846	Colorado Multiple Institutional Review Board (COMIRB) IRB #1 - Panel S - Behavioral
FL028	IORG0000153	FWA00002247	IRB00000260	U of Miami - Social & Behavioral Science Committee
			IRB00005621	U of Miami IRB #1 - Med IRB A
			IRB00005622	U of Miami IRB #2 - Med IRB B
			IRB00006078	U of Miami IRB #3 - Med IRB C
			IRB00010711	U of Miami IRB #5 - UM CIRB
MA036	IORG0000035	FWA00001121	IRB00000052	Dana-Farber Cancer Inst IRB #1 - PANEL A
			IRB00000753	Dana-Farber Cancer Inst IRB #2 PANEL 'B'
			IRB00001186	Dana-Farber Cancer Inst IRB #3 PANEL 'C'
			IRB00003340	Dana-Farber Cancer Inst IRB #4 PANEL 'D'
			IRB00005504	Dana-Farber Cancer Inst IRB #5 - PANEL 'E'
			IRB00006224	Dana-Farber Cancer Inst IRB #6 - PANEL 'F'
			IRB00007493	Dana-Farber Cancer Institute IRB #7 - PANEL 'G'
MI014	IORG0000144	FWA00004969	RB00000244	U of Michigan IRBMED A-1 #1
			IRB00000245	U of Michigan IRB #2 - Hlth - Health Sciences and Behavioral Sciences - Maize
			IRB00000246	U of Michigan IRB #3 - Behavioral Science - Health Sciences and Behavioral Sciences - Blue
			IRB00001995	U of Michigan IRBMED B-2 #6
			IRB00001996	U of Michigan IRBMED A-2 #7
			IRB00001999	U of Michigan IRBMED B-1 #8
			IRB00005467	U of Michigan IRB #1 - IRBMED C-1 #9
			IRB00012211	U of Michigan IRB #11 - IRBMED C-2 #11
MO011	IORG0000101	FWA00002284	IRB00005594	Washington U School of Medicine - Protocol Adherence Review Committee (PARC)
			IRB00009237	Washington U In St. Louis IRB #12 - WU IRB
NCIDTC	IORG0000010	FWA00005897	IRB00011862	National Institutes of Health IRB #10 - NIH Intramural IRB
			IRB00012078	National Institutes of Health IRB #9 - Research Compliance Review Committee
NY016	IORG0000163	FWA00004998	IRB00000273	MEMORIAL SLOAN-KETTERING CANCER CTR IRB #1
			IRB00009377	Memorial Sloan-Kettering Cancer Ctr IRB #2
			IRB00013747	Memorial Sloan Kettering Cancer Ctr IRB #3
OH007	IORG0000181	FWA00006378	IRB00000294	Ohio State U IRB #1 - Biomedical Science
			IRB00000295	Ohio State U IRB #2 - Behavioral & Social Science
			IRB00003096	Ohio State U IRB #3 - Biomedical Cancer
PA015	IORG0000196	FWA00006790	IRB00000319	U of Pittsburgh IRB #1 - A - IRB00000319
			IRB00000320	U of Pittsburgh IRB #2 - B - IRB00000320
			IRB00000321	U of Pittsburgh IRB #3 - C - IRB00000321
			IRB00003491	U of Pittsburgh IRB #6 - F - IRB00003491
			IRB00004005	U of Pittsburgh IRB #7 - G - IRB00004005
			IRB00004006	U of Pittsburgh IRB #8 - H - IRB00004006
			IRB00004728	IRB00004728 U of Pittsburgh IRB #9 – D
			IRB00012276	U of Pittsburgh IRB #5 - Committee E - EFIC IRB00012276
			IRB00012864	U of Pittsburgh IRB #4 - Committee I IRB00012864
			IRB00014780	U of Pittsburgh IRB #10 - Committee J
TX035	IORG0000083	FWA00000363	IRB00000121	U of Texas M.D. Anderson Cancer Ctr IRB #1 - Clinical - Clinical
NCI-CIRB	IORG0000460	N/A	IRB00009430	National Cancer Institute IRB #3 - Adult CIRB - Early Phase Emphasis
			IRB00000781	National Cancer Inst Central IRB #1 (Adult) - Adult CIRB - Late Phase Emphasis
			IRB00004296	National Cancer Inst Central IRB #2 (Pediatric)
			IRB00010018	National Cancer Institute IRB #4 - Cancer Prevention and Control

NCI Protocol #: 10411

Local Protocol #: 21-070

TITLE: Phase 2 Study of Rogaratinib (BAY 1163877) in the Treatment of Patients with Sarcoma Harboring Alterations in Fibroblast Growth Factor Receptor (FGFR) 1-4 and SDH-deficient Gastrointestinal Stromal Tumor (GIST)

Corresponding Organization: **LAO-MA036** / Dana-Farber - Harvard Cancer Center LAO

Principal Investigator: Suzanne George, MD
Sarcoma Center, Dana-Farber Cancer Institute

Participating Organizations

**LAO-11030** / University Health Network Princess Margaret Cancer Center LAO
**LAO-CA043** / City of Hope Comprehensive Cancer Center LAO
**LAO-CT018** / Yale University Cancer Center LAO
**LAO-MA036** / Dana-Farber - Harvard Cancer Center LAO
**LAO-MD017** / JHU Sidney Kimmel Comprehensive Cancer Center LAO
**LAO-OH007** / Ohio State University Comprehensive Cancer Center LAO
**LAO-PA015** / UPMC Hillman Cancer Center LAO
**LAO-TX035** / University of Texas MD Anderson Cancer Center LAO
**LAO-NCI** / National Cancer Institute LAO
**EDDOP** / Early Drug Development Opportunity Program
**CATCHUP** / Creating Access to Targeted Cancer Therapy for Underserved Populations

NCI-Supplied Agent: Rogaratinib (BAY 1163877) () (NSC 804782)

IND #: 153443
IND Sponsor: DCTD, NCI

Protocol Type / Version # / Version Date:

Original / July 22, 2020
Revision 1 / September 9, 2020
Revision 2a / November 18, 2020
Revision 3 / December 22, 2020
Revision 4 / January 14, 2021
Revision 5 / August 2, 2021
Revision 6 / January 18, 2022
Revision 7 / June 30, 2022
Revision 8 / October 25, 2022

Revision 9 / March 7, 2023
Revision 10 / July 26, 2023

SCHEMA

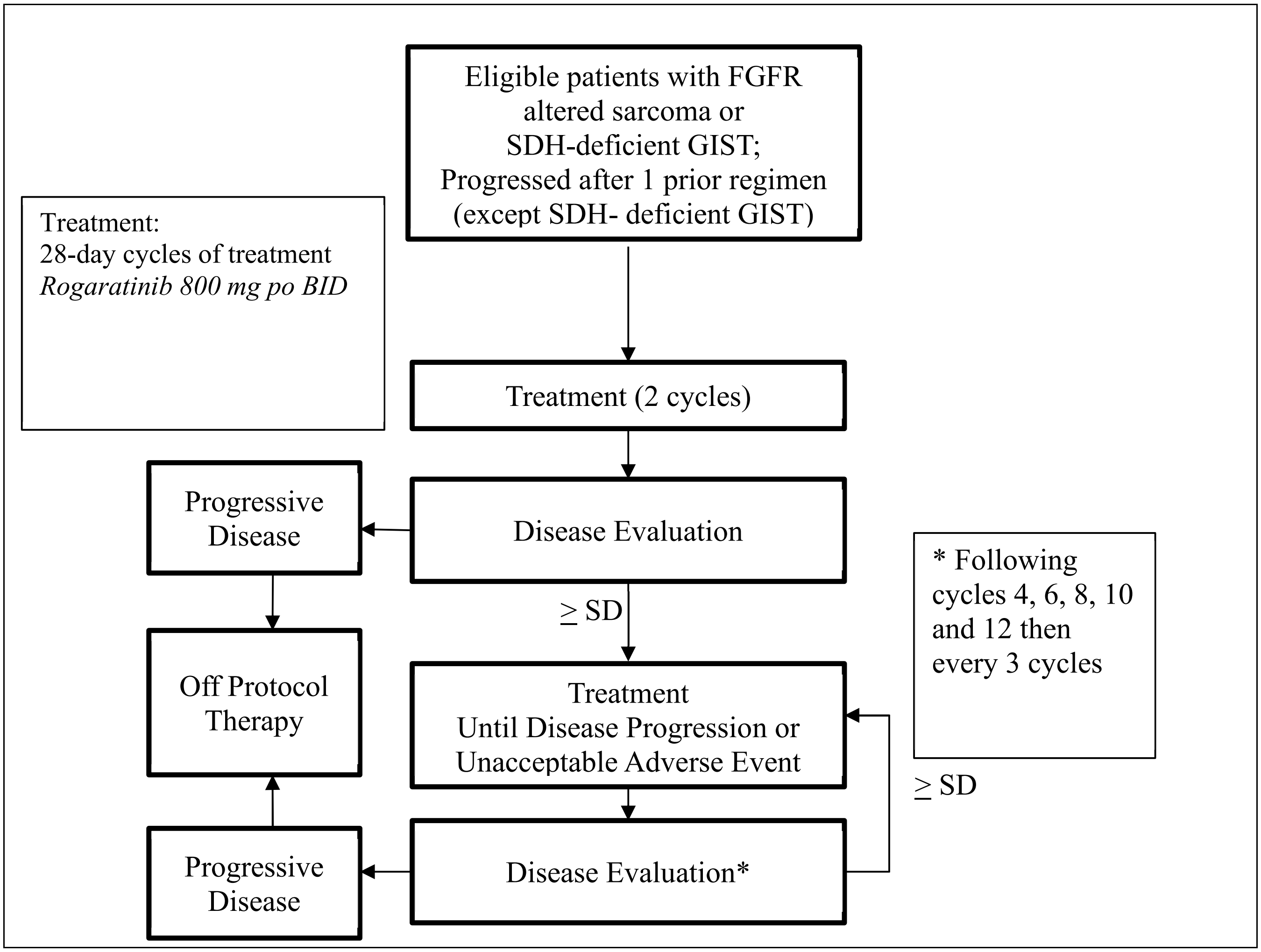
Eligible patients with FGFR altered sarcoma or SDH-deficient GIST; Progressed after 1 prior regimen (except SDH- deficient GIST)
Treatment:
28-day cycles of treatment
Rogaratinib 800 mg po BID
Treatment (2 cycles)
Progressive Disease
Disease Evaluation
≥ SD
Off Protocol Therapy
Treatment Until Disease Progression or Unacceptable Adverse Event
* Following cycles 4, 6, 8, 10 and 12 then every 3 cycles
≥ SD
Progressive Disease
Disease Evaluation*

TABLE OF CONTENTS

SCHEMA 3

1. OBJECTIVES 7
    1.1 Primary Objectives....7
    1.2 Secondary Objectives....7
    1.3 Exploratory Objectives....7

2. BACKGROUND....7
    2.1 Study Disease....7
    2.2 CTEP IND Agent....15
    2.3 Rationale....18
    2.4 Correlative Studies Background....22

3. PATIENT SELECTION....24
    3.1 Eligibility Criteria....24
    3.2 Exclusion Criteria....26
    3.3 Inclusion of Women and Minorities....27

4. REGISTRATION PROCEDURES....27
    4.1 Investigator and Research Associate Registration with CTEP....27
    4.2 Site Registration....28
    4.3 Patient Registration....31
    4.4 General Guidelines....33

5. BIOMARKER, CORRELATIVE, AND SPECIAL STUDIES....33
    5.1 Summary Table for Specimen Collection....33
    5.2 Summary Table(s) for Research Biopsies....34
    5.3 Specimen Procurement Kits and Scheduling....34
    5.4 Specimen Tracking System Instructions....35
    5.5 Specimen Collection....39
    5.6 Shipping Specimens from Clinical Site to the EET Biobank....41
    5.7 Shipping Specimens from Clinical Site to the JHU SKCCC Analytical Pharmacology Core Lab (APC) for the ChOP-KC....43
    5.8 Biomarker Plan....44
    5.9 Integral Laboratory Studies....47
    5.10 Integrated Correlative Studies....47
    5.11 Exploratory Correlative Studies....49

6. TREATMENT PLAN....50
    6.1 Agent Administration....50
    6.2 General Concomitant Medication and Supportive Care Guidelines....50
    6.3 Duration of Therapy....51
    6.4 Duration of Follow-Up....52

7. DOSING DELAYS/DOSE MODIFICATIONS....52

8. PHARMACEUTICAL INFORMATION 54
   8.1 CTEP IND Agent 55

9. STATISTICAL CONSIDERATIONS 57
   9.1 Study Design/Endpoints 57
   9.2 Sample Size/Accrual Rate 58
   9.3 Stratification Factors 58
   9.4 Analysis of Secondary Endpoints 59
   9.5 Analysis of Exploratory Endpoints 59
   9.6 For phase 2 protocols only: Reporting and Exclusions 59

10. ADVERSE EVENTS: LIST AND REPORTING REQUIREMENTS 60
   10.1 Comprehensive Adverse Events and Potential Risks List (CAEPR) 60
   10.2 Adverse Event Characteristics 62
   10.3 Expedited Adverse Event Reporting 62
   10.4 Routine Adverse Event Reporting 65
   10.5 Pregnancy 65
   10.6 Secondary Malignancy 66
   10.7 Second Malignancy 66

11. STUDY CALENDAR 66

12. MEASUREMENT OF EFFECT 69
   12.1 Antitumor Effect – Solid Tumors 69

13. STUDY OVERSIGHT AND DATA REPORTING / REGULATORY REQUIREMENTS 75
   13.1 Study Oversight 75
   13.2 Data Reporting 75
   13.3 Data Quality Portal 78
   13.4 CTEP Multicenter Guidelines 78
   13.5 Collaborative Agreements Language 78
   13.6 Genomic Data Sharing Plan 80
   13.7 Incidental/Secondary Findings Disclosure Procedure 80

14. REFERENCES 81

APPENDIX A PERFORMANCE STATUS CRITERIA 83

APPENDIX B FORMULA TO ESTIMATE RENAL FUNCTION USING SERUM CREATININE 84

APPENDIX C PATIENT DRUG INTERACTIONS HANDOUT AND WALLET CARD 85

APPENDIX D TISSUE BIOPSY VERIFICATION 88

APPENDIX E PATIENT’S MEDICATION DIARY 89

APPENDIX F PATIENT'S PHARMACOKINETIC WORKSHEET..........................91

1. OBJECTIVES

1.1 Primary Objectives

1.1.1 To estimate the objective radiographic response rate to single agent rogaratinib (BAY 1163877) in two cohorts of patients with sarcoma: Cohort A defined as patients with a sarcoma which harbors an alteration in fibroblast growth factor receptor (FGFR) 1,2,3 or 4 identified by next-generation sequencing profiling, and Cohort B defined as patients with advanced succinate dehydrogenase (SDH)-deficient gastrointestinal stromal tumor (GIST).

1.2 Secondary Objectives

1.2.1 To estimate progression-free survival (PFS) in patients in Cohort A and Cohort B treated with rogaratinib (BAY 1163877).

1.2.2 Further assessment for safety and tolerability.

1.3 Exploratory Objectives

1.3.1 To evaluate serial measurements of FGFR and FGFR ligand in serial tumor biopsies as potential pharmacodynamic markers of FGFR pathway inhibition by RNA-seq (pre-treatment biopsy and post-progression biopsy [if available]).

1.3.2 Whole exome sequencing (WES) of the pre-treatment biopsy and post-progression biopsy (if available) to help identify mechanisms of resistance.

1.3.3 To bank tumor material, germline deoxyribonucleic acid (DNA), and peripheral blood for potential future research for participating subjects who provide additional consent.

1.3.4 To explore rogaratinib exposure with pharmacodynamics effects (i.e., clinical response, toxicity, and markers of FGFR pathway inhibition).

2. BACKGROUND

2.1 Study Disease

Sarcomas represent a complex and heterogeneous group of mesenchymal malignancies arising from connective tissue in bone or soft tissue. There will be approximately 12-15,000 new cases of sarcoma each year in the United States, and 4-5,000 deaths (Siegel *et al.*, 2014). When localized, many sarcomas are potentially curable with surgical resection with or without radiation and chemotherapy (Brennan, 2013). Unfortunately, despite adequate local control, metastases are common and for most patients, advanced disease, whether at diagnosis or recurrence, is fatal. In metastatic soft tissue sarcomas (STS), single agent or combination chemotherapy has led to a median survival of 12 months across histologies (Italiano *et al.*, 2011). Standard single agent regimens for STS include doxorubicin (response rates <25%), ifosfamide

(response rates <25%), and other less active therapies including trabectedin, dacarbazine and platinum compounds. Combination regimens include doxorubicin plus ifosfamide (response rates 20- 24%), doxorubicin plus ifosfamide and dacarbazine (response rates 32- 49%), and additionally gemcitabine with a second agent such as docetaxel (response rate 18-53%, histology dependent), vinorelbine or dacarbazine (clinical benefit rate = 49%) (Brennan, 2013). In general, combination therapies improve response rates and PFS but have no impact on overall survival. Pazopanib is a multi-kinase inhibitor approved for second line or greater treatment of metastatic STS. In a double-blinded phase 3 trial pazopanib was compared to placebo in 369 patients with STS, excluding adipocytic sarcomas and gastrointestinal stromal tumor (GIST). Although the response rate was quite low at 6% there was a significant improvement in median PFS (4.6 versus 1.6 months). There was no difference in OS (van der Graaf *et al.*, 2012).

Prior to the pazopanib approval in 2012, there were no therapies approved by the U.S. Food and Drug Administration (FDA) for sarcomas other than (GIST) since doxorubicin in the 1980's. It has been challenging to develop therapies in sarcoma largely due to a combination of small patient numbers given the rarity of each individual sub-type and the inherent biologic complexity of this heterogeneous group of tumors. Additionally, sarcomas tend to occur more frequently in a younger patient population, with tumors like synovial sarcoma, rhabdomyosarcoma and osteosarcoma having a disproportionate number of adolescent and young adult (AYA) patients, a particularly vulnerable oncology population. Novel therapies are urgently needed.

There have been several published pre-clinical investigations exploring the role of FGFR in sarcoma. Schwartz *et al* evaluated platelet-derived growth factor receptor (PDGFR) / vascular endothelial growth factor receptor (VEGFR) and FGFR in cell lines revealing the differential response of the various sarcoma sub-types. The group used nintedanib (a tyrosine kinase inhibitor targeting FGFR, PDGFR and VEGFR) against various sarcoma cell lines. The Ewing sarcoma cell lines had little response, yet the synovial, dedifferentiated liposarcoma and malignant peripheral nerve sheath tumor (MPNST) cell lines had fairly robust responses (Figure 1). The authors went on to examine nintedanib in a xenograft synovial model (Figure 2). There was tumor volume reduction after treatment when compared to imatinib and control (Patwardhan *et al*., 2018).

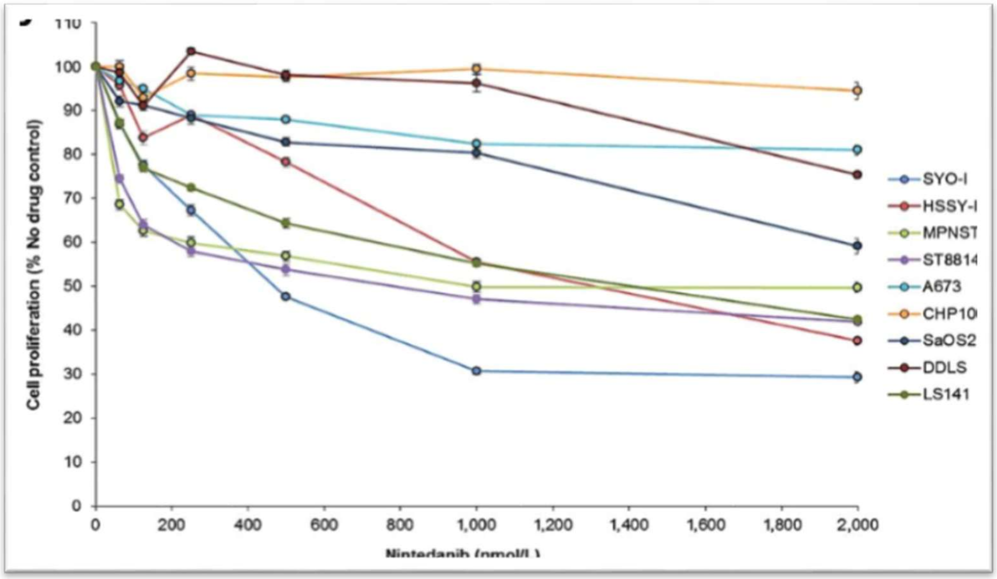

Figure 1: Effect of Nintedanib in Sarcoma Cell Lines

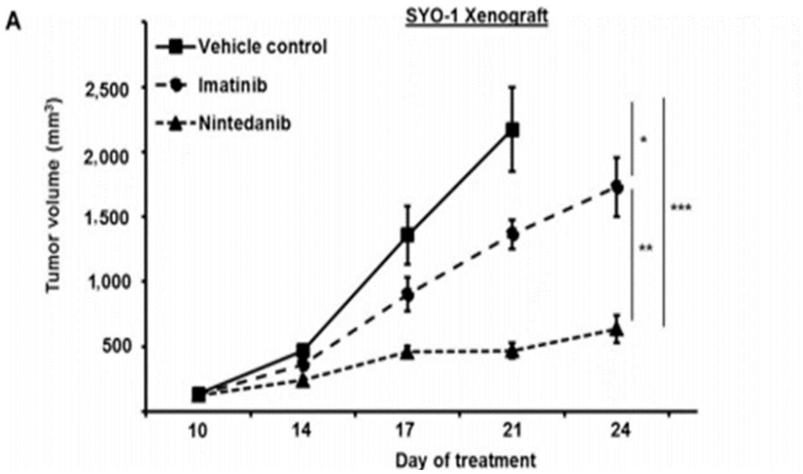

Figure 2: Effect of Nintedanib in Xenograft Synovial Model

Strauss, Lord, and colleagues evaluated the Astra-Zeneca FGFR inhibitor, AZD4547, focusing on osteosarcoma cell lines (Holme *et al*., 2018). In the figures below, Figure 3 is highlighting osteosarcoma cell lines (18 tumors) *vs* all other tumors (70 carcinomas) illustrating the relative sensitivity of osteosarcoma tumor cell lines. Figure 4 is looking specifically at FGFR1 amplification or gain *vs* no FGFR1 amplification or gain. Figure 5 is showing 2 different FGFR inhibitors against 2 cell lines, one with FGFR1 amplified and the other FGFR1 copy number neutral (Holme *et al*., 2018).

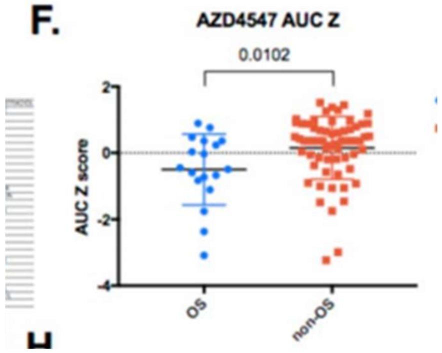

Figure 3: Relative Sensitivity of Osteosarcoma Tumor Cell Lines to AZD4547

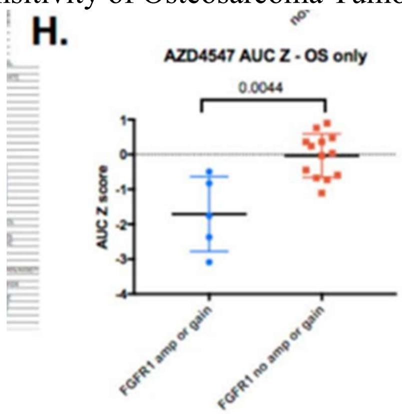

Figure 4: FGFR1 amplification or gain *vs* no FGFR1 amplification or gain

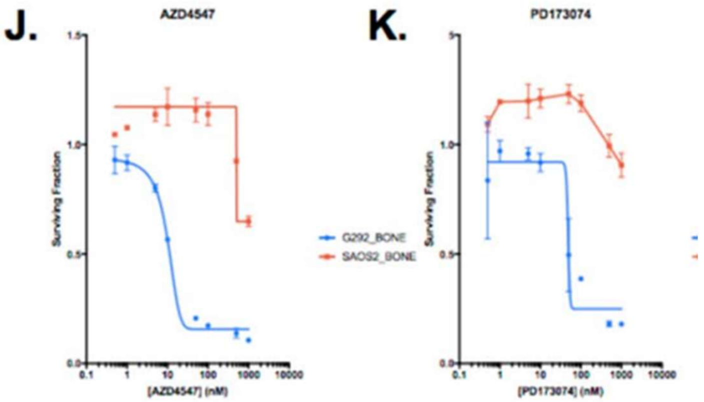

Figure 5: Effect of 2 different FGFR inhibitors against 2 cell lines, one with FGFR1 amplified and the other FGFR1 copy number neutral

Myklebost and colleagues evaluated a slightly different aspect of FGFR signaling, fibroblast growth factor receptor substrate 2 (FRS2) which is downstream of FGFR (Hanes *et al*, 2016). They had 2 dedifferentiated liposarcoma tumor samples from the same patient, one from the ileum and the other from the diaphragm. There was high level amplification of FRS2. They then exposed the cell cultures to the FGFR inhibitor BGJ398. Interestingly, the drug mimicked what was seen with the MDM2 inhibitor (MDM2 is amplified in dedifferentiated liposarcoma).

On the other hand, imatinib was ineffective and palbociclib, only slightly better, which mimics what is typically encountered in the clinical setting. They attributed their findings of reduced proliferation to a reversible cell cycle arrest in the G1 and G0 phases, as the liposarcoma cells resume proliferation after withdrawal from treatment (Hanes *et al*, 2016).

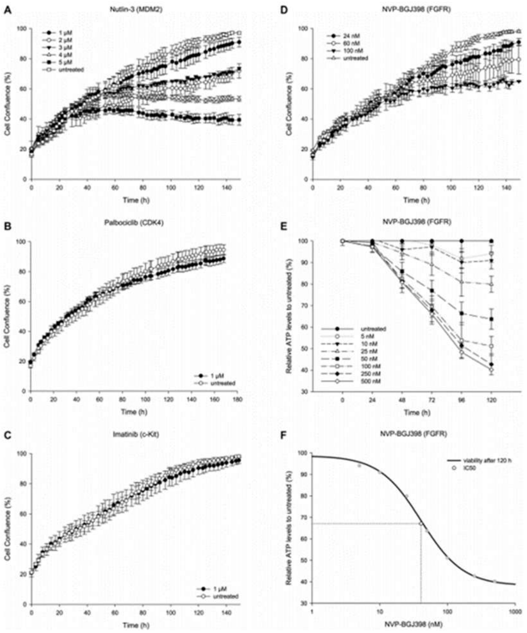

Figure 6: Dedifferentiated liposarcoma samples exposed to MDM2, CDK4/6, FGFR, and ckit inhibitors

Schafer and colleagues evaluated alveolar rhabdomyosarcoma cell lines suggesting that FGFR4 is responsible for the FGF-induced signaling (Crose *et al*., 2012). Cells treated with FGFR inhibitor BGJ398, inhibited FGFR4 auto-phosphorylation in rhabdomyosarcoma cells (Wachtel *et al*., 2014). Corinne Linardic's group from Duke evaluated FGFR4 blockade specifically, focusing on the differential between the two distinct subtypes, embryonal and alveolar rhabdomyosarcoma. They found greater FGFR4 expression in alveolar tissue than in embryonal. However, pharmacologic inhibition is seen in both subtypes in vitro and in vivo. In the figures below, RD is an embryonal cell line and Rh28 is an alveolar cell line. Their group went on to conclude that the consequences of FGFR4 blockade were present but distinctly different in the different subtypes, with apoptosis in the aRMS samples, while eRMS was merely an inhibition of proliferation (Crose *et al*., 2012).

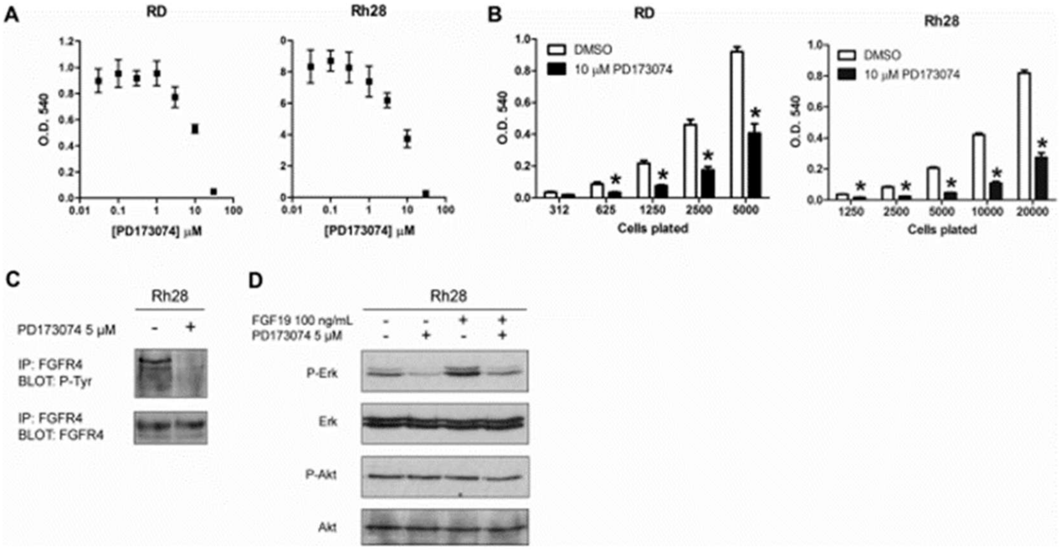

Figure 7: FGFR expression and blockade in alveolar and embryonal rhabdomyosarcoma

Further elaborating on the activity of FGFR inhibitors in rhabdomyosarcoma, Javed Khan's group focused on correlating high FGFR4 expression with advanced stage, aRMS histology and poor survival. They went on to make an inducible anti-FGFR4 shRNA cell line model and showed FGFR4 suppression leads to inhibition of in vivo growth and lung metastases (Taylor *et al.*, 2009).

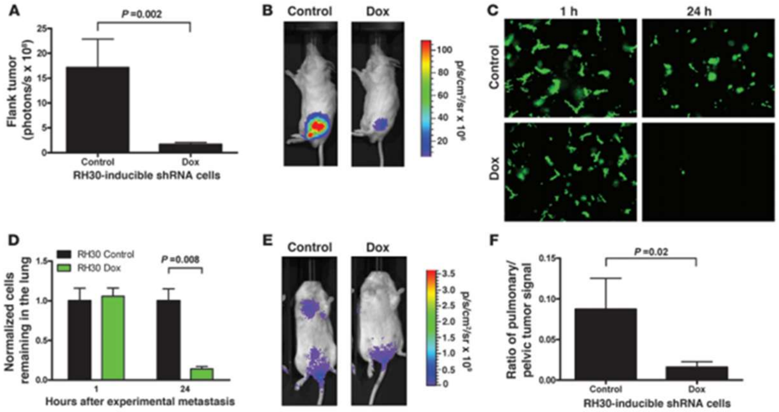

Figure 8: FGFR4 suppression leads to inhibition of *in vivo* growth and lung metastasis.

The Bernstein lab at Massachusetts General Hospital (MGH) and the Broad Institute and colleagues identified FGFR expression and a unique upregulation of FGF ligands in succinate dehydrogenase-deficient GIST (SDH-deficient GIST) (Flavahan *et al.*, 2019). While the majority of GIST subtypes bear activating mutations in the receptor tyrosine kinases *KIT* or *PDGFRA*, approximately 10-15% of adult GIST and the majority of pediatric GIST is driven by the loss of an SDH complex member (Pappo *et al.*, 2008; Boikos *et al.*, 2016). Unlike kinase mutant GIST, SDH-deficient GIST does not respond to treatment with imatinib, though sunitinib has shown anti-tumor activity in this disease (Janeway *et al.*, 2009; Janeway *et al.*, 2011). The mechanism

of the anti-neoplastic activity of sunitinib is unclear, and no other standard treatments are available for this disease.

More recently, in unpublished efforts to characterize PDX models of GIST, researchers at Dana Farber Cancer Institute (DFCI) performed RNA-seq on GIST surgical samples and PDX models of *KIT* mutant or SDH-deficient GIST tumors. Unsupervised hierarchical clustering of RNA-seq data demonstrate co-clustering of PDX with the tumor it was derived from, with separate clustering of all kinase mutant and SDH-deficient GIST (Figure 9A). In contrast to kinase mutant GIST, SDH-deficient GIST demonstrates reduced expression of SDH complex members in addition to a primary loss-of-function mutation (Figure 9B). Kinase mutant and SDH-deficient GIST express similar levels of KIT (Figure 9C), though PDGFRA levels are significantly reduced in SDH-deficient GIST (Figure 9D).

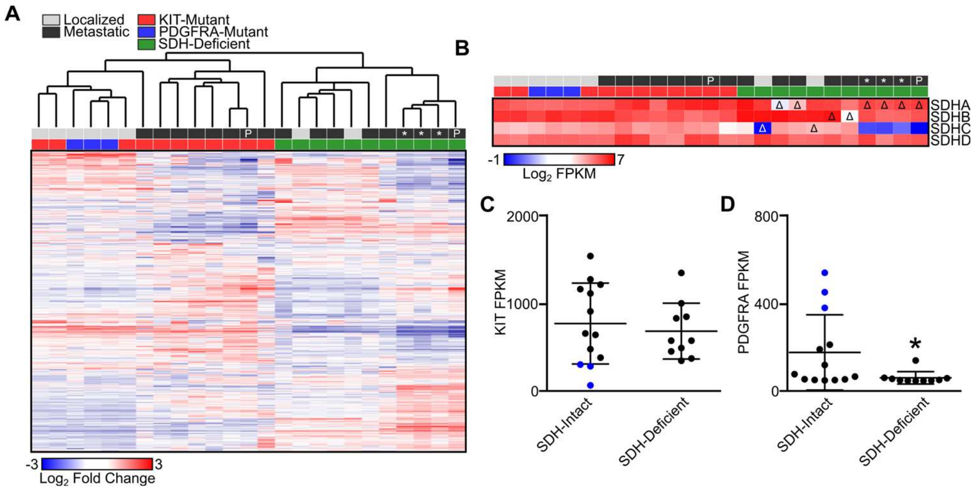

Figure 9: Characterization of GIST and PDX models by RNA-seq. (A) Heat map of row mean normalized log2 FPKM showing hierarchical clustering of all expressed genes (n=10,000) in SDH-intact (n=13) and SDH-deficient (n=10) GIST tumors and PDX derived from SDH-intact or SDH-deficient tumors ("P", n=2). The primary GIST gene mutation and presence of either localized or metastatic disease are indicated by color-coding. Samples derived from the same patient with SDH-deficient GIST at different tumor resection surgeries are indicated with an asterisk. (B) Log2 FPKM heat map of SDH complex member gene expression. The gene with the primary loss of function mutation in each sample is indicated with the symbol Δ. (C-D) FPKM of KIT and PDGFRA. Samples colored in blue bear a primary *PDGFRA* mutation.

To determine genes differentially expressed between kinase mutant (SDH-intact) and SDH-deficient GIST, we performed differential expression analysis of RNA-seq data, which identified significant upregulation of FGF3 and FGF4 in SDH-deficient GIST (Figure 10A-C). Expression of the FGFR family was similar across all GIST subtypes (Figure 10D), with the SDH-deficient PDX faithfully expressing FGF receptor and ligand in a similar fashion as surgical resection samples.

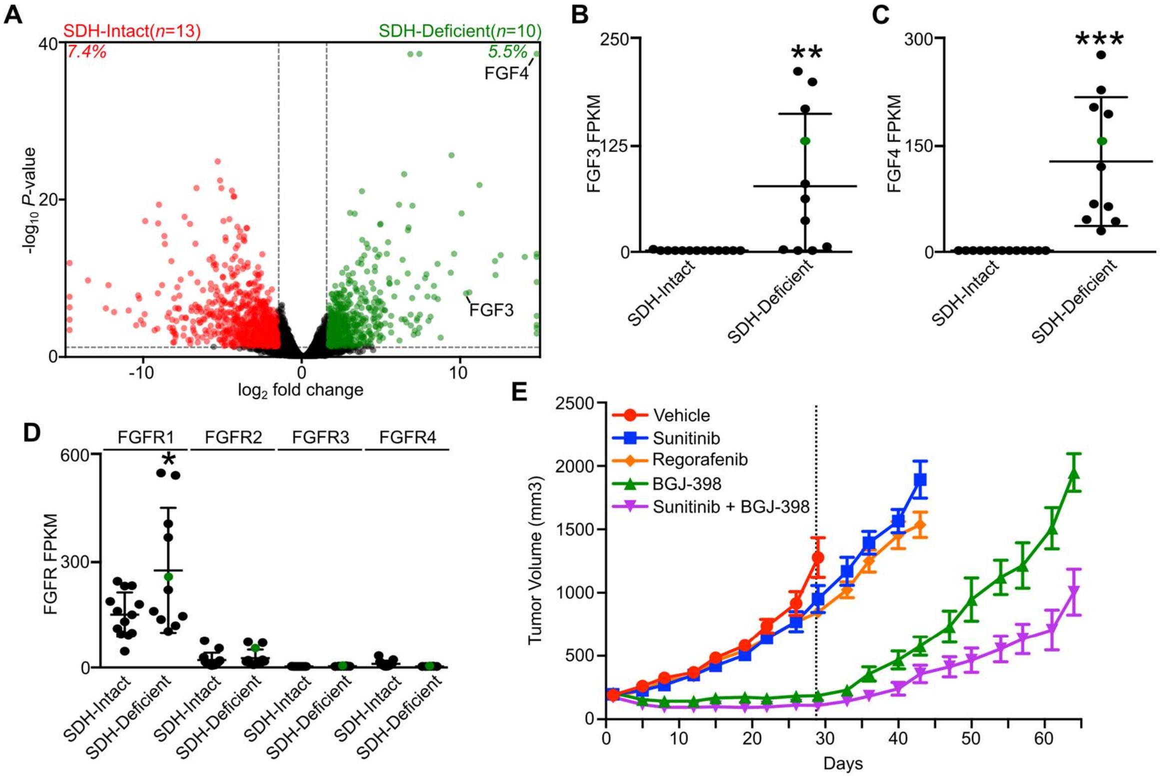

F

Group	P-value (vs vehicle)	P-value (vs sunitinib)	P-value (vs BGJ-398)
Sunitinib	0.0002	-	-
Regorafenib	<0.0001	ns	-
BGJ-398	<0.0001	<0.0001	-
BGJ-398 + Sunitinib	<0.0001	<0.0001	0.0323

Figure 10: FGF and FGFR expression in SDH-deficient GIST and susceptibility to FGFR inhibition. (A) Volcano plot of RNA-seq data in all expressed genes (n=~10,000) in SDH-intact or SDH-deficient GIST. The percent of genes unique to each subtype is indicated in the upper corners. FGF genes are highlighted. (B-D) FPKM or FGF3, FGF4 and FGFR1-4 in GIST subtypes. FPKM values for each gene in SDH-deficient GIST PDX are indicated in green circles. Tumors were compared by unpaired t-test; *, P<0.05; **, P<0.01; ***, P<0.001. (E) Effect of tyrosine kinase or FGFR inhibition in an SDH-deficient GIST PDX. Mice were treated for 28 days with indicated drug(s), n=8 per group. (F) Statistical comparisons of treatment groups by two-way ANOVA.

As no cell line model of SDH-deficient GIST has been developed, we have tested the role of FGFR signaling in SDH-deficient PDX using the FGFR inhibitor BGJ-398. Compared to sunitinib and regorafenib, BGJ-398 significantly reduced tumor volume of the SDH-deficient PDX (Figure 10E-F). Combination treatment with BGJ-398 and sunitinib was more effective than either drug alone. Taken together, these data argue for the relevance of FGFR signaling in SDH-deficient GIST. Though KIT-mutant GIST also expresses high levels of FGFR proteins (Li *et al*., 2015), the clinical relevance of FGFR signaling in disease pathogenesis for the kinase-mutant subtype of GIST is uncertain (Kelly *et al.*, 2019). In contrast, SDH-deficient GIST express similarly high levels of FGFR proteins, and their unique upregulation of FGF ligands suggests and auto-regulatory loop which may support the growth of these poorly understood cancers. These data suggest that inhibition of FGFR, alone and in combination with sunitinib,

may represent a novel treatment strategy for SDH-deficient GIST (Flavahan *et al.*, 2019).

2.2 CTEP IND Agent

2.2.1 Rogaratinib (BAY 1163877) (NSC 804782)

Rogaratinib (BAY 1163877) is a novel small molecule kinase inhibitor which selectively inhibits FGFRs 1, -2, -3, and -4 (Rogaratinib Investigator's Brochure, 2019). Rogaratinib (BAY 1163877) exhibits strong *in vivo* anti-tumor efficacy with good tolerability in monotherapy in animal tumor models.

2.2.1.1 Mechanism of Action

Dysregulation of FGFR expression and activity is considered oncogenic and affects all four FGFR subtypes and various human cancers, including sarcoma, multiple myeloma, breast, bladder, prostate, gastric, or endometrium cancer (Rogaratinib Investigator's Brochure, 2019). Rogaratinib (BAY 1163877) is a novel small molecule kinase inhibitor which selectively inhibits FGFR1, 2, 3, and 4 and showed efficacy in monotherapy in various preclinical cancer models. Additive to synergistic effects with broad range kinase inhibitors, targeted agents or conventional chemotherapeutics was also demonstrated. Rogaratinib (BAY 1163877) showed superior activity in models with overexpression of FGFR1, 2, or 3 mRNA or in models with pathway activation due to mutations in FGFR3. These results indicate that the clinical development of rogaratinib (BAY 1163877) could result in an improved and more effective alternative compared to current treatment and patient stratification options. Clinical safety and efficacy are currently being explored in four ongoing studies (2 monotherapy studies and 2 combination studies) in patients with advanced cancer.

2.2.1.2 Nonclinical Summary

Rogaratinib (BAY 1163877) exhibits monotherapy and combination anti-tumor efficacy in syngeneic and human tumor models characterized by addiction to the FGFR pathway either by gene alteration and/or messenger ribonucleic acid (mRNA) overexpression of FGFR1, -2, -3, -4 and fibroblast growth factor 19 (FGF19) (Rogaratinib Investigator's Brochure, 2019). Monotherapy with rogaratinib (BAY 1163877) resulted in potent anti-tumor efficacy in C51 colon cancer syngeneic tumors. Rogaratinib (BAY 1163877) inhibits the proliferation of lung cancer cell lines National Cancer Institute (NCI)-H1581, DMS114 as well as bladder cancer cell lines RT112 and JMSU-1. Moreover, rogaratinib (BAY 1163877) specifically inhibits phosphorylation of downstream extracellular signal-regulated kinases (ERK). Regarding secondary pharmacodynamics, rogaratinib (BAY 1163877) moderately binds to imidazoline (I2), 5-hydroxytryptamine (serotonin) receptor 2B (serotonin 5-HT2B) and 5-hydroxytryptamine receptor 4 (5-HT4), phosphodiesterase isozyme-5 (PDE5), adrenergic alpha2B, and adenosine transporter in vitro at a concentration of 10 µM. Safety pharmacology parameters of vital organ functions (central nervous, cardiovascular [including. electrocardiogram {ECG}], and respiratory system) in rats and dogs showed no effects of rogaratinib (BAY 1163877) except for a moderate reduction of locomotor activity and muscle tone and a slight decrease in respiratory function. Rogaratinib (BAY 1163877) does not have the potential for QT/QTc (QT interval in ECG/ QT

interval corrected for heart rate) prolongation in vivo, although *in vitro* human ether-a-go-go-related gene potassium channel (hERG K+) current was moderately blocked and repolarization was delayed in terms of action potential duration at 50% and 90% of repolarization (APD50/APD90) prolongation in rabbit Purkinje fibers.

2.2.1.3 Summary of Clinical Experience

As of October 10, 2019, approximately 306 patients with advanced cancer have been treated with rogaratinib (BAY 1163877) in four Phase I, one Phase Ib/II and one Phase II/III trials (Rogaratinib Investigator's Brochure, 2019). Out of these 306 patients, approximately 264 patients had received rogaratinib (BAY 1163877) monotherapy, 16 patients had received rogaratinib (BAY 1163877) in combination with copanlisib (NCT03517956) and 26 patients had received rogaratinib (BAY 1163877) in combination with atezolizumab (NCT03473756).

2.2.1.4 Clinical Pharmacokinetics and Pharmacodynamics

Pharmacokinetic results for rogaratinib (BAY 1163877) are available from the ongoing single-agent dose-escalation clinical trial (NCT01976741) in patients with advanced solid tumors (Rogaratinib Investigator's Brochure, 2019). After oral administration, rogaratinib (BAY 1163877) was rapidly absorbed with median time to reach maximum drug concentration in plasma (t_{max}) typically observed around 2 hours. Plasma concentrations declined with a geometric mean half-life of approximately 12 hours following a single dose of 800 mg. A more than 30% increase in area under the curve (AUC) from time 0 to time 12 hour (AUC_{0-12}) was observed after multiple dosing (twice daily [BID] administration). Relative bioavailability assessment after 100 mg single dose indicated similar exposures were achieved using the liquid service formulation (LSF) and tablet formulations. After single dose, rogaratinib (BAY 1163877) exhibited dose-proportional increase in exposure in the dose range 50 mg to 200 mg and less than dose-proportional increase at higher dose levels with similar observation after multiple dosing. Less than dose-proportional increase may be attributed to saturable absorption. Administration of 800 mg dose of rogaratinib (BAY 1163877) following consumption of high-fat high-calorie meal did not affect its pharmacokinetic (PK). Therefore, rogaratinib (BAY 1163877) may be administered with or without food. After administration of single oral dose of 800 mg, less than 1% of the administered drug was eliminated unchanged in cumulative urine collected over 24 h post-dose indicating renal elimination of rogaratinib (BAY 1163877) is a minor elimination pathway. PK data in Japanese patients enrolled in Study NCT02592785 and other Asian (South Korean and Singaporean) patients enrolled in Study NCT01976741compared to non-Asian patients enrolled in Study NCT01976741do not suggest clinically relevant differences in rogaratinib (BAY 1163877) exposure.

NCT01976741

As of October 10, 2019, 168 patients with advanced cancer were treated with rogaratinib (BAY 1163877) in Study NCT01976741: 23 patients in the dose-escalation cohorts and 145 patients in the expansion cohorts (Rogaratinib Investigator's Brochure, 2019). Out of those 168 patients, 159 patients (21 patients in the dose-escalation cohort and 138 patients in expansion cohorts) were evaluable for efficacy using Response Evaluation Criteria in Solid Tumors (RECIST) 1.1

criteria. One patient achieved complete response (CR) (urinary bladder cancer [UBC] cohort), 18 patients had partial response (PR), 80 patients had stable disease (SD), and 51 patients had progressive disease (PD).

NCT02592785

In the completed Study 16958, 9 patients with advanced cancer were treated with rogaratinib (BAY 1163877) administered orally BID at 600 and 800 mg (Rogaratinib Investigator's Brochure, 2019). All patients were evaluable for efficacy using RECIST 1.1 criteria. None of the patients achieved CR (UBC cohort), one patient (11.1%) achieved PR (epipharynx cancer, at 800 mg BID), five patients (55.6%) had SD and three patients (33.3%) had PD as their best overall response.

Maximum Tolerated Dose (MTD)/Recommended Dose

Per the data monitoring committee (DMC) evaluation of NCT03410693 study full data analysis and review on August 23, 2019, the safety of rogaratinib (BAY 1163877) is consistent to the known safety profile of the compound (Rogaratinib Investigator's Brochure, 2019). 800 mg BID could be considered as dose to use for monotherapy studies in the future.

2.2.1.5 Clinical Safety Summary

NCT01976741

The most common treatment-emergent adverse events (TEAEs) of any grade which were assessed as related to the study drug occurring in ≥10% of the patients were: blood phosphorus increased (57.7%), diarrhea (41.7%), fatigue (20.8%), decreased appetite (19.6%), alopecia (19.0%), dry mouth (17.9%), nausea (15.5%), dysgeusia (14.9%), dry skin (12.5%), and arthralgia and stomatitis (10.1% each). The most common TEAEs which were assessed as related to the study drug and were classified as Grade 3 or Grade 4 or Grade 5 event occurring in ≥3% of the patients were: lipase increased and fatigue (4.8% each).

NCT02592785

The most common TEAEs of any grade assessed as related to the study drug occurring in ≥20% of the patients were: hyperphosphataemia (88.9%), diarrhoea, dysgeusia, stomatitis and constipation (33.3% each), alanine aminotransferase increased, aspartate aminotransferase increased, gamma glutamyltransferase increased, dry skin, keratitis, blood bilirubin increased and blood alkaline phosphatase increased (22.2% each). The TEAEs which were assessed to be causally related to the study drug and classified as Grade 3 events were: hyponatraemia and alanine aminotransferase increased (11.1% each). There were no Grade 4 or Grade 5 TEAEs assessed as related to the study drug.

Adverse events of special interest of rogaratinib (BAY 1163877)

In Study NCT01976741, 20 (11.9%) patients experienced adverse events of special interest

(AESI) of retinal disorders assessed as causally related to rogaratinib (BAY 1163877).

2.3 Rationale

FGFR has recently been explored as a potential target in multiple solid tumor sub-types. As part of a CTEP rogaratinib (BAY 1163877) project team presentation, Ellinghaus & Bender presented data that showed 40% of sarcomas from pre-screening data had FGFR mRNA overexpression as determined by RNAscope. In searching our own institutional molecular profiling data, we identified 1533 sarcomas, with 72 patients having FGFR mutations affecting coding regions or splice sites, and 206 patients with copy number changes. This prompted a more extensive systematic literature search to identify which sarcoma sub-types seem to harbor higher FGFR expression, and thus would be potential viable targets for FGFR inhibiting drugs like rogaratinib (BAY 1163877). Sarcomas that repeatedly demonstrated FGFR over-expression and showed pre-clinical evidence of response to various available FGFR inhibitors included: synovial sarcoma, dedifferentiated liposarcoma, MPNST, SDH- deficient GIST, osteosarcoma and rhabdomyosarcoma. For these reasons, we propose a multiple cohort, single arm, Simon two stage phase II study to evaluate rogaratinib (BAY 1163877) in sarcomas with FGFR alterations as identified on mutational testing (DNA or RNA).

Of note, we have significant preclinical data on the effects of FGFR inhibition on a PDX model of SDH-deficient GIST.

2.3.1 Effects of Rogaratinib (BAY 1163877) on Preclinical Models of SDH-deficient GIST

To determine the comparative effects of various inhibitors of receptor tyrosine kinases on an SDH-deficient PDX, we treated mice for 28 days with sunitinib, regorafenib, BGJ398 and/or rogaratinib (BAY 1163877). Rogaratinib (BAY 1163877) arms included two dosing levels, and an additional arm of rogaratinib (BAY 1163877) in combination with sunitinib. Over the 28-day period, neither sunitinib nor regorafenib had significant anti-tumor activity. In contrast, treatment with FGFR inhibitors BGJ398 or rogaratinib (BAY 1163877) significantly limited tumor growth (Figure 11), with the high dose of rogaratinib (BAY 1163877) (50 mg/kg) significantly reducing tumor volume compared to the low dose of rogaratinib (BAY 1163877) (25 mg/kg) or BGJ398. Following the dosing period, mice continued to be observed and tumors were able to grow without ongoing FGFR inhibition. These data suggest that FGFR signaling is essential for SDH-deficient GIST growth, and continuous inhibitor treatment is necessary for disease control.

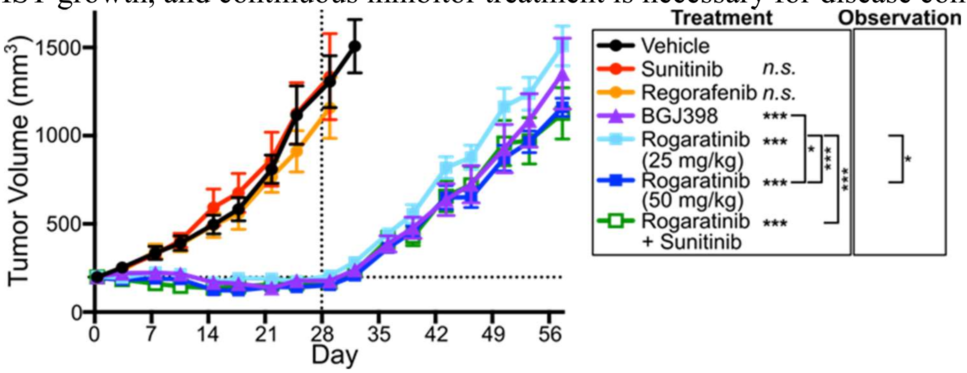

Figure 11: Effects of drug treatment on SDH-deficient GIST PDX tumor volume. Mice were treated for 28 days with vehicle (n=8), sunitinib 40 mg/kg QD (n=8), regorafenib 30 mg/kg QD

(n=8), BGJ398 20 mg/kg QD (n=8), rogaratinib (BAY 1163877) 25 mg/kg BID (n=8), rogaratinib (BAY 1163877) 50 mg/kg BID (n=8), or combination rogaratinib (BAY 1163877) 25 mg/kg BID and sunitinib 40 mg/kg QD (n=8). Tumor volume was measured during the 28-day treatment period and during the subsequent period of observation, with statistical calculations for these two periods indicated. Tumor volume in mm3 is indicated as mean ± SEM. The vertical dotted line indicates the end of the treatment period, and horizontal line the average baseline tumor volume. Data were analyzed by two-way ANOVA, with comparison to the vehicle condition unless otherwise indicated; *, P<0.05; **, P<0.01; ***, P<0.001. All single agent treatment groups were well tolerated. In the combination group, 4 of 8 mice required drug holiday between 1 and 11 days due to >15% body weight loss.

2.3.2 Pharmacodynamic Effects of Rogaratinib (BAY 1163877) Treatment on Preclinical Models of SDH-deficient GIST

To determine the mechanisms of toxicity of rogaratinib (BAY 1163877) treatment in SDH-deficient GIST, we treated tumor-bearing mice for five days with vehicle control, sunitinib, rogaratinib (BAY 1163877) or the combination of sunitinib and rogaratinib (BAY 1163877) and harvested tumors 4 or 24 hours after the final treatment dose. Tumor lysates were analyzed by Western blot for relative abundance of KIT, phospho-KIT, FGFR1, phospho-FGFR1, phospho-ERK and total ERK (Figure 12). Compared to control conditions, all treatment conditions reduced KIT and phospho-KIT levels, while phospho-FGFR1 levels were only reduced with rogaratinib (BAY 1163877) or combination rogaratinib (BAY 1163877) and sunitinib treatment (Figure 12A-C). The levels of phospho-ERK, a downstream mediator of receptor tyrosine kinase signaling, was significantly reduced only with rogaratinib (BAY 1163877) or combination treatments at the 4-hour time point, with recovery of phospho-ERK levels by 24 hours after drug administration.

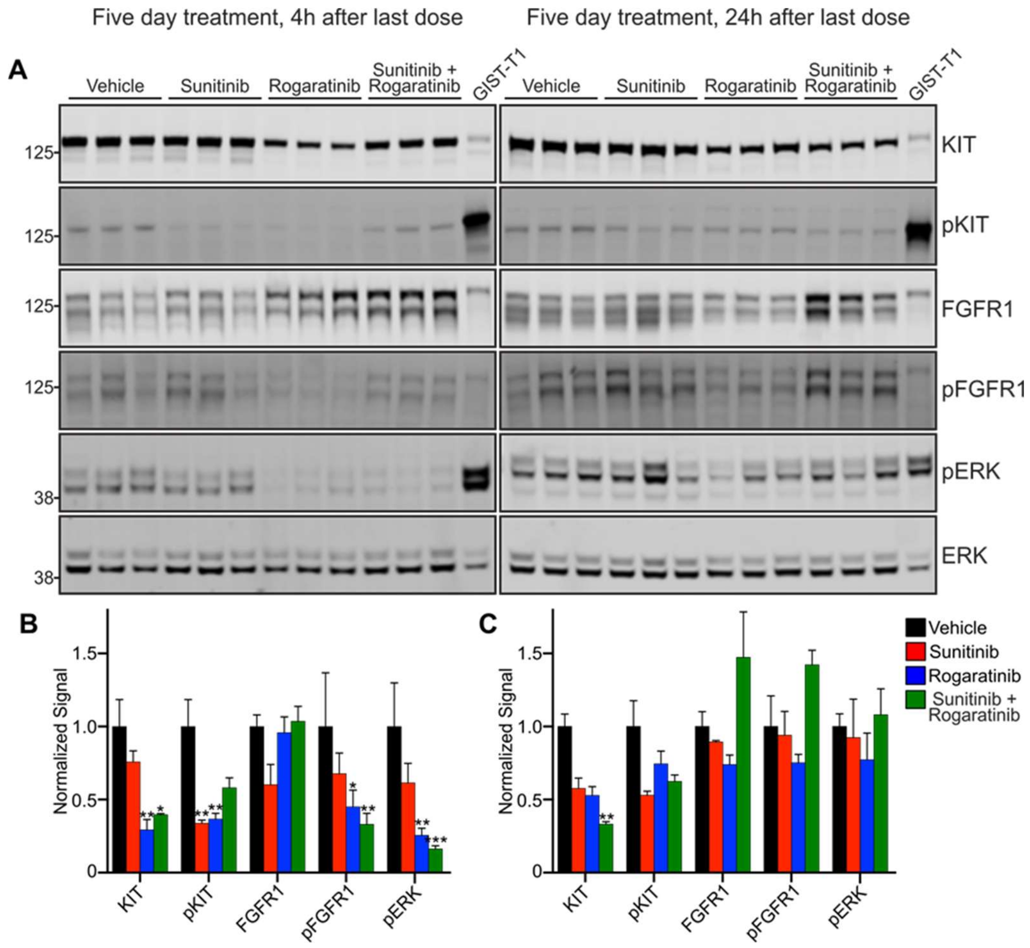

Figure 12: Effects of short-term drug treatment on SDH-deficient GIST PDX protein levels. A, Western blot for the indicated proteins following five days of drug treatment with vehicle (n=3), sunitinib 40 mg/kg QD (n=3), rogaratinib (BAY 1163877) 50 mg/kg BID (n=3), or combination rogaratinib (BAY 1163877) 25 mg/kg BID and sunitinib 40 mg/kg QD (n=3). Tumors were harvested after 4h (left column) or 24h (right column) of the final treatment administration. Quantification of Western blot protein intensity with signal normalized to total ERK as a loading control and to the vehicle condition at 4h (B) or 24h (C). Data were analyzed by one-way ANOVA, with comparison to the vehicle condition; *, $P<0.05$; **, $P<0.01$; ***, $P<0.001$.

To further explore mechanisms of rogaratinib (BAY 1163877) toxicity in SDH-deficient GIST, we performed RNA-seq on this five-day treatment cohort (Figure 13). Unsupervised hierarchical clustering of transcriptional data demonstrates co-clustering of all rogaratinib (BAY 1163877) and combination rogaratinib (BAY 1163877) and sunitinib samples, with separate co-clustering of sunitinib and vehicle treated mice (Figure 13A). Using gene set enrichment analysis (GSEA), rogaratinib (BAY 1163877) treated tumors showed significant decreases in transcripts related to cell cycle progression and MTOR signaling, both at 4 and 24 hours (Figure 13B-C). Levels of KIT gene expression trended towards lower transcript abundance in rogaratinib (BAY 1163877) treated tumors, while FGFR1 levels showed an increasing trend with rogaratinib (BAY 1163877) treatment (Figure 13D), as seen by Western blot (Figure 12). GPR20, another histologic marker of GIST, significantly decreased with rogaratinib (BAY 1163877) treatment (Figure 13D). Sprouty and DUSP family proteins antagonize receptor tyrosine kinase signaling, and their

expression is highly sensitive to changes in signal transduction. In rogaratinib (BAY 1163877) and combination treatment groups at 4 hours, SPRY2, SPRY4 and DUSP6 transcripts were significantly reduced with FGFR inhibition, and levels rebound following 24 hours of drug cessation (Figure 13E). Finally, while expression levels of ANO1 show a trend towards decreasing with rogaratinib (BAY 1163877) treatment, FGF3 and FGF4 transcripts are highly and significantly reduced with FGFR inhibition (Figure 13F). Taken together, these data suggest that signal transduction through FGFR is essential for growth of SDH-deficient GIST, and that FGFR inhibition with rogaratinib (BAY 1163877) reduces mitogenic signal transduction. Further, rogaratinib (BAY 1163877) silences expression of FGF3 and FGF4, which disrupts the autocrine loop driving FGFR signaling that supports this disease.

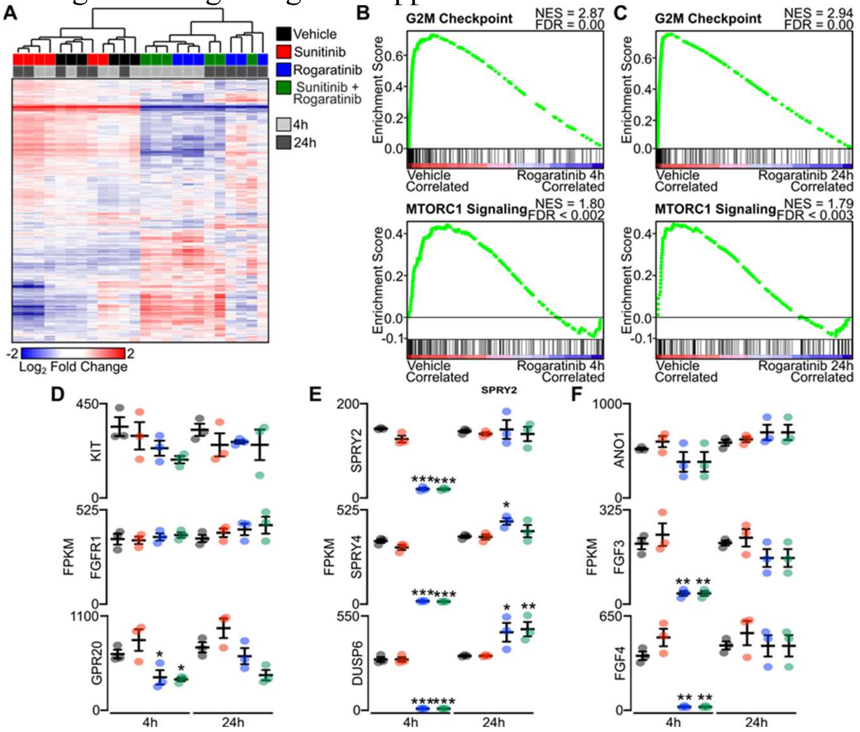

Figure 13: Effects of short-term drug treatment on PG20 transcription. A, Unsupervised hierarchical clustering of RNA-seq data following five days of drug treatment with vehicle (n=3), sunitinib 40 mg/kg QD (n=3), rogaratinib (BAY 1163877) 50 mg/kg BID (n=3), or combination rogaratinib (BAY 1163877) 25 mg/kg BID and sunitinib 40 mg/kg QD (n=3), with tumors harvested after 4h or 24h of the final treatment administration. B, GSEA of Hallmark data sets for G2M Checkpoint and MTORC1 Signaling comparing vehicle and rogaratinib (BAY 1163877) groups harvested 4h post-final treatment. C, GSEA of Hallmark data sets for G2M Checkpoint and MTORC1 Signaling comparing vehicle and rogaratinib (BAY 1163877) groups harvested 24h post-final treatment. D, Expression in FPKM of KIT, FGFR1 and GPR20 in vehicle (grayscale), sunitinib (red), rogaratinib (BAY 1163877) (blue) and combination rogaratinib (BAY 1163877) and sunitinib (green) tumors harvest 4h or 24h following the final treatment administration. E, Expression in FPKM of negative regulators of RTK signaling SPRY2, SPRY4 and DUSP6. F, Expression in FPKM of ANO1, FGF3 and FGF4. Data were analyzed by one-way ANOVA, with comparison to the vehicle condition; *, $P<0.05$; **, $P<0.01$; ***, $P<0.001$.

2.3.3 FGFR expression across sarcoma.

To evaluate levels of FGFR1-4 across sarcoma tumors, PDX and cell lines, we performed RNA-seq to compare transcript abundance from samples generated in our center (Figure 14). SDH-deficient GIST and KIT or PDGFRA mutant GIST expressed the highest levels of FGFR1 and FGFR2, though other histologies including leiomyosarcoma, dermatofibrosarcoma protuberans and others abundantly expressed FGFR1. By contrast, angiosarcoma expressed relatively higher levels of FGFR3, and rhabdomyosarcoma relatively higher levels of FGFR4. These preliminary results will support additional preclinical research on the role of FGFRs in supporting diverse sarcoma biology.

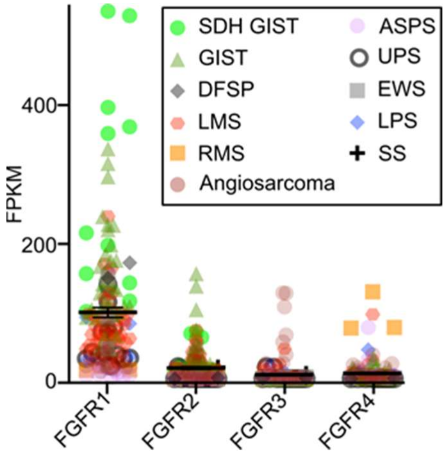

Figure 14: Expression of FGFRs across sarcoma tumors and models. Expression in FPKM of FGFR1-4 in sarcoma tumors, PDX and cell lines, including SDH-deficient GIST (n=12), KIT or PDGFRA mutant GIST (n=36), dermatofibrosarcoma protuberans (DFSP, n=2), leiomyosarcoma (LMS, n=49), rhabdomyosarcoma (RMS, n=3), angiosarcoma (n=21), alveolar soft part sarcoma (ASPS, n=8), undifferentiated pleomorphic sarcoma (UPS, n=13), Ewing sarcoma (EWS, n=3), liposarcoma (n=7) and synovial sarcoma (n=2).

2.4 Correlative Studies Background

2.4.1 Mutations and fusions in FGFR 1, 2, 3, and 4

Alterations in FGFR 1-4 have been associated with multiple subtypes of sarcoma (See Sections 2.1 and 2.3 for detailed discussion). We hypothesize that inhibiting these targetable, potential driver alterations, with rogaratinib (BAY 1163877) will disrupt tumor growth and lead to clinical benefit.

2.4.2 SDH

The Bernstein lab at Massachusetts General Hospital (MGH) and the Broad Institute and colleagues identified FGFR expression and a unique upregulation of FGF ligands in succinate dehydrogenase-deficient GIST (SDH-deficient GIST) (Flavahan *et al*, 2019) (See Sections 2.1 and 2.3 for detailed discussion). We hypothesized that FGFR inhibition with rogaratinib (BAY 1163877) will reduces mitogenic signal transduction and disrupt tumor growth in SDH-deficient GIST.

2.4.3 Whole Exome Sequencing (WES)

Whole exome sequencing (WES) will be performed to identify and characterize the spectrum of FGFR mutations and co-occurring alterations in patients with sarcoma enrolled onto this study. Characterization of FGFR alterations, and any potential downstream collaborating genomic aberrations, may better define the spectrum of FGFR alterations predictive of therapeutic response.

Our preclinical data in SDH-deficient GIST demonstrates expression of the ligand FGF3, FGF4 and receptor FGFR, autocrine loop in both archival material and in a PDX model (Figure 13F). WES will allow for characterization of specific SDH-alterations and thereby allow further exploratory analyses of genomic findings to outcomes in this population.

For the sarcoma cohort, baseline tumor biopsy will allow for WES prior to treatment initiation. This will allow for retrospective confirmation of the FGFR alteration which led to patient eligibility while concurrently allow for exploration of the genomic landscape of the enrolled population.

2.4.4 RNA Sequencing (RNAseq)

We have recently identified that SDH-deficient GIST is characterized by epigenetic dysregulation in the form of insulator dysfunction and altered chromosomal topology (Flavahan *et al.*, Nature 2019). In SDH-deficient GIST tumors and our PDX model of this disease, the ANO1 enhancer aberrantly drives high expression of FGF3 and FGF4, leading to autocrine activation of FGFR1. In preclinical studies shown in Section 2.3, we have identified a gene expression signature in SDH-deficient GIST that is altered by FGFR inhibition by rogaratinib (BAY 1163877), which includes the loss of negative regulators of receptor tyrosine kinase signaling and reduction in FGF3 and FGF4 expression. The pre-study biopsy will establish the expression of FGFRs, FGF3/4 and the signal transduction system we have characterized that entrains tumor growth. Biopsy and RNA-seq of the flash frozen tissue at disease progression will help establish biological mechanisms of drug resistance and whether alterations in the baseline gene expression program are present. Gene expression profiling of FGFR-altered sarcomas would also provide the opportunity to assess the expression of FGFRs and downstream mediators that may be similarly predictive of disease response to FGFR inhibition.

Our preclinical data in SDH-deficient GIST demonstrates expression of the ligand FGF3, FGF4 and receptor FGFR, autocrine loop in both archival material and in a PDX model (Figure 13F). For patients with SDH-deficient GIST, baseline tumor biopsies will allow us to assess the autocrine production of FGF3 and FGF4 leading to subsequent autocrine activation of FGFR1. This will be assessed by RNAseq and can be correlated in an exploratory manner with response to therapy.

For the sarcoma cohort, baseline tumor biopsy will allow for RNAseq prior to treatment initiation. This will allow for retrospective confirmation of the FGFR alteration which led to patient eligibility while concurrently allow for exploration of the genomic landscape of the

enrolled population.

2.4.5 Pharmacokinetics (PK)

These studies are necessary to characterize the variability in exposure to rogaratinib. After single or multiple doses, the variability in exposure ranges from ~35% in maximal concentrations and ~45% for total exposure (area under the curve) (Rogaratinib Investigator's Brochure, 2019). Trough samples will be collected when the patients are at clinic visits to correlate with pharmacodynamics effects (*i.e.*, clinical response, toxicity, and markers of FGFR pathway inhibition). Additional trough samples may be collected after a dose reduction to correlate the exposure at tolerable doses.

3. PATIENT SELECTION

3.1 Eligibility Criteria

3.1.1 Participant must have histologically confirmed sarcoma with FGFR alteration identified by next-generation sequencing profiling with the exception of SDH-deficient GIST who can be enrolled regardless of FGFR status. Initial testing can be performed on archival tissue, if available. Patients must have locally advanced or metastatic disease that is not amenable to surgery.

3.1.2 Presence of measurable disease: Patient must have measurable disease. See Section 12 (Measurement of Effect) for the evaluation of measurable disease.

3.1.3 Patients must have progressed following at least one standard prior chemotherapy regimen with the exception of SDH-deficient GIST for which there is no standard of care.

3.1.4 Participant must be willing to undergo pre-treatment biopsy if disease site is amenable to biopsy and low risk for the biopsy procedure. If biopsy is not possible, eligibility may be approved after discussion with the Study Chair. Of note, a minimum of 15 participants in each arm open to stage 2 should have disease amenable to biopsy. For those arms open in stage 1, all patients should have biopsiable disease.

3.1.5 Age ≥18 years.
Because no dosing or adverse event data are currently available on the use of rogaratinib (BAY 1163877) in patients <18 years of age, children are excluded from this study.

3.1.6 ECOG performance status ≤2 (Karnofsky ≥60%, see Appendix A).

3.1.7 Patients must have adequate organ and marrow function as defined below:

– Hemoglobin	≥ 8.0 g/dL
– absolute neutrophil count	≥1,000/mcL
– platelets	≥100,000/mcL
– total bilirubin	≤1.5 × institutional upper limit of normal (ULN)

– AST(SGOT)/ALT(SGPT) ≤3.0 × institutional ULN (unless liver metastases are present in which case it must be ≤ 5 × ULN)

– glomerular filtration rate (GFR) ≥60 mL/min/1.73 m^2 (using the CDK-EPI formula see Appendix B)

3.1.8 Human immunodeficiency virus (HIV)-infected patients on effective non-CYP3A4 interacting (see Section 3.2.3) anti-retroviral therapy with undetectable viral load within 6 months are eligible for this trial.

3.1.9 For patients with evidence of chronic hepatitis B virus (HBV) infection, the HBV viral load must be undetectable on suppressive therapy, if indicated.

3.1.10 Patients with a history of hepatitis C virus (HCV) infection must have been treated and cured. For patients with HCV infection who are currently on treatment, they are eligible if they have an undetectable HCV viral load.

3.1.11 Patients with **treated brain metastases** are eligible if follow-up brain imaging after central nervous system (CNS)-directed therapy shows no evidence of progression.

3.1.12 Patients must be disease-free of prior invasive malignancies for > 5 years with the exception of curatively-treated basal cell or squamous cell carcinoma of the skin or carcinoma *in situ* of the cervix.

NOTE: If there is a history of prior malignancy, patients must not be receiving other specific treatment for that cancer.

3.1.13 Patients should have completed prior treatment for their cancer: chemotherapy or radiotherapy must have been completed for greater than 2 weeks (6 weeks for nitrosoureas or mitomycin C) prior to entering the study.

3.1.14 Patients should have recovered from adverse events due to prior anti-cancer therapy (*i.e.*, have residual toxicities > Grade 1) with the exception of alopecia.

3.1.15 Patients with known history or current symptoms of cardiac disease, or history of treatment with cardiotoxic agents, should have a clinical risk assessment of cardiac function using the New York Heart Association Functional Classification. To be eligible for this trial, patients should be class 2B or better.

3.1.16 Patients must have a QTc interval length of below 450 msec.

3.1.17 Participant is willing to comply with the protocol for the duration of the study including undergoing treatment and scheduled visits and examinations including follow up.

3.1.18 Participant must be able to swallow and maintain pills.

3.1.19 Women of childbearing potential must have a negative urine or serum pregnancy test within 28 days of initial dose of rogaratinib (BAY 1163877), and again within 7 days prior to treatment on day 1. If screening occurs within 7 days of day 1, only one pregnancy test is required.

3.1.20 The effects of rogaratinib (BAY 1163877) on the developing human fetus are unknown. For this reason and because kinase inhibitor agents are known to be teratogenic, women of child-bearing potential and men must agree to use adequate contraception (hormonal or barrier method of birth control; abstinence) prior to study entry, for the duration of study participation, and 4 months after completion of rogaratinib (BAY 1163877). Should a woman become pregnant or suspect she is pregnant while she or her partner is participating in this study, she should inform her treating physician immediately. Men treated or enrolled on this protocol must also agree to use adequate contraception prior to the study, for the duration of study participation, and 4 months after completion of rogaratinib (BAY 1163877) administration.

3.1.21 Ability to understand and the willingness to sign a written informed consent document. Participants with impaired decision-making capacity (IDMC) who have a legally-authorized representative (LAR) and/or family member available will also be eligible.

3.2 Exclusion Criteria

3.2.1 Patients who are receiving any other investigational agents.

3.2.2 History of allergic reactions attributed to compounds of similar chemical or biologic composition to rogaratinib (BAY 1163877).

3.2.3 Concomitant administration with sensitive substrates/narrow therapeutic index drugs of CYP3A4, P-gp BCRP, MATE1, and MATE2K, and strong inhibitors and inducers of CYP3A4 should be avoided. Use caution with strong inhibitors and inducers of P-gp. Because the lists of these agents are constantly changing, it is important to regularly consult a frequently-updated medical reference. As part of the enrollment/informed consent procedures, the patient will be counseled on the risk of interactions with other agents, and what to do if new medications need to be prescribed or if the patient is considering a new over-the-counter medicine or herbal product. (See Appendix C Patient Drug Information Handout and Wallet Card).

3.2.4 Concomitant administration of medications that prolong QT/QTc interval is prohibited in accordance with the published FDA guidance "E14 Clinical Evaluation of QT/QTc Interval Prolongation and Proarrhythmic Potential for Non-Antiarrhythmic Drugs".

3.2.5 Patients with disturbed calcium and/or phosphate metabolism are excluded from this study.

3.2.6 Patients with uncontrolled intercurrent illness.

3.2.7 Patients with psychiatric illness/social situations that would limit compliance with study requirements.

3.2.8 Pregnant women are excluded from this study because rogaratinib (BAY 1163877) is kinase inhibitor agent with the potential for teratogenic or abortifacient effects. Because there is an unknown but potential risk for adverse events in nursing infants secondary to treatment of the mother with rogaratinib (BAY 1163877), breastfeeding should be discontinued if the mother is treated with rogaratinib (BAY 1163877).

3.3 Inclusion of Women and Minorities

NIH policy requires that women and members of minority groups and their subpopulations be included in all NIH-supported biomedical and behavioral research projects involving NIH-defined clinical research unless a clear and compelling rationale and justification establishes to the satisfaction of the funding Institute & Center (IC) Director that inclusion is inappropriate with respect to the health of the subjects or the purpose of the research. Exclusion under other circumstances must be designated by the Director, NIH, upon the recommendation of an IC Director based on a compelling rationale and justification. Cost is not an acceptable reason for exclusion except when the study would duplicate data from other sources. Women of childbearing potential should not be routinely excluded from participation in clinical research. Please see http://grants.nih.gov/grants/funding/phs398/phs398.pdf.

4. REGISTRATION PROCEDURES

4.1 Investigator and Research Associate Registration with CTEP

Food and Drug Administration (FDA) regulations and National Cancer Institute (NCI) policy require all individuals contributing to NCI-sponsored trials to register and to renew their registration annually. To register, all individuals must obtain a Cancer Therapy Evaluation Program (CTEP) Identity and Access Management (IAM) account at https://ctepcore.nci.nih.gov/iam. In addition, persons with a registration type of Investigator (IVR), Non-Physician Investigator (NPIVR), or Associate Plus (AP) must complete their annual registration using CTEP's web-based Registration and Credential Repository (RCR) at https://ctepcore.nci.nih.gov/rcr.

RCR utilizes five person registration types.

- IVR: MD, DO, or international equivalent,
- NPIVR: advanced practice providers (*e.g.*, NP or PA) or graduate level researchers (*e.g.*, PhD),
- AP: clinical site staff (*e.g.*, RN or CRA) with data entry access to CTSU applications such as the Roster Update Management System (RUMS), OPEN, Rave, acting as a primary site contact, or with consenting privileges,
- Associate (A): other clinical site staff involved in the conduct of NCI-sponsored trials, and

- Associate Basic (AB): individuals (*e.g.*, pharmaceutical company employees) with limited access to NCI-supported systems.

RCR requires the following registration documents:

Documentation Required	IVR	NPIVR	AP	A	AB
FDA Form 1572	✓	✓			
Financial Disclosure Form	✓	✓	✓		
NCI Biosketch (education, training, employment, license, and certification)	✓	✓	✓		
GCP training	✓	✓	✓		
Agent Shipment Form (if applicable)	✓				
CV (optional)	✓	✓	✓		

An active CTEP-IAM user account and appropriate RCR registration is required to access all CTEP and Cancer Trials Support Unit (CTSU) websites and applications. In addition, IVRs and NPIVRs must list all clinical practice sites and Institutional Review Boards (IRBs) covering their practice sites on the FDA Form 1572 in RCR to allow the following:

- Addition to a site roster,
- Assign the treating, credit, consenting, or drug shipment (IVR only) tasks in OPEN,
- Act as the site-protocol Principal Investigator (PI) on the IRB approval, and
- Assign the Clinical Investigator (CI) role on the Delegation of Tasks Log (DTL).

In addition, all investigators acting as the Site-Protocol PI (Investigator listed on the IRB approval), consenting/treating/drug shipment investigator in OPEN, or as the Clinical Investigator (CI) on the DTL must be rostered at the enrolling site with a participating organization.

Additional information is located on the CTEP website at https://ctep.cancer.gov/investigatorResources/default.htm. For questions, please contact the **RCR Help Desk.**

4.2 Site Registration

This study is supported by the NCI Cancer Trials Support Unit (CTSU).

IRB Approval

Sites participating with the NCI Central Institutional Review Board (NCI CIRB) must submit the Study Specific Worksheet for Local Context (SSW) to the CIRB using IRBManager to indicate their intent to open the study locally. The NCI CIRB's approval of the SSW is automatically communicated to the CTSU Regulatory Office, but sites are required to contact the CTSU

Regulatory Office to establish site preferences for applying NCI CIRB approvals across their Signatory Network. Site preferences can be set at the network or protocol level. Questions about establishing site preferences can be addressed to the CTSU Regulatory Office.

In addition, the Site-Protocol PI (*i.e.*, the investigator on the IRB/REB approval) must meet the following five criteria to complete processing of the IRB/REB approval record:

- Holds an Active CTEP status,
- Rostered at the site on the IRB/REB approval (*applies to US and Canadian sites only*) and on at least one participating roster,
- If using NCI CIRB, rostered on the NCI CIRB Signatory record,
- Includes the IRB number of the IRB providing approval in the Form FDA 1572 in the RCR profile, and
- Holds the appropriate CTEP registration type for the protocol.

Additional Requirements

Additional requirements to obtain an approved site registration status include:

- An active Federalwide Assurance (FWA) number,
- An active roster affiliation with the Lead Protocol Organization (LPO) or a Participating Organization (PO), and
- Compliance with all protocol-specific requirements (PSRs).

4.2.1 Downloading Regulatory Documents

Download the site registration forms from the protocol-specific page located on the CTSU members' website. Permission to view and download this protocol and its supporting documents is restricted based on person and site roster assignment. To participate, the institution and its associated investigators and staff must be associated with the LPO or a PO on the protocol. One way to search for a protocol is listed below.

- Log in to the CTSU members' website (https://www.ctsu.org) using your CTEP-IAM username and password,
- Click on *Protocols* in the upper left of the screen
  - Enter the protocol number in the search field at the top of the protocol tree, or
  - Click on the By Lead Organization folder to expand, then select LAO-MA036 / Dana-Farber - Harvard Cancer Center LAO, and protocol number 10411,
- Click on *Documents*, select *Site Registration*, and download and complete the forms provided. (Note: For sites under the CIRB, IRB data will load automatically to the CTSU.)

4.2.2 Protocol Specific Requirements For 10411 Site Registration

- Specimen Tracking System Training Requirement:
  - All data entry users (Clinical Research Associate role) at each participating site will need to complete the Theradex-led training.
  - Theradex will provide a certificate of completion, which will need to be submitted to

the CTSU through the Regulatory Submission Portal.

- The training is a one-time only requirement per individual. If an individual has previously completed the training for another ETCTN study, the training does not need to be completed again nor does the certificate of completion need to be resubmitted to the CTSU. However, new versions of the Specimen Tracking System may require new training.
- This training will need to be completed before the first patient enrollment at a given site.
- Please contact STS Support at Theradex for the training.

4.2.3 Submitting Regulatory Documents

Submit required forms and documents to the CTSU Regulatory Office using the Regulatory Submission Portal on the CTSU website.

To access the Regulatory Submission Portal, log on to the CTSU members' website, go to the Regulatory section, and select Regulatory Submission.

Institutions with patients waiting that are unable to use the Regulatory Submission Portal should alert the CTSU Regulatory Office immediately in order to receive further instruction and support.

Delegation of Tasks Log (DTL)

Each site must complete a protocol-specific DTL using the DTL application in the Delegation Log section on the CTSU members' website. The Clinical Investigator (CI) is required to review and electronically sign the DTL prior to the site receiving an Approved site registration status and enrolling patients to the study. To maintain an approved site registration status the CI must re-sign the DTL at least annually and when a new version of the DTL is released; and activate new task assignments requiring CI sign-off. Any individual at the enrolling site on a participating roster may initiate the site DTL. Once the DTL is submitted for CI approval, only the designated DTL Administrators or the CI may update the DTL. Instructions on completing the DTL are available in the Help Topics button in the DTL application and include a Master Task List, which describes DTL task assignments, CI signature, and CTEP registration requirements.

4.2.4 Checking Site Registration Status

Site's registration status may be verified on the CTSU website.

- Click on *Regulatory* at the top of the screen
- Click on *Site Registration, and*
- Enter the site's 5-character CTEP Institution Code and click on Go
  - Additional filters are available to sort by Protocol, Registration Status, Protocol Status, and/or IRB Type.

Note: The status shown only reflects institutional compliance with site registration requirements

as outlined within the protocol. It does not reflect compliance with protocol requirements for individuals participating on the protocol or the enrolling investigator's status with the NCI or their affiliated networks.

4.3 Patient Registration

4.3.1 OPEN / IWRS

The Oncology Patient Enrollment Network (OPEN) is a web-based registration system available on a 24/7 basis. OPEN is integrated with CTSU regulatory and roster data and with the LPOs registration/randomization systems or the Theradex Interactive Web Response System (IWRS) for retrieval of patient registration/randomization assignment. OPEN or IWRS will populate the patient enrollment data in NCI's clinical data management system, Medidata Rave.

Requirements for OPEN access:

- A valid CTEP-IAM account.
- To perform enrollments or request slot reservations: Must be on an LPO roster, ETCTN corresponding roster, or PO roster with the role of Registrar. Registrars must hold a minimum of an Associate Plus (AP) registration type.
- If a DTL is required for the study, the registrar must hold the OPEN Registrar task on the DTL for the site.
- Have an approved site registration for the protocol prior to patient enrollment.

To assign an Investigator (IVR) or Non-Physician Investigator (NPIVR) as the treating, crediting, consenting, drug shipment (IVR only), or receiving investigator for a patient transfer in OPEN, the IVR or NPIVR must list the IRB number used on the site's IRB approval on their Form FDA 1572 in RCR. If a DTL is required for the study, the IVR or NPIVR must be assigned the appropriate OPEN-related tasks on the DTL.

Prior to accessing OPEN, site staff should verify the following:

- Patient has met all eligibility criteria within the protocol stated timeframes, and
- All patients have signed an appropriate consent form and HIPAA authorization form (if applicable).

Note: The OPEN system will provide the site with a printable confirmation of registration and treatment information. IWRS system also sends an email confirmation of the registration. You may print this confirmation for your records.

Access OPEN at https://open.ctsu.org or from the OPEN link on the CTSU members' website. Further instructional information is in the OPEN section of the CTSU website. For any additional questions, contact the CTSU Help Desk.

Patient enrollment for this study will be facilitated using the Slot Reservation System in conjunction with the registration system in OPEN. Prior to discussing protocol entry with the

patient, all site staff must use the CTSU OPEN Slot Reservation System or the IWRS Slot Reservation System to ensure that a slot on the protocol is available to the patient. Once a slot reservation confirmation is obtained, site staff may then proceed to enroll the patient to this study.

4.3.2 Special Instructions for Patient Enrollment

The enrolling site will use the Slot Reservation System in IWRS to reserve a slot.

Once reserved, the site will submit the following documentation to Dana-Farber Cancer Institute:

- For COHORT A: Local NGS report documenting FGFR alteration must be submitted and reviewed/approved by the study team prior to registration

- For COHORT B: Pathology reports documenting SDH-deficient GIST must be submitted and reviewed/approved by the study team prior to registration

This Study will use the ETCTN Specimen Tracking System (STS).

- All biospecimens collected for this trial must be submitted using the ETCTN Specimen Tracking System (STS) unless otherwise noted.
- The system is accessed through Rave user roles: "Rave CRA" and "Rave CRA (Labadmin)" for data entry at the treating institutions and "Biorepository" for users receiving the specimens for processing and storage at reference labs and the Early-Phase and Experimental Clinical Trials Biospecimen Bank (EET Biobank, formerly known as the ETCTN Biorepository).
- Please refer to the Medidata Account Activation and Study Invitation Acceptance link on the CTSU website in the Data Management section under the Rave Home tab and then under Rave Resource Materials tab.
- **Important: Failure to complete required fields in STS may result in a delay in sample processing.** Any case reimbursements associated with sample submissions will not be credited if samples requiring STS submission are not logged into STS.

Detailed instructions on use of the STS can be found in Section 5.4.

4.3.3 OPEN/IWRS Questions?

Further instructional information on OPEN is provided on the OPEN link of the CTSU website at https://www.ctsu.org or at https://open.ctsu.org. For any additional questions contact the CTSU Help Desk.

Theradex has developed a Slot Reservations and Cohort Management User Guide, which is available on the Theradex website: http://www.theradex.com/clinicalTechnologies/?National-Cancer-Institute-NCI-11. This link to the Theradex website is also on the CTSU website OPEN tab. For questions about the use of IWRS for slot reservations, contact the Theradex Helpdesk.

4.4 General Guidelines

Following registration, patients should begin protocol treatment within 7 days. Issues that would cause treatment delays should be discussed with the Principal Investigator. If a patient does not receive protocol therapy following registration, the patient's registration on the study may be canceled. The Study Coordinator should be notified of cancellations as soon as possible.

5. BIOMARKER, CORRELATIVE, AND SPECIAL STUDIES

5.1 Summary Table for Specimen Collection

Time Point	Specimen	Send Specimens To:
**Archival**		
	• Formalin-fixed paraffin-embedded (FFPE) tumor tissue block (preferred)[1]<br><br>If a block is not available, then submit:<br>• 1 H&E stained slide (3-5 µm)<br>• 30-50 unstained, uncharged, air-dried slides (10 µm). If not feasible, then a minimum of 20 unstained air-dried uncharged slides (10 µm) should be submitted with a minimum tumor content of 30-40%[2].	EET Biobank
**Pre-treatment (prior to C1D1)**		
	• 2 tissue cores, flash frozen[3]<br>• 10 mL blood in EDTA (mandatory)	EET Biobank
	• 1 × 4 mL blood in EDTA (mandatory)	JHU SKCCC Analytical Pharmacology Core Lab for the ChOP-KC
**Clinic Visits (C1D15, C2D1, C2D15, and C3D1)[4,5]**		
Pre-dose	• 1 × 4 mL blood in EDTA (mandatory)	JHU SKCCC Analytical Pharmacology Core Lab for the ChOP-KC
**Clinic Visits after a dose reduction (any Cycle/Day)**		
Pre-dose	• 1 × 4 mL blood in EDTA (mandatory)	JHU SKCCC Analytical Pharmacology Core Lab for the ChOP-KC
**Progression**		
	• 2 tissue cores, flash frozen[3](optional)	EET Biobank
	• 1 × 4 mL blood in EDTA (optional)	JHU SKCCC Analytical Pharmacology Core Lab for the ChOP-KC
[1]For archival tissue (if available), **a copy of the corresponding anatomic pathology report must be sent with the tissue and uploaded to Rave**. If submitting slides, then slides must be		

processed in order, and numbered sequentially (*e.g.*, H&E stained slide is created first and labeled 1, unstained slides are then created and numbered 2 – 51).<br>[2] ***Submission of specimens with <30% tumor content may not provide sufficient material for analysis***<br>[3]For new biopsies, **the Tissue Biopsy Verification Form (Appendix D*),* a copy of the radiology and/or operative reports from the tissue removal procedure, *and* the diagnostic anatomic pathology report** must be sent with the tissue to the EET Biobank.<br>[4]Patients should be instructed to bring their morning dose of rogaratinib (BAY 1163877) with them to the clinic.<br>[5]The dates of the PK samples are when the physical exams are scheduled. If a physical exam is shifted due to a holiday or inclement weather, the PK should be collected on that day (and not the exact day listed above).

5.2 Summary Table(s) for Research Biopsies

**Biopsy #:** 1				
**Trial Time Point:** Pre-treatment				
**Biopsy Definition:** Research – No Clinical Impact (All cores from a single biopsy procedure impact research goals, but do not directly impact patient care or benefit the patient.)				
**Core Priority**	**Use in the Trial**	**Biomarker Name(s)**	**Tumor Content Required**	**Post-Biopsy Processing**
1	Integrated	WES	≥ 70%	Flash frozen
2	Integrated	RNAseq	≥ 70%	Flash frozen

**Biopsy #:** 2				
**Trial Time Point:** Progression				
**Biopsy Definition:** Research – No Clinical Impact (All cores from a single biopsy procedure impact research goals, but do not directly impact patient care or benefit the patient.)				
**Core Priority**	**Use in the Trial**	**Biomarker Name(s)**	**Tumor Content Required**	**Post-Biopsy Processing**
1	Integrated	WES	≥ 70%	Flash frozen
2	Integrated	RNAseq	≥ 70%	Flash frozen

5.3 Specimen Procurement Kits and Scheduling

5.3.1 Specimen Procurement Kits

Kits for the collection and shipment of frozen specimens to the EET Biobank can be ordered online via the Kit Management system: (https://kits.bpc-apps.nchri.org/).

Users at the clinical sites will need to set up an account in the Kit Management system and select a specific clinical trial protocol to request a kit. Please note that protocol may include more than one type of kit. Each user may order two kits per kit type per day (daily max = 6 kits). Kits are

shipped ground, so please allow 5-7 days for receipt. A complete list of kit contents for each kit type is located on the Kit Management system website.

Note: Kits or supplies are only provided for specimens shipped to the EET Biobank. Institutional supplies must be used for all other specimen collection and processing.

5.3.2 Scheduling of Specimen Collections

Please adhere to the following guidelines when scheduling procedures to collect tissue:

- Tissue submitted as FFPE (blocks or slides) can be collected on any day but must be shipped to the EET Biobank on Monday through Thursday.

- Tissue specimens submitted frozen can be collected on any day but must be stored frozen and shipped to the EET Biobank on Monday through Thursday. In the event that frozen specimens cannot be shipped immediately, they must be maintained in a -70°C to -80°C freezer.

- Fresh blood specimens may be collected and shipped Monday through Friday.

5.4 Specimen Tracking System Instructions

5.4.1 Specimen Tracking System Overview and Enrollment Instructions

For the ETCTN STS, the following information will be requested:

- Protocol Number
- Investigator Identification
    - Institution and affiliate name
    - Investigator's name
- Eligibility Verification: Patients must meet all the eligibility requirements listed in Section 3.
- Additional Requirements:
    - Patients must provide a signed and dated, written informed consent form.

Upon enrolling a patient, IWRS will communicate with OPEN, assigning two separate and unique identification numbers to the patient, a Universal patient ID (UPID) and a Treatment patient ID. The UPID is associated with the patient and used each and every time the patient engages with the portion of this or any other protocol that uses the ETCTN Specimen Tracking System. The UPID contains no information or link to the treatment protocol. IWRS will maintain an association between the UPID for ETCTN biobanking and molecular characterization and any treatment protocols the patient participates in, thereby allowing analysis of the molecular characterization results with the clinical data.

Immediately following enrollment, the institutional anatomical pathology report for the diagnosis under which the patient is being enrolled must be uploaded into Rave. The report must include the surgical pathology ID (SPID), collection date, block number, and the IWRS-assigned UPID and patient study ID for this trial. For newly acquired biopsies without a corresponding

radiology report, the radiology and operative report(s) must also be uploaded into Rave, when available. **<u>Important</u>: Remove any personally identifying information, including, but not limited to, the patient's name, date of birth, initials, medical record number, and patient contact information from the institutional pathology report prior to submission.**

Additionally, please note that the STS software creates pop-up windows when reports are generated, so you will need to enable pop-ups within your web browser while using the software.

For questions regarding the Specimen Tracking System, please contact STS Support.

The Shipping List report **<u>must</u>** be included with all sample submissions.

5.4.2 <u>Specimen Labeling</u>

5.4.2.1 Blood Specimen Labels

Include the following on blood specimens (including whole blood):

- Patient Study ID
- Universal Patient ID (UPID)
- Specimen ID (automatically generated by Rave)
- Time point
- Specimen type (*e.g.*, blood, serum)
- Collection date (to be added by hand)
- Collection time for PK samples (to be added by hand)

5.4.2.2 Tissue Specimen Labels

Include the following on all tissue specimens or containers (*e.g.*, formalin jar):

- Patient Study ID
- Universal Patient ID (UPID)
- Specimen ID (automatically generated by Rave)
- Time point
- Specimen type (*e.g.*, flash frozen, *etc.*)
- Tissue type (P for primary, M for metastatic or N for normal)
- Surgical pathology ID (SPID) number (when applicable)
- Collection date (to be added by hand)
- Slide section number (only if archival tissue is submitted as slides) (to be added by hand)

5.4.2.3 Example of Specimen Label Generated by STS

STS includes a label printing facility, accessed via the Print Label CRF in the All Specimens folder. A generated PDF is emailed to the user as a result of saving that form.

The following image is an example of a tissue specimen label printed on a label that is 0.5" high

and 1.28” wide.

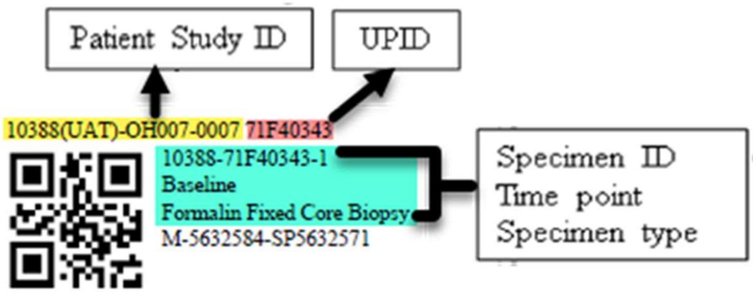

The QR code in the above example is for the Specimen ID shown on the second line.

Labels may be printed on a special purpose label printer, one label at a time, or on a standard laser printer, multiple labels per page. Theradex recommends the use of these low temperature waterproof labels for standard laser printers: https://www.labtag.com/shop/product/cryo-laser-labels-1-28-x-0-5-cl-23-colors-available/

The last line item on the label includes the following data points joined together:

1. Tissue only: Primary (P), Metastatic (M), Normal (N) tissue indicated at the beginning of the specimen ID; this field is blank if not relevant (*e.g*., for blood)
2. Block ID or blank if not relevant
3. SPID (Surgical Pathology ID) or blank if none
4. An optional alpha-numeric code that is protocol specific and is only included if the protocol requires an additional special code classification

Space is provided at the bottom of the label for the handwritten date and optional time. The last line on the example label is for the handwritten date and optional time.

5.4.3 <u>Overview of Process at Treating Site</u>

5.4.3.1 OPEN Registration

All registrations will be performed using the Oncology Patient Enrollment Network (OPEN) system. OPEN communicates automatically with the Interactive Web Response System (IWRS) which handles identifier assignments, any study randomization, and any prescribed slot assignments. If specimen analysis is required to determine eligibility, the protocol will be setup with multi-step registration.

Registration with eligibility specimen analysis:

1. Site enters first step data into OPEN.
2. IWRS receives data from OPEN, generates the Patient Study ID and the Universal Patient ID, both of which are sent back to OPEN.

3. IWRS sends first step registration data, including the IDs and a TAC of "NOT REG" directly to Rave.
4. The specimen tracking system in Rave is utilized for the specimen that contributes to eligibility determination.
5. Site enters second and any subsequent step data into OPEN including results of specimen analysis.
6. IWRS receives all data from OPEN, then sends it onto Rave with either the treatment TAC or a TAC of "SCRN FAIL".
7. In addition to the specimen tracking forms completed to determine eligibility, data entry for screen failure patients should include Histology and Disease, all forms in the Baseline folder, any lab forms connected to eligibility determination, and Off Treatment/Off Study.

Any data entry errors made during enrollment should be corrected in Rave.

5.4.3.2 Rave Specimen Tracking Process Steps

Step 0: Log into Rave via your CTEP-IAM account, then navigate to the appropriate participant.

Step 1: Complete the **Histology and Disease** form (but do not upload reports until a specimen label can be applied to them) and the Baseline forms regarding **Prior Therapies**. Enter the initial clinical specimen data:

- **Specimen Tracking Enrollment** CRF: Enter Time Point, Specimen Category, Specimen Type, Block number, Tissue type, Surgical Path ID, and number of labels needed (include extra labels to apply to reports to be uploaded). CRF generates unique Specimen ID.

Step 2: Print labels using report the Print Labels CRF located in the All Specimens folder, then collect specimen.

- Label specimen containers and write collection date on each label. After collection, store labeled specimens as described in Section 5.4.2.
- Apply an extra specimen label to *each* report before scanning. Return to the **Histology and Disease** form to upload any initial Pathology, Radiology, Molecular Reports (up to 4), Surgical (or Operative) reports. Return to **Specimen Tracking Enrollment** CRF to upload any molecular report (one per specimen) and/or specimen specific pathology or related report (one per specimen) and/or Tissue Biopsy Verification form (Appendix D). Uploaded reports should have protected health information (PHI) data, like name, date of birth, mailing address, medical record number or social security number (SSN), redacted. **Do not redact SPID, block number, diagnosis, or relevant dates (such as collection date), and include the UPID and patient study ID on each document** (either by adding a label or hand writing).

Step 3: Complete specimen data entry.

- **Specimen Transmittal** Form: Enter collection date and time and other required specimen details.

Step 4: When ready to ship, enter shipment information.

- **Shipping Status** CRF: Enter tracking number, your contact information, recipient, number of sample containers and ship date once for the first specimen in a shipment.
- **Copy Shipping** CRF: In the specimen folders for additional specimens (if any) that will be shipped with the initial specimen, please use the **Copy Shipping** form to derive common data into additional **Shipping Status** forms. A few unique fields will still need to be entered in **Shipping Status.**

Step 5: Print shipping list report and prepare to ship.

- Shipping List report is available at the site level.
- Print two copies of the shipping list, one to provide in the box, the other for your own records.
- Print pathology or other required reports to include in the box. Be sure the printed copy includes the specimen label.

Step 6: Send email notification.

- For only one of the specimens in the shipment, click "Send Email Alert" checkbox on the **Shipping Status** CRF to email recipient.

Step 7: Ship the specimen(s).

Step 8: Monitor the Receiving Status form located in each specimen folder for acknowledgment of receipt and adequacy.

5.5 Specimen Collection

5.5.1 Archival or Formalin-Fixed Paraffin-Embedded (FFPE) Tumor Specimen

If previously-collected FFPE tissue will be submitted, then the following criteria must be met:

- Tissue must have been collected within 6 months prior to registration
- FFPE tumor tissue block(s) must be submitted. The optimal block is at least 70% tumor. Specimen size requirement is as follows:
  - Surface area: 25 mm^2 is optimal. Minimum is 5 mm^2.
  - Volume: 1 mm^3 optimal. Minimum volume is 0.2 mm^3, however the success of DNA extraction decreases at suboptimal tissue volume.

If an existing block cannot be submitted, the following are requested, if available:

- One (1) H&E slide (3-5 μm)
- Thirty to fifty (30 – 50) 10 μm unstained air-dried uncharged slides (preferred). If not feasible, then a minimum of twenty (20) 10 μm unstained air-dried uncharged slides should be submitted with a minimum tumor content of at least 30%. **Submission of specimens with <30% tumor content may not provide sufficient material for analysis**.

Process and number slides sequentially (e.g., H&E stained slide should be created first and labeled with "1," and additional unstained slides should be processed next and be labeled 2 – n).

See Section 5.4.2 for labeling instructions.

5.5.2 Collection of Snap-Frozen Biopsies

1. Tissue should be frozen as soon as possible. Optimally, freeze within 30 minutes from resection.
2. Prior to tissue collection:
    a. Label cryovial(s) according to instructions in Section 5.4.2.
    b. Place cryovial(s) on dry ice to freeze. The vials should appear frosty when ready.
3. Immediately place tissue in foil and allow to completely freeze (using either direct contact with dry ice, or liquid nitrogen vapor).
4. Gently remove the frozen tissue from the foil. If the tissue is sticking to the foil, then gently run a finger over the back of the foil to loosen the tissue.
5. Using clean forceps place each tissue core in a separate pre-chilled cryovial. Tissue should move freely in the vial.
6. Place the tissue in a -70 to -80°C freezer. Keep frozen until shipment to the EET Biobank.

5.5.3 Blood Collection

5.5.3.1 Collection of Blood in EDTA Tubes for Whole Blood Processing

1. Label EDTA tubes according to the instructions in Section 5.4.2.
2. Collect 10 mL blood in EDTA tube(s) and gently invert tube to mix.
3. Ship on day of collection (whenever possible) according to instructions in Section 5.6.
4. If blood cannot be shipped on the day of collection (e.g., a late scheduled collection), then refrigerate until shipment.

5.5.3.2 Collection of Blood in EDTA Tubes for PK Processing

1. Label EDTA tubes according to the instructions in Section 5.4.2.
2. Collect 4 mL blood in EDTA tube(s) (Becton Dickinson Catalog # 367844 or 367862, Franklin Lakes, NJ) and gently invert tube to mix.
3. Place samples immediately **on ice** after collection; samples must be processed **within 30 minutes**.
4. Invert sample 8-10 times immediately before processing.
5. Centrifuge at ~1300 xg for 10 minutes in swinging bucket (SW) or 15 minutes in a fixed angel (FA) rotor at 4ºC in a refrigerated centrifuge. Make sure that the centrifuge reaches speed and is maintained throughout the entire spin.
6. Carefully remove tube from centrifuge.
7. Using a pipette, transfer equal aliquots of plasma into 2-3 labeled 1.2 mL cryovials

(e.g., preferred are external thread, conical self-standing vials like Corning™ 430658), not exceeding ~1 mL per cryovial.

8. Label samples as Rogaratinib PK, including study number (NCI10411), unique patient ID (assigned by the consortium), date of collection, draw time, and time point.
9. Store plasma samples at -70ºC or below until shipment or transfer to Johns Hopkins.

5.6 Shipping Specimens from Clinical Site to the EET Biobank

5.6.1 General Shipping Information

The shipping container sent with kit contents should be used to ship frozen specimens to the EET Biobank. Fresh blood should be shipped at ambient temperature to the EET Biobank. In winter months, please include extra insulation, such as bubble wrap, inside the shipping container.

5.6.1.1 Required Forms for Specimen Submissions:

Each document submitted with the specimen must be labeled with a label printed from the STS, or the Universal ID and Patient Study ID.

Tissue	Required Forms
Archival (if available)	1. Shipping List<br>2. Corresponding Pathology Report
New Biopsy	1. Shipping List<br>2. Tissue Biopsy Verification Form<br>3. Diagnostic Pathology Report<br>4. Operative and/or Radiology Report
Blood	1. Shipping List

5.6.2 Specimen Shipping Instructions

Frozen specimens and archival (FFPE) tissue (if available) may be shipped on Monday through Thursday.

Fresh blood may be shipped on Monday through Friday. Please select “Saturday Delivery” when shipping fresh blood on a Friday.

5.6.2.1 Shipping of FFPE Blocks and Glass Slides

1. Before packaging blocks or slides, verify that each specimen is labeled according to Section 5.4.2.2.
2. Blocks should be placed in a hard-sided container, preferably a special block holder, to protect the specimen. Glass slides are to be placed in plastic slide holders. Place tissue

paper on top of the separated slides prior to closing the slide holder to reduce slide movement during shipment.

3. Place the blocks or slides in a reinforced cardboard shipping box with appropriate packaging filler to minimize movement of specimens within the shipping box.
4. Include a copy of the forms listed above and a shipping manifest from the Specimen Tracking System with each shipment.
5. Please include a cold pack when shipping on hot days and extra insulation on cold days.
6. Ship specimens to the address listed below. FedEx Priority Overnight is strongly recommended to prevent delays in package receipt.

5.6.2.2 Shipping Frozen Specimens in a Single-Chamber Kit

1. Before packaging specimens, verify that each specimen is labeled according to the instructions in Section 5.4.2.1.
2. Place the specimens in zip-lock bags. Use a separate zip-lock bag for each specimen type and time point.
3. Place the zip-lock bags in the biohazard envelope containing absorbent material. Expel as much air as possible and seal securely.
4. Put the secondary envelope into a Tyvek envelope. Expel as much air as possible and seal securely.
5. Place frozen specimens in the kit compartment with dry ice. Layer the bottom of the compartment with dry ice until it is approximately one-third full. Place the frozen specimens on top of the dry ice. Cover the specimens with the dry ice until the compartment is almost completely full. When packaging specimens, ensure that you leave enough room to include at least 5 pounds of dry ice in the shipment.
6. Insert a copy of the required forms into a plastic bag and place in the kit chamber.
7. Place the Styrofoam lid on top to secure specimens during shipment. Do not tape the inner chamber shut.
8. Close the outer lid of the Specimen Procurement Kit and tape it shut with durable sealing tape. Do not completely seal the container.
9. Complete a FedEx air bill and attach to top of shipping container.
10. Complete a dry ice label.
11. Attach the dry ice label and an Exempt Human Specimen sticker to the side of the shipping container.
12. Ship specimens via overnight courier to the address below. FedEx Priority Overnight is strongly recommended to prevent delays in package receipt.

5.6.2.3 Shipping Blood Using Supplies Provided by the Institution

1. Before packaging specimens, verify that the collection tube is labeled according to the instructions in 5.4.2.
2. Place the blood collection tube into a zip-lock bag.
3. Place the zip-lock bag in the biohazard envelope containing absorbent material. Expel as much air as possible and seal securely.
4. Place the biohazard envelope into a Tyvek envelope. Expel as much air as possible and seal securely.

5. Place specimen and a copy of the shipping manifest into a sturdy shipping container. In winter months, please use an insulated container and include extra insulation, such as bubble wrap, inside the container to prevent specimens from freezing.
6. Close the container and tape shut.
7. Attach a shipping label to the top of shipping container.
8. Attach an Exempt Human Specimen sticker to the side of the shipping container.
9. Ship specimens via overnight courier to the address below. FedEx Priority Overnight is strongly recommended to prevent delays in package receipt.

5.6.3 Shipping Address

Ship to the EET Biobank. Ship fresh blood specimens the same day of specimen collection. Do not ship specimens the day before a holiday.

FedEx Priority Overnight service is very strongly preferred.

NOTE: The EET Biobank FedEx Account will not be provided to submitting institutions. There is no central Courier account for this study. Sites are responsible for the cost of shipments to the EET Biobank.

5.6.4 Contact Information for Assistance

For all queries, please contact the EET Biobank.

5.7 Shipping Specimens from Clinical Site to the JHU SKCCC Analytical Pharmacology Core Lab (APC) for the ChOP-KC

5.7.1 General Shipping Information

Specimens should be stored through the end of Cycle 3 Day 1 and shipped as a batch by participant (more than one participant/shipment is acceptable if the site has >1 participant on-study). A participant's samples should be shipped to the APC lab within 2 weeks of the last sample's collection date. (i.e., if C1D28 sample is collected on 9/1/2020, all of that participant's samples should be at the APC lab by 9/15/2020). The APC lab may contact the study team to request shipment off-schedule.

Please ship 1-2 aliquots to the APC laboratory. Once receipt is confirmed, the back-up aliquot may be shipped. The back-up can be shipped at a later date with subsequent batches.

5.7.2 Preparing the Specimen Shipment Instructions

1. Samples should be stored in cardboard boxes (5 1/8” x 5 1/8” x 2”, LxWxH) with dividers.

(*e.g.*, VWR Box item number is 82021-114; divider item number is 82007-154.)

2. Please organize the samples by Patient and Time point in the box.
3. Do not store in plastic bags (they break on dry-ice and labels will detach).
4. A copy of each of the pharmacokinetic sample collection forms for the respective patients or a sample list should be included with each shipment. To prevent problems with illegible writing on tubes, consider numbering them (in addition to sample label) and numbering samples on the sample sheet.
5. Note the study number, PI, and the drugs used/to be measured.
6. A name, phone number and email address should be included with samples so that receipt can be acknowledged.
7. Please notify the lab by email at least 24 hours prior to shipment.

5.7.3 Specimen Shipping Instructions

1. Samples collected at Johns Hopkins University can be transferred utilizing the current SOPs.
2. All samples should be shipped via overnight express courier in insulated containers with enough dry ice to maintain the samples in a frozen state.
3. Overnight shipments should occur on Monday through Wednesday (Tuesday is the preferred day) except when the following day is a holiday.

5.7.4 Shipping Address

Ship to the core laboratory.

5.8 Biomarker Plan

List of Biomarker Assays in Order of Priority

Note for participating sites: Please see Section 5.1 for details on specimens to collect. The specimens tested are not always the same specimens that are submitted by the site, as processing of blood and tissue will occur at the Biobank prior to testing.

Priority	Biomarker Name	Assay (CLIA: Y/N)	Use in the Trial and Purpose	Specimens Tested	Collection Time Points	Mandatory or Optional	Assay Laboratory and Lab PI
**Tissue-based Biomarkers**							
N/A	Mutations and fusions in FGFR 1, 2, 3, and 4	NGS<br><br>CLIA: Y	Integral<br><br>Eligibility for Cohort A	N/A	Pre-enrollment	M	Local Testing (e.g., Foundation One)
N/A	SDH	IHC<br><br>CLIA: Y	Integral<br><br>Eligibility for Cohort B	N/A	Pre-enrollment	M	Local Testing
1	WES	NGS<br><br>CLIA: N	Integrated<br><br>To confirm the presence of FGFR mutations and fusions<br><br>To identify other genetic alterations that might be associated with response and/or potential mechanisms of resistance	DNA from archival FFPE or Fresh Frozen Tumor	Pre-treatment or archival (if pre-treatment unavailable),<br><br>Progression	M<br><br><br><br><br>O	NCLN Genomics Laboratory or MoCha, Frederick National Laboratory for Cancer Research (FNLCR)
2	RNAseq	NGS<br><br>CLIA: N	Integrated<br><br>To examine the correlation between FGFR mutations and changes in FGFR expression<br><br>To examine adaptive activation of signaling pathways as a potential mechanism of resistance	RNA from archival FFPE or Fresh Frozen Tumor	Pre-treatment or archival (if pre-treatment unavailable),<br><br>Progression	M<br><br><br><br><br>O	NCLN Genomics Laboratory or MoCha, Frederick National Laboratory for Cancer Research (FNLCR)

Priority	Biomarker Name	Assay (CLIA: Y/N)	Use in the Trial and Purpose	Specimens Tested	Collection Time Points	Mandatory or Optional	Assay Laboratory and Lab PI
**Blood-based Biomarkers**							
1	WES	NGS<br><br>CLIA: N	Integrated<br><br>Germline control	Germline DNA from blood in EDTA	Pre-treatment	M	NCLN Genomics Laboratory or MoCha, Frederick National Laboratory for Cancer Research (FNLCR)
2	Pharmacokinetics (PK)	GLP<br><br>CLIA: N	Exploratory<br><br>To explore rogaratinib exposure with pharmacodynamics effects (i.e., clinical response, toxicity, and markers of FGFR pathway inhibition).	Plasma	Pre-dose* (pre-treatment [prior to C1D1], C1D15, C2D1, C2D15, and C3D1), the clinic visit after a dose reduction, and at the time of Progression<br><br>*Compliance should be documented as well as the last dose prior to the specimen collection.	M (all other timepoints)<br><br>O (at Progression)	JHU SKCCC Analytical Pharmacology Core Lab for the ChOP-KC

5.9 Integral Laboratory Studies

5.9.1 Mutations and fusions in FGFR 1, 2, 3, and 4

5.9.1.1 Specimen(s) Receipt and Processing at Local Laboratory
For Cohort A, local NGS report documenting FGFR alteration must be submitted and reviewed/approved by the study team prior to registration.

5.9.1.2 Site(s) Performing Correlative Study
This assay will be performed at a local laboratory (*e.g.*, Foundation One) as part of SOC prior to enrollment.

5.9.1.3 Contact information for notification of specimen shipment

Not applicable.

5.9.2 SDH

5.9.2.1 Specimen(s) Receipt and Processing at Local Laboratory
For Cohort B, pathology reports documenting SDH-def GIST must be submitted and reviewed/approved by the study team prior to registration.

5.9.2.2 Site(s) Performing Correlative Study
This assay will be performed at a local laboratory as part of SOC prior to enrollment.

5.9.2.3 Contact information for notification of specimen shipment

Not applicable.

5.10 Integrated Correlative Studies

5.10.1 Whole Exome Sequencing (WES)

5.10.1.1 Specimen(s) Receipt and Processing at the EET Biobank

Available archival FFPE tissue will be received as an FFPE tissue block or as H&E-stained and unstained slides. FFPE tissue blocks and stained slides will be stored at room temperature, and unstained slides will be vacuum sealed and banked in refrigerated storage until processing.

H&E stained slides will undergo a pathology QA review to assess tumor content and annotate for macrodissection, when needed. Following macrodissection, tumor tissue from unstained slides will be scraped for co-extraction of DNA and RNA. The nucleic acids will be analyzed to determine concentration and quality prior to storage in a -80°C freezer. The remaining FFPE block and H&E stained slides will be stored at room temperature. Aliquots of DNA will be shipped to the central sequencing laboratory for analysis.

Frozen tissue received for pre-treatment and progression time points will be barcoded and stored in a liquid nitrogen vapor phase freezer until nucleic acids are extracted.

Prior to extraction, an H&E stained slide will be created from frozen tissue. All H&E stained slides will undergo a pathology QA review to assess tumor content. DNA and RNA will be co-extracted from frozen tissue, and nucleic acids will be analyzed to determine concentration and quality prior to storage in a -80°C freezer. All remaining frozen tissue will be stored in a liquid nitrogen vapor phase freezer. Aliquots of DNA will be shipped to the central sequencing laboratory for analysis.

DNA will be extracted from blood collected in EDTA tubes at pre-treatment. DNA will be quantitated, and then stored in a -80°C freezer until shipping for analysis.

5.10.1.2 Site(s) Performing Correlative Study

This assay will be performed at the NCLN Genomics Laboratory or MoCha, Frederick National Laboratory for Cancer Research (FNLCR).

5.10.1.3 Shipment of Specimens from the EET Biobank to Site Performing Correlative Study

Specimens will be shipped from the EET Biobank to one of the laboratories designated in Section 5.10.1.2.

5.10.1.4 Contact Information for Notification of Specimen Shipment

5.10.2 <u>RNA Sequencing</u>

5.10.2.1 Specimen(s) Receipt and Processing at the EET Biobank

Available archival FFPE tissue will be received as an FFPE tissue block or as H&E-stained and unstained slides. FFPE tissue blocks and stained slides will be stored at room temperature, and unstained slides will be vacuum sealed and banked in refrigerated storage until processing.

H&E stained slides will undergo a pathology QA review to assess tumor content and annotate for macrodissection, when needed. Following macrodissection, tumor tissue from unstained slides will be scraped for co-extraction of DNA and RNA. The nucleic acids will be analyzed to determine concentration and quality prior to storage in a -80°C freezer. The remaining FFPE block and H&E stained slides will be stored at room temperature. Aliquots of RNA will be shipped to the central sequencing laboratory for analysis.

Frozen tissue received for pre-treatment and progression time points will be barcoded and stored

in a liquid nitrogen vapor phase freezer until nucleic acids are extracted.

Prior to extraction, an H&E stained slide will be created from frozen tissue. All H&E stained slides will undergo a pathology QA review to assess tumor content. DNA and RNA will be co-extracted from frozen tissue, and nucleic acids will be analyzed to determine concentration and quality prior to storage in a -80°C freezer. All remaining frozen tissue will be stored in a liquid nitrogen vapor phase freezer. Aliquots of RNA will be shipped to the central sequencing laboratory for analysis.

5.10.2.2 Site(s) Performing Correlative Study

This assay will be performed at the NCLN Genomics Laboratory or MoCha, Frederick National Laboratory for Cancer Research (FNLCR).

5.10.2.3 Shipment of Specimens from the EET Biobank to Site Performing Correlative Study

Specimens will be shipped from the EET Biobank to one of the laboratories designated in Section 5.10.2.2.

5.10.2.4 Contact Information for Notification of Specimen Shipment

5.11 Exploratory Correlative Studies

5.11.1 <u>Pharmacokinetics (PK)</u>

5.11.1.1 Specimen(s) Receipt and Processing at the JHU SKCCC Analytical Pharmacology Core Lab for the ChOP-KC

Rogaratinib will be extracted from plasma isolated from blood collected in EDTA tubes at pre-treatment (prior to C1D1, C1D15, C2D1, C2D15, and C3D1) and at the time of Progression at the sites. Additional trough samples may be collected after a dose reduction to correlate the exposure at tolerable doses. Plasma will be stored in a -80°C freezer until shipping for analysis.

NOTES regarding the PK collection:

- The dates of the PK samples are when the physical exams are scheduled. If a physical exam is shifted due to a holiday or inclement weather, the PK should be collected on that day (and not the exact day listed above).
- If rogaratinib has been held for non-toxicity reasons (Section 7) for longer than 6 doses (3 days), the PK sample should not be collected.
- If rogaratinib has been held for toxicity reasons (Section 7), the PK sample should not be collected with documentation of the last dose administered (date and time).

5.11.1.2 Site(s) Performing Correlative Study

This assay will be performed at the JHU SKCCC Analytical Pharmacology Core Lab for the ChOP-KC.

5.11.1.3 Contact information for notification of specimen shipment

6. TREATMENT PLAN

6.1 Agent Administration

Treatment will be administered on an outpatient basis. Reported adverse events and potential risks are described in Section 10. Appropriate dose modifications are described in Section 7. No investigational or commercial agents or therapies other than those described below may be administered with the intent to treat the patient's malignancy.

**Regimen Description**					
**Agent**	**Premedications; Precautions**	**Dose**	**Route**	**Schedule**	**Cycle Length**
Rogaratinib (BAY 1163877)	N/A	800 mg	PO	BID Day 1 – Day 28	28 days (4 weeks)
PO = Orally, BID =Twice a Day					

The patient will be requested to maintain a medication diary of each dose of medication (see Appendix E). The medication diary will be returned to clinic staff at the end of each course.

6.1.1 CTEP IND Agent

6.1.1.1 Rogaratinib (BAY 1163877) (NSC 804782)

Patients will receive rogaratinib (BAY 1163877) orally 800mg po BID for a 28-day cycle. Rogaratinib (BAY 1163877) may be administered with or without food. If a dose is missed, it can be taken within 3 hours of the usual dosing time. If it is after 3 hours of the usual dosing time, the dose should be skipped and the next dose should be taken at the usual time. Vomited doses should not be replaced. Rogaratinib (BAY 1163877) should be swallowed whole. Do not chew. Patients should be instructed to bring their morning dose of rogaratinib (BAY 1163877) with them to the clinic on Day 1 of Cycles 1-3 and Day 15 of Cycles 1-2.

6.2 General Concomitant Medication and Supportive Care Guidelines

Because there is a potential for interaction of rogaratinib (BAY 1163877) with other concomitantly administered drugs, the case report form must capture the concurrent use of all other drugs, over-the-counter medications, or alternative therapies. The Principal Investigator should be alerted if the patient is taking any agent known to affect or with the potential for drug interactions. The study team should check a frequently-updated medical reference for a list of drugs to avoid or minimize use of. Appendix C (Patient Drug Interaction Handout and Wallet Card) should be provided to patients if available.

Rogaratinib (BAY 1163877) exhibited characteristics of a P-gp substrate (Rogaratinib Investigator's Brochure, 2019). Additionally, it was shown that rogaratinib (BAY 1163877) exhibited inhibitory potential on P-gp and BCRP. Oxidative metabolism of rogaratinib (BAY 1163877) is mainly catalyzed by CYP3A4 and to a lower extent by CYP2C9. Hence, concomitant use of strong inhibitors as well as of strong inducers of CYP3A4 may result in altered plasma exposure levels of rogaratinib (BAY 1163877). Based on *in vitro* study results indicating inhibition of CYP3A4, P-gp and BCRP, narrow therapeutic index drugs that are CYP3A4, P-gp, BCRP, MATE1, and MATE2K substrates and strong inhibitors and inducers of CYP3A4 (*e.g.,* alfentanil, cyclosporine, dihydroergotamine, ergotamine, fentanyl, pimozide, quinidine, sirolimus, and tacrolimus) should be avoided. Use caution with strong inhibitors and inducers of P-gp.

Concomitant administration of medications that prolong QT/QTc interval is prohibited in accordance with the published FDA guidance "E14 Clinical Evaluation of QT/QTc Interval Prolongation and Proarrhythmic Potential for Non-Antiarrhythmic Drugs".

6.3 Duration of Therapy

In the absence of treatment delays due to adverse event(s), treatment may continue until one of the following criteria applies:

- Disease progression
- Intercurrent illness that prevents further administration of treatment
- Unacceptable adverse event(s)
- Patient decides to withdraw from the study
- General or specific changes in the patient's condition render the patient unacceptable for further treatment in the judgment of the investigator
- Clinical progression
- Patient non-compliance
- Pregnancy

  - All women of child bearing potential should be instructed to contact the investigator immediately if they suspect they might be pregnant (*e.g.*, missed or late menstrual period) at any time during study participation.

  - The investigator must immediately notify CTEP in the event of a confirmed pregnancy in a patient participating in the study.

- Termination of the study by sponsor

- The drug manufacturer can no longer provide the study agent

The reason(s) for protocol therapy discontinuation, the reason(s) for study removal, and the corresponding dates must be documented in the Case Report Form (CRF).

6.4 Duration of Follow-Up

Patients will be followed for 30 days after removal from study or until death, whichever occurs first. Patients removed from study for unacceptable adverse event(s) will be followed until resolution or stabilization of the adverse event.

7. DOSING DELAYS/DOSE MODIFICATIONS

- Toxicity modifications are for adverse events (AEs) thought to be at least possibly related to agent.
- A delay of up to 3 weeks (21 days) is permitted from the scheduled cycle start date to allow for toxicity thought to be at least possibly related to agent to resolve such that criteria for allowing continuation of therapy as detailed below are met. If toxicity has not resolved by that time, study treatment should be discontinued.
- Only two dose reductions will be allowed. If AE recurs requiring dose reduction then the patient will be removed from protocol.

Dose Level	Rogaratinib (BAY 1163877) Dose
-2	400 mg, PO, BID
-1	600 mg, PO, BID
0	800 mg, PO, BID

ALT or AST elevations	Management/Next Dose for Rogaratinib (BAY 1163877)
Grade 0-2	No change in dose
Grade 3	Hold* until ≤ Grade 2. Resume at one dose level lower.**
Grade 4	Off protocol therapy
*Patients requiring a delay of >3 weeks should go off protocol therapy.	

ALT or AST elevations	Management/Next Dose for Rogaratinib (BAY 1163877)
**Patients requiring > two dose reductions should go off protocol therapy.<br><br>Patients with ALT or AST > 3x ULN with bilirubin > 2x ULN without findings of cholestasis (i.e. alk phos < 2x ULN) or other etiology for LFT changes should go off protocol.	

Hyperphosphatemia:
Dose modifications of rogaratinib for elevated serum phosphate levels (≥ 7 mg/dL) and management guidance for hyperphosphatemia are presented in the Table below. Dose interruptions for hyperphosphatemia for >21 consecutive days, requires treatment discontinuation.

Serum phosphate	Countermeasures
Initial phosphate value is abnormally high, but less than 7 mg/dL	Continue rogaratinib at the same dose.<br>Consider low phosphate diet [a] and / or initiate phosphate chelators.
≥ 7 mg/dL for two weeks despite phosphate lowering treatment	Hold rogaratinib and increase dose of phosphate chelators until recovery below 7 mg/dL.<br>Re-start rogaratinib at the same dose level and continue phosphate chelators.
≥ 7 mg/dL despite optimal phosphate lowering treatments and two rogaratinib treatment interruptions on the same dose within four weeks	Hold rogaratinib and continue phosphate chelators until recovery below 7 mg/dL.<br>Re-start rogaratinib, but at one dose level lower and continue phosphate chelators.
≥ 7 mg/dL despite optimal phosphate lowering treatments and two rogaratinib treatment interruptions within four weeks at 400 mg b.i.d.	Hold rogaratinib and continue phosphate chelators until recovery below 7 mg/dL.<br>Re-start rogaratinib at 400 mg b.i.d. and continue phosphate chelators.

b.i.d. = Twice daily, *bis in die.*
a: Low phosphate diet can be considered if the patient's nutritional status is not affected.

In case phosphate chelators are not tolerated, low-phosphorus diet should be considered.

For patients with elevated serum phosphate levels ≥ 7 mg/dL, serum phosphate level and standard single 12-lead ECG has to be checked weekly until resolution (serum phosphate < 7 mg/dL).

For patients with hypocalcemia of CTCAE Grade ≥ 2, an additional standard single 12-lead ECG has to be obtained on the day of detection of hypocalcemia and should be repeated as clinically indicated.

Hyperphosphatemia may be associated with soft tissue mineralization (e.g., cutaneous calcification). Any patient with newly diagnosed soft-tissue mineralization suspected to be caused by rogaratinib should permanently discontinue the study treatment.

Retinal disorders:

Patients who experience any decrease in visual acuity, or symptomatic or asymptomatic retinal disorders including retinal detachment / retinal pigment epithelial detachment (RPED) / serous retinopathy / retinal vein occlusion (Grade 1 classified analog to CTCAE) have to undergo ophthalmologic examinations on Day 1 of every cycle.

Patients that experience any decrease in visual acuity, or symptomatic or asymptomatic retinal disorders including retinal detachment / retinal pigment epithelial detachment / serous retinopathy / retinal vein occlusion classified analog to CTCAE as Grade 2 or higher have to be permanently discontinued from study treatment.

**Neutropenia or thrombocytopenia**	**Management/Next Dose for Rogaratinib (BAY 1163877)**
Grade 0-2	No change in dose
Grade 3	Hold* until ≤Grade 2. Resume at one dose level lower.**
Grade 4	Off protocol therapy
*Patients requiring a delay of >3 weeks should go off protocol therapy.<br>**Patients requiring > two dose reductions should go off protocol therapy.	

**Other non-hematologic AEs°**	**Management/Next Dose for Rogaratinib (BAY 1163877)**
Grade 0-2	No change in dose
Grade 3	Hold* until ≤ Grade 2. Resume at one dose level lower**.
Grade 4	Off protocol therapy
*Patients requiring a delay of >3 weeks should go off protocol therapy.<br>**Patients requiring > two dose reductions should go off protocol therapy. Allow 48 hours for optimal management of grade 3 events such as other electrolyte abnormalities, diarrhea, nausea or emesis.	

° Excluding alopecia. Nausea and vomiting only if refractory to anti-emetics, diarrhea refractory to anti-diarrheal agents.
It is recommended that investigators optimize treatment for nausea, vomiting and diarrhea.

8. PHARMACEUTICAL INFORMATION

A list of the adverse events and potential risks associated with the investigational agent administered in this study can be found in Section 10.1.

8.1 CTEP IND Agent

8.1.1 Rogaratinib (BAY 1163877) (NSC 804782)

Chemical Name or Amino Acid Sequence: 4-{[4-amino-6-(methoxymethyl)-5-(7-methoxy-5-methyl-1-benzothiophen-2-yl)pyrrolo[2,1-f][1,2,4]triazin-7-yl]methyl}piperazin-2-one

Other Names: BAY 1163877 (rogaratinib), BAY 1213802 (rogaratinib hydrochloride hydrate)

Classification: Fibroblast growth factor receptors (FGFR) inhibitor

CAS Registry Number: 1443530-05-09

Molecular Formula (BAY1163877): $C_{23}H_{26}N_6O_3S$ **M.W.:** 466.56 g/mol
Molecular Formula (BAY1213802): $C_{23}H_{26}N_6O_3S \cdot HCl \cdot H_2O$ **M.W.:** 521.04 g/mol

Approximate Solubility: BAY 1213802 is slightly soluble in water, very slightly soluble in ethanol, sparingly soluble in polyethylene glycol (PEG) 400.

Mode of Action: Rogaratinib (BAY 1163877) is a small molecule kinase inhibitor of fibroblast growth factor receptor (FGFR) 1, 2, 3, and 4. FGFRs drive crucial developmental signaling pathways, which are responsible for many functions of the tumor cell, including cell proliferation, survival, and migration.

Description: BAY 1213802 is a white to yellow solid that is isolated as a hydrate. BAY 1231802 is the active pharmaceutical ingredient and the hydrochloride hydrate of rogaratinib.

How Supplied: Rogaratinib (BAY 1163877) is supplied by Bayer HealthCare AG and distributed by the Pharmaceutical Management Branch, CTEP/DCTD/NCI as 200 mg coated immediate release tablets in bottles containing 56 tablets. The 200 mg tablets are oblong, red coated tablets with a size of 16 x 8 mm. Excipients include cellulose microcrystalline, lactose monohydrate, crospovidone, copovidone, magnesium stearate, silica colloidal anhydrous, and lacquer red (hypromellose, macrogol, titanium dioxide, and ferric oxide red).

Storage: Do not store above 25°C. Do not freeze.

If a storage temperature excursion is identified, promptly return rogaratinib to below 25°C and quarantine the supplies. Provide a detailed report of the excursion (including documentation of

temperature monitoring and duration of the excursion) to the PMB for determination of suitability.

Stability: Stability studies are ongoing. Dispense in the original container.

Route and Method of Administration: Take by mouth with or without food with a glass of water. Swallow whole. Do not chew. If a dose is missed, take within 3 hours of the usual dosing time. If it is after 3 hours of the usual dosing time, skip the dose and take the next dose at the usual time.

Potential Drug Interactions:
In vitro, rogaratinib (BAY 1163877) is mainly metabolized by CYP3A4 and to a lesser extent by CYP2C9 and CYP1A1. It is a weak inhibitor of CYP2C8, 2C9, UGT1A1, UGT1A4, and UGT1A9. It did not inhibit CYP1A2, 2A6, 2B6, 2C19, 2D6, 2E1, 2J2, 3A4, UGT1A6, UGT2B4, and UGT2B7. However, irreversible inhibition of CYP3A4 was observed after pre-incubation of rogaratinib (BAY 1163877) with human liver microsomes. *In vitro*, rogaratinib (BAY 1163877) did not induce CYP1A2, 3A4, 2B6, or 2C19. Avoid concomitant administration with sensitive substrates/narrow therapeutic index drugs of CYP3A4, and strong inhibitors and inducers of CYP3A4.

In vitro, rogaratinib (BAY 1163877) is a moderate P-gp substrate but not BCRP, OATP1B1, 1B3, or OCT1. Rogaratinib (BAY 1163877) showed weak to moderate inhibition of MATE2K, MATE1, BCRP, BSEP, P-gp, OATP1B1, OATP1B3, and OCT1 *in vitro*. It does not inhibit OAT1, OAT3, and OCT2. Avoid concomitant administration with sensitive substrates/narrow therapeutic index drugs of P-gp BCRP, MATE1, and MATE2K. Use caution with strong inhibitors and inducers of P-gp.

Availability

Rogaratinib (BAY 1163877) (NSC 804782 is an investigational agent supplied to investigators by the Division of Cancer Treatment and Diagnosis (DCTD), NCI.

Rogaratinib (BAY 1163877) (NSC 804782) is provided to the NCI under a Collaborative Agreement between the Pharmaceutical Collaborator and the DCTD, NCI (see Section 13.5).

8.1.2 <u>Agent Ordering and Agent Accountability</u>

8.1.2.1 NCI-supplied agents may be requested by eligible participating Investigators (or their authorized designee) at each participating institution. The CTEP-assigned protocol number must be used for ordering all CTEP-supplied investigational agents. The eligible participating investigators at each participating institution must be registered with CTEP, DCTD through an annual submission of FDA Form 1572 (Statement of Investigator), NCI Biosketch, Agent Shipment Form, and Financial Disclosure Form (FDF). If there are several participating investigators at one institution, CTEP-supplied investigational agents for the study should be ordered under the name of one lead

participating investigator at that institution.

Sites can place orders for PMB-supplied agents only after enrollment onto the study. Please provide the patient ID# when placing an order.

Submit agent requests through the PMB Online Agent Order Processing (OAOP) application. Access to OAOP requires the establishment of a CTEP Identity and Access Management (IAM) account and the maintenance of an "active" account status, a "current" password, and active person registration status. For questions about drug orders, transfers, returns, or accountability, call or email PMB any time. Refer to the PMB's website for specific policies and guidelines related to agent management.

8.1.2.2 Agent Inventory Records – The investigator, or a responsible party designated by the investigator, must maintain a careful record of the receipt, dispensing and final disposition of all agents received from the PMB using the appropriate NCI Investigational Agent (Drug) Accountability Record (DARF) available on the CTEP forms page. Store and maintain separate NCI Investigational Agent Accountability Records for each agent, strength, formulation and ordering investigator on this protocol.

8.1.3 Investigator Brochure Availability

The current versions of the IBs for the agents will be accessible to site investigators and research staff through the PMB OAOP application. Access to OAOP requires the establishment of a CTEP IAM account and the maintenance of an "active" account status, a "current" password and active person registration status. Questions about IB access may be directed to the PMB IB Coordinator via email.

8.1.4 Useful Links and Contacts

- CTEP Forms, Templates, Documents: http://ctep.cancer.gov/forms/
- NCI CTEP Investigator Registration:
- PMB policies and guidelines: http://ctep.cancer.gov/branches/pmb/agent_management.htm
- PMB Online Agent Order Processing (OAOP) application: https://ctepcore.nci.nih.gov/OAOP
- CTEP Identity and Access Management (IAM) account: https://ctepcore.nci.nih.gov/iam/
- CTEP IAM account help:
- IB Coordinator:
- PMB email:
- PMB phone and hours of service:

9. STATISTICAL CONSIDERATIONS

9.1 Study Design/Endpoints

The study will focus on two cohorts of sarcomas with alterations in the FGF axis. The two cohorts will be enrolled and analyzed separately. Cohort A is defined as any sarcoma with documented FGFR alteration as confirmed by NGS. Cohort B is defined as SDH-deficient GIST. No molecular screening is required for SDH-deficient GIST. Rogaratinib (BAY 1163877) will be considered effective in each cohort if a 25% overall response rate (ORR, including CR and PR) is observed.

To achieve this, the study will employ a Simon two-stage optimal design. The Simon two-stage optimal design targeting response rates of 5% *vs.* 25%, with a one-sided type 1 error of 10% and power of 90%, accrues 9 patients at the first stage. With 1 or more responses, it goes on to a second stage for a total of 24 patients. Three or more responses (out of the 24) are required for a positive trial.

Time-to-event endpoints, including PFS, will be estimated using Kaplan-Meier method. Correlative and biomarker objectives will be considered exploratory. Assuming the study moves to the second stage, the maximal width of a 90% confidence interval for any binomial parameter will be no wider than 35.3%, hence estimation of binomial quantities will be fairly accurate. Estimation of continuous biomarker measures will be *via* mean or median as appropriate and standard deviation. There will be limited power to correlate clinical measures with biomarker endpoints with this limited phase 2 sample size, hence those analyses will be considered exploratory.

9.2 Sample Size/Accrual Rate

Sample size: minimum = 18, maximum = 48.

Accrual rate: 2 patients per month per cohort.

PLANNED ENROLLMENT REPORT

DOMESTIC PLANNED ENROLLMENT REPORT (TREATMENT)					
Racial Categories	Ethnic Categories				Total
	Not Hispanic or Latino		Hispanic or Latino		
	Female	Male	Female	Male	
American Indian/ Alaska Native	0	0	0	0	0
Asian	1	1	1	1	4
Native Hawaiian or Other Pacific Islander	0	0	0	0	0
Black or African American	1	1	1	1	4
White	19	17	2	2	40
More Than One Race	0	0	0	0	0
**Total**	21	19	4	4	48

PHS 398 / PHS 2590 (Rev. 03/20 Approved Through 2/28/2023) OMB No. 0925-0001/0002

9.3 Stratification Factors

Not applicable.

9.4 Analysis of Secondary Endpoints

Time-to-event endpoints, including PFS, will be estimated using Kaplan-Meier method. Correlative and biomarker objectives will be considered exploratory. Assuming the study moves to the second stage, the maximal width of a 90% confidence interval for any binomial parameter will be no wider than 35.3%, hence estimation of binomial quantities will be fairly accurate. Estimation of continuous biomarker measures will be via mean or median as appropriate and standard deviation. There will be limited power to correlate clinical measures with biomarker endpoints with this limited phase 2 sample size, hence those analyses will be considered exploratory.

9.5 Analysis of Exploratory Endpoints

Trough samples for rogaratinib will be used to assess exposure-response relationships with various PD endpoints (*i.e.,* clinical response, toxicity, and markers of FGFR pathway inhibition). Rogaratinib concentrations in these samples will be quantitatively measured using liquid chromatography/tandem mass spectrometric (LC/MS/MS) method developed by the Analytical Pharmacology Core Laboratory at the Sidney Kimmel Comprehensive Cancer Center (SKCCC) at Johns Hopkins for the ChOP-KC. For rogaratinib, the average trough concentration (C_{min}) will be calculated as an average of all samples at the same dose. If multiple dose reductions occur for a single patient, the C_{min} at each dose level will be calculated. Exploratory correlative studies with pharmacodynamic (biological endpoints, toxicity and efficacy) will be analyzed using nonparametric statistics. Significance for comparisons will be at the $p<0.05$ level.

9.6 For phase 2 protocols only: Reporting and Exclusions

9.6.1 Evaluation of Toxicity

All patients will be evaluable for toxicity from the time of their first treatment with rogaratinib (BAY 1163877).

9.6.2 Evaluation of Response

All patients included in the study must be assessed for response to treatment, even if there are major protocol treatment deviations. Each patient will be assigned one of the following categories: 1) complete response, 2) partial response, 3) stable disease, 4) progressive disease, 5) early death from malignant disease, 6) early death from toxicity, 7) early death because of other cause, or 9) unknown (not assessable, insufficient data). [Note: By arbitrary convention, category 9 usually designates the “unknown” status of any type of data in a clinical database.]

All of the patients who met the eligibility criteria (with the possible exception of those who received no study medication) should be included in the main analysis of the response rate. Patients in response categories 4-9 should be considered to have a treatment failure (disease progression). Thus, an incorrect treatment schedule or drug administration does not result in

exclusion from the analysis of the response rate. Precise definitions for categories 4-9 will be protocol specific.

All conclusions should be based on all eligible patients. Subanalyses may then be performed on the basis of a subset of patients, excluding those for whom major protocol deviations have been identified (*e.g.*, early death due to other reasons, early discontinuation of treatment, major protocol violations, *etc.*). However, these subanalyses may not serve as the basis for drawing conclusions concerning treatment efficacy, and the reasons for excluding patients from the analysis should be clearly reported.

10. ADVERSE EVENTS: LIST AND REPORTING REQUIREMENTS

Adverse event (AE) monitoring and reporting is a routine part of every clinical trial. The following list of AEs (Section 10.1) and the characteristics of an observed AE (Sections 10.2 and 10.3) will determine whether the event requires expedited reporting via the CTEP Adverse Event Reporting System (CTEP-AERS) **in addition** to routine reporting.

10.1 Comprehensive Adverse Events and Potential Risks List (CAEPR)

10.1.1 CAEPRs for CTEP IND Agent

10.1.1.1 CAEPR for Rogaratinib (BAY 1163877, NSC 804782)

Comprehensive Adverse Events and Potential Risks list (CAEPR) for Rogaratinib (BAY 1163877, NSC 804782)

The Comprehensive Adverse Events and Potential Risks list (CAEPR) provides a single list of reported and/or potential adverse events (AE) associated with an agent using a uniform presentation of events by body system. In addition to the comprehensive list, a subset, the Specific Protocol Exceptions to Expedited Reporting (SPEER), appears in a separate column and is identified with bold and italicized text. This subset of AEs (SPEER) is a list of events that are protocol specific exceptions to expedited reporting to NCI (except as noted below). Refer to the 'CTEP, NCI Guidelines: Adverse Event Reporting Requirements' http://ctep.cancer.gov/protocolDevelopment/electronic_applications/docs/aeguidelines.pdf for further clarification. *Frequency is provided based on 279 patients.* Below is the CAEPR for Rogaratinib (BAY 1163877).

> **NOTE**: Report AEs on the SPEER **ONLY IF** they exceed the grade noted in parentheses next to the AE in the SPEER. If this CAEPR is part of a combination protocol using multiple investigational agents and has an AE listed on different SPEERs, use the lower of the grades to determine if expedited reporting is required.

Version 2.2, February 14, 2023[1]

**Adverse Events with Possible Relationship to Rogaratinib (BAY 1163877) (CTCAE 5.0 Term) [n= 279]**			**Specific Protocol Exceptions to Expedited Reporting (SPEER)**
**Likely (>20%)**	**Less Likely (<=20%)**	**Rare but Serious (<3%)**	
EYE DISORDERS			
	Retinal detachment		
	Retinopathy		
GASTROINTESTINAL DISORDERS			
Diarrhea			***Diarrhea (Gr 2)***
	Dry mouth		***Dry mouth (Gr 2)***
	Mucositis oral		***Mucositis oral (Gr 2)***
	Nausea		***Nausea (Gr 2)***
	Vomiting		
GENERAL DISORDERS AND ADMINISTRATION SITE CONDITIONS			
Fatigue			***Fatigue (Gr 2)***
INVESTIGATIONS			
	Lipase increased		
METABOLISM AND NUTRITION DISORDERS			
Anorexia			***Anorexia (Gr 2)***
Hyperphosphatemia			***Hyperphosphatemia (Gr 2)***
MUSCULOSKELETAL AND CONNECTIVE TISSUE DISORDERS			
	Arthralgia		
NERVOUS SYSTEM DISORDERS			
	Dysgeusia		***Dysgeusia (Gr 2)***
SKIN AND SUBCUTANEOUS TISSUE DISORDERS			
Alopecia			***Alopecia (Gr 2)***
	Dry skin		
	Nail changes		
	Nail loss		
	Nail ridging		

[1]This table will be updated as the toxicity profile of the agent is revised. Updates will be distributed to all Principal Investigators at the time of revision. The current version can be obtained by contacting the Protocol Information Office. Your name, the name of the investigator, the protocol and the agent should be included in the e-mail.

Adverse events reported on Rogaratinib (BAY 1163877) trials, but for which there is insufficient evidence to suggest that there was a reasonable possibility that Rogaratinib (BAY 1163877) caused the adverse event:

BLOOD AND LYMPHATIC SYSTEM DISORDERS - Anemia
EYE DISORDERS - Blurred vision; Eye disorders - Other (macular detachment); Eye disorders - Other (macular edema); Eye disorders - Other (macular telangiectasia); Eye disorders - Other (retinal edema); Eye disorders - Other (retinal pigment epitheliopathy); Eye disorders - Other (scintillating scotoma); Keratitis; Vision decreased
GASTROINTESTINAL DISORDERS - Constipation; Gastrointestinal disorders - Other (colitis ischemic);

Pancreatitis
GENERAL DISORDERS AND ADMINISTRATION SITE CONDITIONS - Fever; General disorders and administration site conditions - Other (general physical health deterioration)
INFECTIONS AND INFESTATIONS - Mucosal infection; Paronychia; Rhinitis infective
INVESTIGATIONS - Alanine aminotransferase increased; Alkaline phosphatase increased; Aspartate aminotransferase increased; Blood bilirubin increased; Creatinine increased; GGT increased; Investigations - Other (calcium phosphate product increased); Neutrophil count decreased
METABOLISM AND NUTRITION DISORDERS - Hypercalcemia; Hypoglycemia; Hyponatremia
NEOPLASMS BENIGN, MALIGNANT AND UNSPECIFIED (INCL CYSTS AND POLYPS) - Neoplasms benign, malignant and unspecified (incl cysts and polyps) - Other (pharyngeal cancer)
NERVOUS SYSTEM DISORDERS - Neuralgia
RENAL AND URINARY DISORDERS - Acute kidney injury; Renal and urinary disorders - Other (nephritis)
RESPIRATORY, THORACIC AND MEDIASTINAL DISORDERS - Pneumonitis; Respiratory, thoracic and mediastinal disorders - Other (interstitial lung disease)
SKIN AND SUBCUTANEOUS TISSUE DISORDERS - Pruritus; Purpura; Rash acneiform; Rash maculo-papular; Skin and subcutaneous tissue disorders - Other (cutaneous calcification/soft tissue mineralization)

Note: Rogaratinib (BAY 1163877) in combination with other agents could cause an exacerbation of any adverse event currently known to be caused by the other agent, or the combination may result in events never previously associated with either agent.

10.2 Adverse Event Characteristics

- **CTCAE term (AE description) and grade:** The descriptions and grading scales found in the revised NCI Common Terminology Criteria for Adverse Events (CTCAE) version 5.0 will be utilized for AE reporting. All appropriate treatment areas should have access to a copy of the CTCAE version 5.0. A copy of the CTCAE version 5.0 can be downloaded from the CTEP website http://ctep.cancer.gov/protocolDevelopment/electronic_applications/ctc.htm.

- **For expedited reporting purposes only:**
  - AEs for the agent that are ***bold and italicized*** in the CAEPR (*i.e.*, those listed in the SPEER column, Section 10.1) should be reported through CTEP-AERS only if the grade is above the grade provided in the SPEER.

- **Attribution** of the AE:
  - Definite – The AE *is clearly related* to the study treatment.
  - Probable – The AE *is likely related* to the study treatment.
  - Possible – The AE *may be related* to the study treatment.
  - Unlikely – The AE *is doubtfully related* to the study treatment.
  - Unrelated – The AE *is clearly NOT related* to the study treatment.

10.3 Expedited Adverse Event Reporting

10.3.1 Rave-CTEP-AERS Integration

The Rave Cancer Therapy Evaluation Program Adverse Event Reporting System (CTEP-AERS) integration enables evaluation of post-baseline AEs entered in Rave to determine whether they require expedited reporting, and facilitates entry in CTEP-AERS for those AEs requiring expedited reporting.

All AEs that occur after baseline are collected in Medidata Rave using the Adverse Event form, which is available for entry at each treatment or reporting period, and used to collect AEs that start during the period or persist from the previous reporting period. CRA will enter AEs that occur prior to the start of treatment on a baseline form that is not included in the Rave-CTEP-AERS integration. AEs that occur prior to enrollment must begin and end on the baseline Adverse Event form and should not be included on the standard Adverse Events form that is available at treatment unless there has been an increase in grade.

Prior to sending AEs through the rules evaluation process, site staff should verify the following on the Adverse Event form in Rave:

- The reporting period (course/cycle) is correct, and
- AEs are recorded and complete (no missing fields) and the form is query-free (fields added to the form during study build do not need to be query-free for the integration call with CTEP-AERS to be a success).

The CRA reports AEs in Rave at the time the Investigator learns of the event. If the CRA modifies an AE, it must be re-submitted for rules evaluation.

Upon completion of AE entry in Medidata Rave, the CRA submits the AE for rules evaluation by completing the Expedited Reporting Evaluation form. Both NCI and protocol-specific reporting rules evaluate the AEs submitted for expedited reporting. A report is initiated in CTEP-AERS using information entered in Medidata Rave for AEs that meet reporting requirements. The CRA completes the report by accessing CTEP-AERS via a direct link on the Medidata Rave Expedited Reporting Evaluation form.

In the rare occurrence that internet connectivity is lost, a 24-hour notification is to be made to CTEP by telephone. Once Internet connectivity is restored, the 24-hour notification that was phoned in must be entered immediately into CTEP-AERS using the direct link from Medidata Rave.

Additional information about the CTEP-AERS integration is available on the CTSU website:

- Study specific documents: Protocols > Documents > Education and Promotion, and

- Expedited Safety Reporting Rules Evaluation user guide: Resources > CTSU Operations Information > User Guides & Help Topics.

NCI requirements for SAE reporting are available on the CTEP website:

- NCI Guidelines for Investigators: Adverse Event Reporting Requirements is available at https://ctep.cancer.gov/protocolDevelopment/electronic_applications/docs/aeguidelines.pdf.

10.3.2 Distribution of Adverse Event Reports

CTEP-AERS is programmed for automatic electronic distribution of reports to the following individuals: Principal Investigator and Adverse Event Coordinator(s) (if applicable) of the Corresponding Organization or Lead Organization, the local treating physician, and the Reporter and Submitter. CTEP-AERS provides a copy feature for other e-mail recipients.

10.3.3 Expedited Reporting Guidelines

Use the NCI protocol number and the protocol-specific patient ID assigned during trial registration on all reports.

Note: A death on study requires both routine and expedited reporting, regardless of causality as long as the death occurred within 30 days after the last administration of the investigational agent. Attribution to treatment or other cause must be provided.

Death due to progressive disease should be reported as **Grade 5 "Disease progression"** in the system organ class (SOC) "General disorders and administration site conditions." Evidence that the death was a manifestation of underlying disease (*e.g.*, radiological changes suggesting tumor growth or progression: clinical deterioration associated with a disease process) should be submitted.

Phase 1 and Early Phase 2 Studies: Expedited Reporting Requirements for Adverse Events that Occur on Studies under an IND/IDE within 30 Days of the Last Administration of the Investigational Agent/Intervention [1, 2]

**FDA REPORTING REQUIREMENTS FOR SERIOUS ADVERSE EVENTS (21 CFR Part 312)**<br>**NOTE:** Investigators **MUST** immediately report to the sponsor (NCI) **ANY** Serious Adverse Events, whether or not they are considered related to the investigational agent(s)/intervention (21 CFR 312.64)<br>An adverse event is considered serious if it results in **ANY** of the following outcomes:<br>1) Death<br>2) A life-threatening adverse event<br>3) An adverse event that results in inpatient hospitalization or prolongation of existing hospitalization for ≥ 24 hours<br>4) A persistent or significant incapacity or substantial disruption of the ability to conduct normal life functions<br>5) A congenital anomaly/birth defect.<br>6) Important Medical Events (IME) that may not result in death, be life threatening, or require hospitalization may be considered serious when, based upon medical judgment, they may jeopardize the patient or

subject and may require medical or surgical intervention to prevent one of the outcomes listed in this definition. (FDA, 21 CFR 312.32; ICH E2A and ICH E6).

<u>ALL</u> <u>SERIOUS</u> adverse events that meet the above criteria MUST be immediately reported to the NCI via electronic submission within the timeframes detailed in the table below.

Hospitalization	Grade 1 and Grade 2 Timeframes	Grade 3-5 Timeframes
Resulting in Hospitalization ≥ 24 hrs	10 Calendar Days	24-Hour 5 Calendar Days
Not resulting in Hospitalization ≥ 24 hrs	Not required	

NOTE: Protocol specific exceptions to expedited reporting of serious adverse events are found in the Specific Protocol Exceptions to Expedited Reporting (SPEER) portion of the CAEPR.

<u>Expedited AE reporting timelines are defined as:</u>

- "24-Hour; 5 Calendar Days" - The AE must initially be submitted electronically within 24 hours of learning of the AE, followed by a complete expedited report within 5 calendar days of the initial 24-hour report.
- "10 Calendar Days" - A complete expedited report on the AE must be submitted electronically within 10 calendar days of learning of the AE.

[1]Serious adverse events that occur more than 30 days after the last administration of investigational agent/intervention and have an attribution of possible, probable, or definite require reporting as follows:
Expedited 24-hour notification followed by complete report within 5 calendar days for:

- All Grade 3, 4, and Grade 5 AEs

Expedited 10 calendar day reports for:

- Grade 2 AEs resulting in hospitalization or prolongation of hospitalization

[2]For studies using PET or SPECT IND agents, the AE reporting period is limited to 10 radioactive half-lives, rounded UP to the nearest whole day, after the agent/intervention was last administered. Footnote "1" above applies after this reporting period.

Effective Date: May 5, 2011

10.4 Routine Adverse Event Reporting

All Adverse Events **must** be reported in routine study data submissions. **AEs reported expeditiously through CTEP-AERS must <u>also</u> be reported in routine study data submissions.**

Adverse event data collection and reporting, which are required as part of every clinical trial, are done to ensure the safety of patients enrolled in the studies as well as those who will enroll in future studies using similar agents. AEs are reported in a routine manner at scheduled times during the trial using Medidata Rave. For this trial the Adverse Event CRF is used for routine AE reporting in Rave.

10.5 Pregnancy

Although not an adverse event in and of itself, pregnancy as well as its outcome must be documented via **CTEP-AERS**. In addition, the ***Pregnancy Information Form*** included within the NCI Guidelines for Adverse Event Reporting Requirements must be completed and

submitted to CTEP. Any pregnancy occurring in a patient or patient's partner from the time of consent to 90 days after the last dose of study drug must be reported and then followed for outcome. Newborn infants should be followed until 30 days old. Please see the "NCI Guidelines for Investigators: Adverse Event Reporting Requirements for DCTD (CTEP and CIP) and DCP INDs and IDEs" (at http://ctep.cancer.gov/protocolDevelopment/adverse_effects.htm) for more details on how to report pregnancy and its outcome to CTEP.

10.6 Secondary Malignancy

A *secondary malignancy* is a cancer caused by treatment for a previous malignancy (*e.g.*, treatment with investigational agent/intervention, radiation or chemotherapy). A secondary malignancy is not considered a metastasis of the initial neoplasm.

CTEP requires all secondary malignancies that occur following treatment with an agent under an NCI IND/IDE be reported expeditiously via CTEP-AERS. Three options are available to describe the event:

- Leukemia secondary to oncology chemotherapy (*e.g.*, acute myelocytic leukemia [AML])
- Myelodysplastic syndrome (MDS)
- Treatment-related secondary malignancy

Any malignancy possibly related to cancer treatment (including AML/MDS) should also be reported via the routine reporting mechanisms outlined in each protocol.

10.7 Second Malignancy

A second malignancy is one unrelated to the treatment of a prior malignancy (and is **NOT** a metastasis from the initial malignancy). Second malignancies require **ONLY** routine AE reporting unless otherwise specified.

11. STUDY CALENDAR

Pre-treatment evaluations are to be conducted within 1 week prior to start of protocol therapy. Scans and x-rays must be done ≤4 weeks prior to the start of therapy. Informed consent may be obtained within ≤ 4 weeks prior to start of protocol therapy. Screening ophthalmologic exam and biopsy may be done ≤ 14 days prior to start of protocol therapy. In the event that the patient's condition is deteriorating, laboratory evaluations should be repeated within 48 hours prior to initiation of the next cycle of therapy. All cycle visits and radiologic evaluations are tied to C1D1.

Procedures	Pre-treatment	Cycles 1 - 4				Cycles 5 and Beyond				Off Study[a]
		Day 1	Day 8	Day 15	Day 22	Day 1	Day 8	Day 15	Day 22	
Window		±2 days	±2 days	±2 days	±2 days	±2 days	±2 days	±2 days	±2 days	
Rogaratinib (BAY 1163877)[e]		X--X								
Informed consent	X									
Demographics	X									
Medical history	X									
Concurrent meds	X	X---X								X
Physical exam	X	X		X		X				X
Vital signs	X	X		X		X				X
Weight	X									
Performance status	X									
CBC w/diff, plts	X	X		X		X				X
Comprehensive Chemistry Panel[b]	X	X		X		X				X
ECG (as indicated)	X									
Adverse event evaluation		X---X								X
Tumor measurements	X	Tumor measurements are repeated every _8_ weeks. Documentation (radiologic) must be provided for patients removed from study for progressive disease.								X

Procedures	Pre-treatment	Cycles 1 - 4				Cycles 5 and Beyond				Off Study[a]
		Day 1	Day 8	Day 15	Day 22	Day 1	Day 8	Day 15	Day 22	
Radiologic evaluation	X[f]	Radiologic measurements should be performed every _8_ weeks (±3 days).								X
Pregnancy test[c]	X									
Ophthalmologic examination	X[g]									
Archival Tissue (if available)	X[h]									
Biopsy	X[i]									X[d]
Biomarker Blood Test	X									
PK Blood Test[J]	X	X		X						X

a: Off-study evaluation.

b: Albumin, alkaline phosphatase, total bilirubin, bicarbonate, BUN, calcium, chloride, creatinine, glucose, LDH, phosphorus, potassium, total protein, SGOT [AST], SGPT [ALT], sodium. Conjugated and unconjugated bilirubin will be evaluated if the bilirubin is found to be elevated and if there is a clinical need.

c: Pregnancy test for women of childbearing potential.

d: Optional biopsy at progression.

e: 800 mg PO BID each day of a 28 day cycle (see Appendix E). Rogaratinib (BAY 1163877) must be taken twice a day with 8 oz. water, with or without food. Swallow whole. Do not chew.

f: pre-treatment radiologic evaluations (scans and x-rays) should be done ≤4 weeks prior to the start of protocol therapy.

g: Ophthalmologic examination is recommended for screening. Additional monitoring as described in Section 7.0. May be done ≤ 14 days prior to start of protocol therapy.

h: Archival tissue (if available) should be submitted for all participants on study.

iI: May be done ≤ 14 days prior to start of protocol therapy. Pre-treatment biopsy is mandatory if disease site is amenable to biopsy and low risk for the biopsy procedure. If biopsy not possible, eligibility may be approved after discussion with medical monitor.

j: Pre-dose (at pre-treatment [prior to C1D1], C1D15, C2D1, C2D15, and C3D1) is mandatory and can be shifted to coincide with the physical exam (due to a holiday or inclement weather) and at the time of Progression (optional). Additional trough samples may be collected at the next physical exam after a dose reduction.

12. MEASUREMENT OF EFFECT

12.1 Antitumor Effect – Solid Tumors

For the purposes of this study, patients should be re-evaluated for response every 8 weeks. In addition to a baseline scan, confirmatory scans should also be obtained 8 weeks (not less than 4) weeks following initial documentation of objective response.

Response and progression will be evaluated in this study using the new international criteria proposed by the revised Response Evaluation Criteria in Solid Tumors (RECIST) guideline (version 1.1) [*Eur J Ca* 45:228-247, 2009]. Changes in the largest diameter (unidimensional measurement) of the tumor lesions and the shortest diameter in the case of malignant lymph nodes are used in the RECIST criteria.

12.1.1 Definitions

Evaluable for Toxicity. All patients will be evaluable for toxicity from the time of their first treatment with rogaratinib (BAY 1163877).

Evaluable for Objective Response. Only those patients who have measurable disease present at baseline, have received at least one cycle of therapy, and have had their disease re-evaluated will be considered evaluable for response. These patients will have their response classified according to the definitions stated below. (Note: Patients who exhibit objective disease progression prior to the end of cycle 1 will also be considered evaluable.)

Evaluable Non-Target Disease Response. Patients who have lesions present at baseline that are evaluable but do not meet the definitions of measurable disease, have received at least one cycle of therapy, and have had their disease re-evaluated will be considered evaluable for non-target disease. The response assessment is based on the presence, absence, or unequivocal progression of the lesions.

12.1.2 Disease Parameters

Measurable Disease. Measurable lesions are defined as those that can be accurately measured in at least one dimension (longest diameter to be recorded) as ≥20 mm (≥2 cm) by chest x-ray or as ≥10 mm (≥1 cm) with CT scan, MRI, or calipers by clinical exam. All tumor measurements must be recorded in millimeters (or decimal fractions of centimeters).

Note: Tumor lesions that are situated in a previously irradiated area might or might not be considered measurable.

Malignant Lymph Nodes. To be considered pathologically enlarged and measurable, a lymph node must be ≥15 mm (≥1.5 cm) in short axis when assessed by CT scan (CT scan slice thickness recommended to be no greater than 5 mm [0.5 cm]). At baseline and in follow-up,

only the short axis will be measured and followed.

Non-Measurable Disease. All other lesions (or sites of disease), including small lesions (longest diameter <10 mm [<1 cm] or pathological lymph nodes with ≥10 to <15 mm [≥1 to <1.5 cm] short axis), are considered non-measurable disease. Bone lesions, leptomeningeal disease, ascites, pleural/pericardial effusions, lymphangitis cutis/pulmonitis, inflammatory breast disease, and abdominal masses (not followed by CT or MRI), are considered as non-measurable.

Note: Cystic lesions that meet the criteria for radiographically defined simple cysts should not be considered as malignant lesions (neither measurable nor non-measurable) since they are, by definition, simple cysts.

'Cystic lesions' thought to represent cystic metastases can be considered as measurable lesions, if they meet the definition of measurability described above. However, if non-cystic lesions are present in the same patient, these are preferred for selection as target lesions.

Target Lesions. All measurable lesions up to a maximum of 2 lesions per organ and 5 lesions in total, representative of all involved organs, should be identified as **target lesions** and recorded and measured at baseline. Target lesions should be selected on the basis of their size (lesions with the longest diameter), be representative of all involved organs, but in addition should be those that lend themselves to reproducible repeated measurements. It may be the case that, on occasion, the largest lesion does not lend itself to reproducible measurement in which circumstance the next largest lesion which can be measured reproducibly should be selected. A sum of the diameters (longest for non-nodal lesions, short axis for nodal lesions) for all target lesions will be calculated and reported as the baseline sum diameters. If lymph nodes are to be included in the sum, then only the short axis is added into the sum. The baseline sum diameters will be used as reference to further characterize any objective tumor regression in the measurable dimension of the disease.

Non-Target Lesions. All other lesions (or sites of disease) including any measurable lesions over and above the 5 target lesions should be identified as **non-target lesions** and should also be recorded at baseline. Measurements of these lesions are not required, but the presence, absence, or in rare cases unequivocal progression of each should be noted throughout follow-up.

12.1.3 Methods for Evaluation of Measurable Disease

All measurements should be taken and recorded in metric notation using a ruler or calipers. All baseline evaluations should be performed as closely as possible to the beginning of treatment and never more than 4 weeks before the beginning of the treatment.

The same method of assessment and the same technique should be used to characterize each identified and reported lesion at baseline and during follow-up. Imaging-based evaluation is preferred to evaluation by clinical examination unless the lesion(s) being followed cannot be imaged but are assessable by clinical exam.

Clinical Lesions. Clinical lesions will only be considered measurable when they are superficial (*e.g.*, skin nodules and palpable lymph nodes) and ≥10 mm (≥1 cm) diameter as assessed using calipers (*e.g.*, skin nodules). In the case of skin lesions, documentation by color photography, including a ruler to estimate the size of the lesion, is recommended.

Chest X-Ray. Lesions on chest x-ray are acceptable as measurable lesions when they are clearly defined and surrounded by aerated lung. However, CT is preferable.

Conventional CT and MRI. This guideline has defined measurability of lesions on CT scan based on the assumption that CT slice thickness is 5 mm (0.5 cm) or less. If CT scans have slice thickness greater than 5 mm (0.5 cm), the minimum size for a measurable lesion should be twice the slice thickness. MRI is also acceptable in certain situations (*e.g.* for body scans).

Use of MRI remains a complex issue. MRI has excellent contrast, spatial, and temporal resolution; however, there are many image acquisition variables involved in MRI, which greatly impact image quality, lesion conspicuity, and measurement. Furthermore, the availability of MRI is variable globally. As with CT, if an MRI is performed, the technical specifications of the scanning sequences used should be optimized for the evaluation of the type and site of disease. Furthermore, as with CT, the modality used at follow-up should be the same as was used at baseline and the lesions should be measured/assessed on the same pulse sequence. It is beyond the scope of the RECIST guidelines to prescribe specific MRI pulse sequence parameters for all scanners, body parts, and diseases. Ideally, the same type of scanner should be used and the image acquisition protocol should be followed as closely as possible to prior scans. Body scans should be performed with breath-hold scanning techniques, if possible.

PET-CT. At present, the low dose or attenuation correction CT portion of a combined PET-CT is not always of optimal diagnostic CT quality for use with RECIST measurements. However, if the site can document that the CT performed as part of a PET-CT is of identical diagnostic quality to a diagnostic CT (with IV and oral contrast), then the CT portion of the PET-CT can be used for RECIST measurements and can be used interchangeably with conventional CT in accurately measuring cancer lesions over time. Note, however, that the PET portion of the CT introduces additional data which may bias an investigator if it is not routinely or serially performed.

Ultrasound. Ultrasound is not useful in assessment of lesion size and should not be used as a method of measurement. Ultrasound examinations cannot be reproduced in their entirety for independent review at a later date and, because they are operator dependent, it cannot be guaranteed that the same technique and measurements will be taken from one assessment to the next. If new lesions are identified by ultrasound in the course of the study, confirmation by CT or MRI is advised. If there is concern about radiation exposure at CT, MRI may be used instead of CT in selected instances.

Endoscopy, Laparoscopy. The utilization of these techniques for objective tumor evaluation is not advised. However, such techniques may be useful to confirm complete pathological response when biopsies are obtained or to determine relapse in trials where recurrence following

complete response (CR) or surgical resection is an endpoint.

Tumor Markers. Tumor markers alone cannot be used to assess response. If markers are initially above the upper normal limit, they must normalize for a patient to be considered in complete clinical response. Specific guidelines for both CA-125 response (in recurrent ovarian cancer) and PSA response (in recurrent prostate cancer) have been published [*JNCI* 96:487-488, 2004; *J Clin Oncol* 17, 3461-3467, 1999; *J Clin Oncol* 26:1148-1159, 2008]. In addition, the Gynecologic Cancer Intergroup has developed CA-125 progression criteria which are to be integrated with objective tumor assessment for use in first-line trials in ovarian cancer [*JNCI* 92:1534-1535, 2000].

Cytology, Histology. These techniques can be used to differentiate between partial responses (PR) and complete responses (CR) in rare cases (*e.g.*, residual lesions in tumor types, such as germ cell tumors, where known residual benign tumors can remain).

The cytological confirmation of the neoplastic origin of any effusion that appears or worsens during treatment when the measurable tumor has met criteria for response or stable disease is mandatory to differentiate between response or stable disease (an effusion may be a side effect of the treatment) and progressive disease.

FDG-PET. While FDG-PET response assessments need additional study, it is sometimes reasonable to incorporate the use of FDG-PET scanning to complement CT scanning in assessment of progression (particularly possible 'new' disease). New lesions on the basis of FDG-PET imaging can be identified according to the following algorithm:

a. Negative FDG-PET at baseline, with a positive FDG-PET at follow-up is a sign of PD based on a new lesion.
b. No FDG-PET at baseline and a positive FDG-PET at follow-up: If the positive FDG-PET at follow-up corresponds to a new site of disease confirmed by CT, this is PD. If the positive FDG-PET at follow-up is not confirmed as a new site of disease on CT, additional follow-up CT scans are needed to determine if there is truly progression occurring at that site (if so, the date of PD will be the date of the initial abnormal FDG-PET scan). If the positive FDG-PET at follow-up corresponds to a pre-existing site of disease on CT that is not progressing on the basis of the anatomic images, this is not PD.
c. FDG-PET may be used to upgrade a response to a CR in a manner similar to a biopsy in cases where a residual radiographic abnormality is thought to represent fibrosis or scarring. The use of FDG-PET in this circumstance should be prospectively described in the protocol and supported by disease-specific medical literature for the indication. However, it must be acknowledged that both approaches may lead to false positive CR due to limitations of FDG-PET and biopsy resolution/sensitivity.

Note: A ‘positive’ FDG-PET scan lesion means one which is FDG avid with an uptake greater than twice that of the surrounding tissue on the attenuation corrected image.

12.1.4 Response Criteria

12.1.4.1 Evaluation of Target Lesions

Complete Response (CR): Disappearance of all target lesions. Any pathological lymph nodes (whether target or non-target) must have reduction in short axis to <10 mm (<1 cm).

Partial Response (PR): At least a 30% decrease in the sum of the diameters of target lesions, taking as reference the baseline sum diameters.

Progressive Disease (PD): At least a 20% increase in the sum of the diameters of target lesions, taking as reference the smallest sum on study (this includes the baseline sum if that is the smallest on study). In addition to the relative increase of 20%, the sum must also demonstrate an absolute increase of at least 5 mm (0.5 cm). (Note: the appearance of one or more new lesions is also considered progressions).

Stable Disease (SD): Neither sufficient shrinkage to qualify for PR nor sufficient increase to qualify for PD, taking as reference the smallest sum diameters while on study.

12.1.4.2 Evaluation of Non-Target Lesions

Complete Response (CR): Disappearance of all non-target lesions and normalization of tumor marker level. All lymph nodes must be non-pathological in size (<10 mm [<1 cm] short axis).

Note: If tumor markers are initially above the upper normal limit, they must normalize for a patient to be considered in complete clinical response.

Non-CR/Non-PD: Persistence of one or more non-target lesion(s) and/or maintenance of tumor marker level above the normal limits.

Progressive Disease (PD): Appearance of one or more new lesions and/or *unequivocal progression* of existing non-target lesions. *Unequivocal progression* should not normally trump target lesion status. It must be representative of overall disease status change, not a single lesion increase.

Although a clear progression of "non-target" lesions only is exceptional, the opinion of the treating physician should prevail in such circumstances, and the progression status should be confirmed at a later time by the review panel (or Principal Investigator).

12.1.4.3 Evaluation of Best Overall Response

The best overall response is the best response recorded from the start of the treatment until disease progression/recurrence (taking as reference for progressive disease the smallest measurements recorded since the treatment started). The patient's best response assignment will depend on the achievement of both measurement and confirmation criteria.

For Patients with Measurable Disease (*i.e.*, Target Disease)

Target Lesions	Non-Target Lesions	New Lesions	Overall Response	Best Overall Response when Confirmation is Required*
CR	CR	No	CR	≥4 wks. Confirmation**
CR	Non-CR/Non-PD	No	PR	≥4 wks. Confirmation**
CR	Not evaluated	No	PR	
PR	Non-CR/Non-PD/not evaluated	No	PR	
SD	Non-CR/Non-PD/not evaluated	No	SD	Documented at least once ≥4 wks. from baseline**
PD	Any	Yes or No	PD	no prior SD, PR or CR
Any	PD	Yes or No	PD	
Any	Any	Yes	PD	

* See RECIST 1.1 manuscript for further details on what is evidence of a new lesion.
** Only for non-randomized trials with response as primary endpoint.

Note: Patients with a global deterioration of health status requiring discontinuation of treatment without objective evidence of disease progression at that time should be reported as "*symptomatic deterioration.*" Every effort should be made to document the objective progression even after discontinuation of treatment.

For Patients with Non-Measurable Disease (*i.e.*, Non-Target Disease)

Non-Target Lesions	New Lesions	Overall Response
CR	No	CR
Non-CR/non-PD	No	Non-CR/non-PD*
Not all evaluated	No	not evaluated
Unequivocal PD	Yes or No	PD
Any	Yes	PD

* 'Non-CR/non-PD' is preferred over 'stable disease' for non-target disease since SD is increasingly used as an endpoint for assessment of efficacy in some trials so to assign this category when no lesions can be measured is not advised

12.1.5 Duration of Response

Duration of overall response: The duration of overall response is measured from the time measurement criteria are met for CR or PR (whichever is first recorded) until the first date that recurrent or progressive disease is objectively documented (taking as reference for progressive disease the smallest measurements recorded since the treatment started).

The duration of overall CR is measured from the time measurement criteria are first met for CR until the first date that progressive disease is objectively documented.

Duration of stable disease: Stable disease is measured from the start of the treatment until the criteria for progression are met, taking as reference the smallest measurements recorded since the treatment started, including the baseline measurements.

12.1.6 Progression-Free Survival

PFS is defined as the duration of time from start of treatment to time of progression or death, whichever occurs first.

12.1.7 Response Review

The PI or designee will review all objective responses.

13. STUDY OVERSIGHT AND DATA REPORTING / REGULATORY REQUIREMENTS

Adverse event lists, guidelines, and instructions for AE reporting can be found in Section 10 (Adverse Events: List and Reporting Requirements).

13.1 Study Oversight

This protocol is monitored at several levels, as described in this section. The Protocol Principal Investigator is responsible for monitoring the conduct and progress of the clinical trial, including the ongoing review of accrual, patient-specific clinical and laboratory data, and routine and serious adverse events; reporting of expedited adverse events; and accumulation of reported adverse events from other trials testing the same drug(s). The Protocol Principal Investigator and statistician have access to the data at all times through the CTMS web-based reporting portal.

All Study Investigators at participating sites who register/enroll patients on a given protocol are responsible for timely submission of data via Medidata Rave and timely reporting of adverse events for that particular study. This includes timely review of data collected on the electronic CRFs submitted via Medidata Rave.

All studies are also reviewed in accordance with the enrolling institution's data safety monitoring plan.

13.2 Data Reporting

Medidata Rave is a clinical data management system being used for data collection for this trial/study. Access to the trial in Rave is controlled through the CTEP-IAM system and role assignments.

Requirements to access Rave via iMedidata:

- A valid account, and
- Assigned a Rave role on the LPO or PO roster at the enrolling site of: Rave CRA, Rave Read Only, Rave CRA (LabAdmin), Rave SLA, or Rave Investigator.
- Rave role requirements:
    - Rave CRA or Rave CRA (Lab Admin) role, must have a minimum of an Associate Plus (AP) registration type,
    - Rave Investigator role, must be registered as a Non-Physician Investigator (NPIVR) or Investigator (IVR), and
    - Rave Read Only role, site staff must have at a minimum an Associates (A) registration type.
- Refer to https://ctep.cancer.gov/investigatorResources/default.htm for registration types and documentation required.

If the study has a DTL, individuals requiring write access to Rave must also be assigned the appropriate Rave tasks on the DTL.

Upon initial site registration approval for the study in Regulatory Support System (RSS), all persons with Rave roles assigned on the appropriate roster will be sent a study invitation e-mail from iMedidata. To accept the invitation, site staff must log in to the Select Login (https://login.imedidata.com/selectlogin) using their CTEP-IAM username and password, and click on the *accept* link in the upper right-corner of the iMedidata page. Site staff will not be able to access the study in Rave until all required Medidata and study specific trainings are completed. Trainings will be in the form of electronic learnings (eLearnings), and can be accessed by clicking on the link in the upper right pane of the iMedidata screen. If an eLearning is required and has not yet been taken, the link to the eLearning will appear under the study name in iMedidata instead of the *Rave EDC* link; once the successful completion of the eLearning has been recorded, access to the study in Rave will be granted, and a *Rave EDC* link will display under the study name.

Site staff that have not previously activated their iMedidata/Rave account at the time of initial site registration approval for the study in RSS will receive a separate invitation from iMedidata to activate their account. Account activation instructions are located on the CTSU website in the Data Management section under the Rave resource materials (Medidata Account Activation and Study Invitation Acceptance). Additional information on iMedidata/Rave is available on the CTSU members' website in the Data Management > Rave section or by contacting the CTSU Help Desk.

13.2.1 Method

This study will be monitored by the Clinical Trials Monitoring Service (CTMS). Data will be submitted to CTMS at least once every two weeks via Medidata Rave (or other modality if approved by CTEP). Information on CTMS reporting is available at: http://www.theradex.com/clinicalTechnologies/?National-Cancer-Institute-NCI-11. On-site

audits will be conducted on an 18-36 month basis as part of routine cancer center site visits. More frequent audits may be conducted if warranted by accrual or due to concerns regarding data quality or timely submission. For CTMS monitored studies, after users have activated their accounts, please contact the Theradex Help Desk for additional support with Rave and completion of CRFs.

13.2.2 Responsibility for Data Submission

For ETCTN trials, it is the responsibility of the PI(s) at the site to ensure that all investigators at the ETCTN Sites understand the procedures for data submission for each ETCTN protocol and that protocol specified data are submitted accurately and in a timely manner to the CTMS via the electronic data capture system, Medidata Rave.

Data are to be submitted via Medidata Rave to CTMS on a real-time basis, but no less than once every 2 weeks. The timeliness of data submissions and timeliness in resolving data queries will be tracked by CTMS. Metrics for timeliness will be followed and assessed on a quarterly basis. For the purpose of Institutional Performance Monitoring, data will be considered delinquent if it is greater than 4 weeks past due.

Data from Medidata Rave and CTEP-AERS is reviewed by the CTMS on an ongoing basis as data is received. Queries will be issued by CTMS directly within Rave. The queries will appear on the Task Summary Tab within Rave for the CRA at the ETCTN to resolve. Monthly web-based reports are posted for review by the Drug Monitors in the IDB, CTEP. Onsite audits will be conducted by the CTMS to ensure compliance with regulatory requirements, GCP, and NCI policies and procedures with the overarching goal of ensuring the integrity of data generated from NCI-sponsored clinical trials, as described in the ETCTN Program Guidelines, which may be found on the CTEP (http://ctep.cancer.gov/protocolDevelopment/electronic_applications/adverse_events.htm) and CTSU websites.

CTMS will utilize a core set of eCRFs that are Cancer Data Standards Registry and Repository (caDSR) compliant (http://cbiit.nci.nih.gov/ncip/biomedical-informatics-resources/interoperability-and-semantics/metadata-and-models). Customized eCRFs will be included when appropriate to meet unique study requirements. The PI is encouraged to review the eCRFs, working closely with CTMS to ensure prospectively that all required items are appropriately captured in the eCRFs prior to study activation. CTMS will prepare the eCRFs with built-in edit checks to the extent possible to promote data integrity.

CDUS data submissions for ETCTN trials activated after March 1, 2014, will be carried out by the CTMS contractor, Theradex. CDUS submissions are performed by Theradex on a monthly basis. The trial's lead institution is responsible for timely submission to CTMS via Rave, as above.

Further information on data submission procedures can be found in the ETCTN Program Guidelines

(http://ctep.cancer.gov/protocolDevelopment/electronic_applications/adverse_events.htm).

13.3 Data Quality Portal

The Data Quality Portal (DQP) provides a central location for site staff to manage unanswered queries and form delinquencies, monitor data quality and timeliness, generate reports, and review metrics.

The DQP is located on the CTSU members' website under Data Management. The Rave Home section displays a table providing summary counts of Total Delinquencies and Total Queries. DQP Queries, DQP Delinquent Forms, and the DQP Reports modules are available to access details and reports of unanswered queries, delinquent forms, and timeliness reports. Review the DQP modules on a regular basis to manage specified queries and delinquent forms.

The DQP is accessible by site staff that are rostered to a site and have access to the CTSU website. Staff that have Rave study access can access the Rave study data using a direct link on the DQP.

To learn more about DQP use and access, click on the Help icon displayed on the Rave Home, DQP Queries, and DQP Delinquent Forms modules.

Note: Some Rave protocols may not have delinquent form details or reports specified on the DQP. A protocol must have the Calendar functionality implemented in Rave by the Lead Protocol Organization (LPO) for delinquent form details and reports to be available on the DQP. Site staff should contact the LPO Data Manager for their protocol regarding questions about Rave Calendaring functionality.

13.4 CTEP Multicenter Guidelines

Not applicable.

13.5 Collaborative Agreements Language

The agent(s) supplied by CTEP, DCTD, NCI used in this protocol is/are provided to the NCI under a Collaborative Agreement (CRADA, CTA, CSA) between the Pharmaceutical Company(ies) (hereinafter referred to as "Collaborator(s)") and the NCI Division of Cancer Treatment and Diagnosis. Therefore, the following obligations/guidelines, in addition to the provisions in the "Intellectual Property Option to Collaborator" (http://ctep.cancer.gov/industryCollaborations2/intellectual_property.htm) contained within the terms of award, apply to the use of the Agent(s) in this study:

1. Agent(s) may not be used for any purpose outside the scope of this protocol, nor can Agent(s) be transferred or licensed to any party not participating in the clinical study. Collaborator(s) data for Agent(s) are confidential and proprietary to Collaborator(s) and shall

be maintained as such by the investigators. The protocol documents for studies utilizing Agents contain confidential information and should not be shared or distributed without the permission of the NCI. If a copy of this protocol is requested by a patient or patient's family member participating on the study, the individual should sign a confidentiality agreement. A suitable model agreement can be downloaded from: http://ctep.cancer.gov.

2. For a clinical protocol where there is an investigational Agent used in combination with (an)other Agent(s), each the subject of different Collaborative Agreements, the access to and use of data by each Collaborator shall be as follows (data pertaining to such combination use shall hereinafter be referred to as "Multi-Party Data"):

    a. NCI will provide all Collaborators with prior written notice regarding the existence and nature of any agreements governing their collaboration with NCI, the design of the proposed combination protocol, and the existence of any obligations that would tend to restrict NCI's participation in the proposed combination protocol.

    b. Each Collaborator shall agree to permit use of the Multi-Party Data from the clinical trial by any other Collaborator solely to the extent necessary to allow said other Collaborator to develop, obtain regulatory approval or commercialize its own Agent.

    c. Any Collaborator having the right to use the Multi-Party Data from these trials must agree in writing prior to the commencement of the trials that it will use the Multi-Party Data solely for development, regulatory approval, and commercialization of its own Agent.

3. Clinical Trial Data and Results and Raw Data developed under a Collaborative Agreement will be made available to Collaborator(s), the NCI, and the FDA, as appropriate and unless additional disclosure is required by law or court order as described in the IP Option to Collaborator (http://ctep.cancer.gov/industryCollaborations2/intellectual_property.htm). Additionally, all Clinical Data and Results and Raw Data will be collected, used and disclosed consistent with all applicable federal statutes and regulations for the protection of human subjects, including, if applicable, the *Standards for Privacy of Individually Identifiable Health Information* set forth in 45 C.F.R. Part 164.

4. When a Collaborator wishes to initiate a data request, the request should first be sent to the NCI, who will then notify the appropriate investigators (Group Chair for Cooperative Group studies, or PI for other studies) of Collaborator's wish to contact them.

5. Any data provided to Collaborator(s) for Phase 3 studies must be in accordance with the guidelines and policies of the responsible Data Monitoring Committee (DMC), if there is a DMC for this clinical trial.

6. Any manuscripts reporting the results of this clinical trial must be provided to CTEP by the Group office for Cooperative Group studies or by the principal investigator for non-Cooperative Group studies for immediate delivery to Collaborator(s) for advisory review and

comment prior to submission for publication. Collaborator(s) will have 30 days from the date of receipt for review. Collaborator shall have the right to request that publication be delayed for up to an additional 30 days in order to ensure that Collaborator's confidential and proprietary data, in addition to Collaborator(s)'s intellectual property rights, are protected. Copies of abstracts must be provided to CTEP for forwarding to Collaborator(s) for courtesy review as soon as possible and preferably at least three (3) days prior to submission, but in any case, prior to presentation at the meeting or publication in the proceedings. Press releases and other media presentations must also be forwarded to CTEP prior to release. Copies of any manuscript, abstract and/or press release/ media presentation should be sent to CTEP.

The Regulatory Affairs Branch will then distribute them to Collaborator(s). No publication, manuscript or other form of public disclosure shall contain any of Collaborator's confidential/ proprietary information.

13.6 Genomic Data Sharing Plan

Not applicable.

13.7 Incidental/Secondary Findings Disclosure Procedure

Not applicable.

14. REFERENCES

Rogaratinib Investigator's Brochure, 2019, BAY 1163877 (Rogaratinib), Version 11.0, 10 December 2019, Bayer AG.

Boikos, S.A., A.S. Pappo, J.K. Killian, *et al*. (2016). Molecular Subtypes of KIT/PDGFRA Wild-Type Gastrointestinal Stromal Tumors: A Report From the National Institutes of Health Gastrointestinal Stromal Tumor Clinic. *JAMA Oncol*. 2(7):922-928.

Brennan, M.F. (2013). Lessons learned from the study of soft tissue sarcoma. *Int J Surg*. 11 Suppl 1:S8-10.

Crose, L.E., K.T. Etheridge, C. Chen, *et al*. (2012). FGFR4 blockade exerts distinct antitumorigenic effects in human embryonal versus alveolar rhabdomyosarcoma. *Clin Cancer Res*. 18(14):3780-3790.

Flavahan, W.A., Y. Drier, S.E. Johnstone, *et al*. (2019). Altered chromosomal topology drives oncogenic programs in SDH-deficient GIST. *Nature*. Epub 2019/10/17.

Hanes, R., I. Grad, S. Lorenz, *et al*. (2016). Preclinical evaluation of potential therapeutic targets in dedifferentiated liposarcoma. *Oncotarget*. 7(34):54583-54595.

Holme, H., A. Gulati, R. Brough, *et al*. (2018). Chemosensitivity profiling of osteosarcoma tumour cell lines identifies a model of BRCAness. *Sci Rep*. 8(1):10614.

Italiano, A., S. Mathoulin-Pelissier, A.L. Cesne, *et al*. (2011). Trends in survival for patients with metastatic soft-tissue sarcoma. *Cancer*. 117(5):1049-1054.

Janeway, K.A., K.H. Albritton, A.D. Van Den Abbeele, *et al*. (2009). Sunitinib treatment in pediatric patients with advanced GIST following failure of imatinib. *Pediatr Blood Cancer*. 52(7):767-71.

Janeway, K.A., S.Y. Kim, M. Lodish, *et al*. (2011). Defects in succinate dehydrogenase in gastrointestinal stromal tumors lacking KIT and PDGFRA mutations. *Proc Natl Acad Sci U S A*. 108(1):314-318.

Kelly, C.M., A.N. Shoushtari, L.X. Qin, *et al*. (2019). A phase Ib study of BGJ398, a pan-FGFR kinase inhibitor in combination with imatinib in patients with advanced gastrointestinal stromal tumor. *Invest New Drugs*. 37(2):282-290.

Li, F., H. Huynh, X. Li, *et al*. (2015). FGFR-Mediated Reactivation of MAPK Signaling Attenuates Antitumor Effects of Imatinib in Gastrointestinal Stromal Tumors. *Cancer Discov*. 5(4):438-451.

Pappo, A.S., K.A. Janeway. (2009). Pediatric gastrointestinal stromal tumors. *Hematol Oncol*

Clin North Am. 23(1):15-34.

Patwardhan, P.P., E. Musi, G.K. Schwartz. (2018). Preclinical Evaluation of Nintedanib, a Triple Angiokinase Inhibitor, in Soft-tissue Sarcoma: Potential Therapeutic Implication for Synovial Sarcoma. *Mol Cancer Ther*. 17(11):2329-2340.

Siegel, R., J. Ma, Z. Zou, *et al*. (2014). Cancer Statistics, 2014. *CA Cancer J Clin*. 64(1):9-29.

Taylor, J.G., A.T. Cheuk, P.S. Tsang, *et al*. (2009). Identification of FGFR4-activating mutations in human rhabdomyosarcomas that promote metastasis in xenotransplanted models. *J Clin Invest*. 119(11):3395-3407.

van der Graaf, W.T., J.Y. Blay, S.P. Chawla, *et al*. (2012). Pazopanib for metastatic soft-tissue sarcoma (PALETTE): a randomised, double-blind, placebo-controlled phase 3 trial. *Lancet*. 2;379(9829):1879-1886.

Wachtel, M., J. Rakic, M. Okoniewski, *et al*. (2014). FGFR4 signaling couples to Bim and not Bmf to discriminate subsets of alveolar rhabdomyosarcoma cells. *Int J Cancer*. 135(7):1543-1552.

APPENDIX A PERFORMANCE STATUS CRITERIA

**ECOG Performance Status Scale**		**Karnofsky Performance Scale**	
Grade	Descriptions	Percent	Description
0	Normal activity. Fully active, able to carry on all pre-disease performance without restriction.	100	Normal, no complaints, no evidence of disease.
		90	Able to carry on normal activity; minor signs or symptoms of disease.
1	Symptoms, but ambulatory. Restricted in physically strenuous activity, but ambulatory and able to carry out work of a light or sedentary nature (*e.g.*, light housework, office work).	80	Normal activity with effort; some signs or symptoms of disease.
		70	Cares for self, unable to carry on normal activity or to do active work.
2	In bed <50% of the time. Ambulatory and capable of all self-care, but unable to carry out any work activities. Up and about more than 50% of waking hours.	60	Requires occasional assistance, but is able to care for most of his/her needs.
		50	Requires considerable assistance and frequent medical care.
3	In bed >50% of the time. Capable of only limited self-care, confined to bed or chair more than 50% of waking hours.	40	Disabled, requires special care and assistance.
		30	Severely disabled, hospitalization indicated. Death not imminent.
4	100% bedridden. Completely disabled. Cannot carry on any self-care. Totally confined to bed or chair.	20	Very sick, hospitalization indicated. Death not imminent.
		10	Moribund, fatal processes progressing rapidly.
5	Dead.	0	Dead.

APPENDIX B FORMULA TO ESTIMATE RENAL FUNCTION USING SERUM CREATININE

Formulas to estimate renal function using serum creatinine provided by the NCI's Investigational Drug Steering Committee (IDSC) Pharmacological Task Force in table below.

1. Estimated glomerular filtration rate (eGFR) using the Chronic Kidney Disease Epidemiology Collaboration (CKD-EPI) (Levey *et al.*, 2009).

Formulae:

**Race and Sex**	**Serum Creatinine (SCr), *µmol/L (mg/dL)***	**Equation**
**Black**		
Female	≤62 (≤0.7)	$GFR = 166 \times (SCr/0.7)^{-0.329} \times (0.993)^{Age}$
	>62 (>0.7)	$GFR = 166 \times (SCr/0.7)^{-1.209} \times (0.993)^{Age}$
Male	≤80 (≤0.9)	$GFR = 163 \times (SCr/0.9)^{-0.411} \times (0.993)^{Age}$
	>80 (>0.9)	$GFR = 163 \times (SCr/0.9)^{-1.209} \times (0.993)^{Age}$
**White or other**		
Female	≤62 (≤0.7)	$GFR = 144 \times (SCr/0.7)^{-0.329} \times (0.993)^{Age}$
	>62 (>0.7)	$GFR = 144 \times (SCr/0.7)^{-1.209} \times (0.993)^{Age}$
Male	≤80 (≤0.9)	$GFR = 141 \times (SCr/0.9)^{-0.411} \times (0.993)^{Age}$
	>80 (>0.9)	$GFR = 141 \times (SCr/0.9)^{-1.209} \times (0.993)^{Age}$

SCr in mg/dL; Output is in mL/min/1.73 m^2 and needs no further conversions.

References

1. Levey, A.S., L.A. Stevens, C.H. Schmid, *et al*. (2009). A new equation to estimate glomerular filtration rate. *Ann Inter Med*. 150:604-612.
2. Levey, A.S., J. Coresh, T. Greene, *et al*. (2006). Using standardized serum creatinine values in the modification of diet in renal disease study equation for estimating glomerular filtration rate. *Ann Intern Med*. 145:247-254.
3. Cockcroft, D.W. and M.H. Gault. (1976). Prediction of creatinine clearance from serum creatinine. *Nephron*. 16:31-41.

APPENDIX C PATIENT DRUG INTERACTIONS HANDOUT AND WALLET CARD

Information for Patients, Their Caregivers and Non-Study Healthcare Team on Possible Interactions with Other Drugs and Herbal Supplements

**Patient Name:**		**Diagnosis:**		**Trial #:**	10411
**Study Doctor:**		**Study Doctor Phone #:**		**Study Drug(s):**	Rogaratinib (BAY 1163877)

Please show this paper to all your healthcare providers (doctors, physician assistants, nurse practitioners, pharmacists), and tell them you are taking part in a clinical trial sponsored by the National Cancer Institute.

These are the things that your healthcare providers need to know:
Rogaratinib (BAY 1163877) interacts with certain specific enzyme in your liver or other tissues like the gut and certain transport proteins that help move drugs in and out of the cell.

	Explanation
CYP isoenzymes	The enzyme in question is **CYP3A4**. Rogaratinib (BAY 1163877) is broken down by this enzyme and may be affected by other drugs that inhibit or induce this enzyme. Rogaratinib (BAY 1163877) can also inhibit this enzyme and may affect sensitive drugs or drugs with a narrow therapeutic window that are broken down by this enzyme.
Transport proteins	The proteins in question are P-gp, BCRP, MATE1, and MATE2K. Rogaratinib (BAY 1163877) is moved in and out of cells/organs by P-gp and may be affected by other drugs that inhibit or induce this transport protein. Rogaratinib (BAY 1163877) inhibits P-gp, BCRP, MATE1, and MATE2K and may be affect sensitive drugs or drugs with narrow therapeutic window that are moved in and out of cells/organs by this transport protein.

These are the things that you need to know:
The study drug rogaratinib (BAY 1163877), may interact with other drugs which can cause side effects. For this reason, it is very important to tell your doctors about all your medicines, including: (a) medicines you are taking before this clinical trial, (b) medicines you start or stop taking during this study, (c) medicines you buy without a prescription (over-the-counter remedy), (d) herbals or supplements (e.g. St. John's Wort). It is helpful to bring your medication bottles or an updated medication list with you.

Before you enroll onto the clinical trial, your study doctor will work with your regular health care providers to review any medicines and herbal supplements that are considered strong inhibitors and inducers of CYP3A4 and P-gp, and sensitive substrates/narrow therapeutic index drugs of CYP3A4, P-gp, BCRP, MATE1, and MATE2K.

- Please be very careful! Over-the-counter drugs (including herbal supplements) may contain ingredients that could interact with your study drug. Speak to your doctors or pharmacist to determine if there could be any side effects.

- No grapefruit juice, Seville oranges, or grapefruit can be consumed while on rogaratinib (BAY 1163877)

- Make sure your doctor knows to avoid certain prescription medications.
- Your regular health care provider should check a frequently updated medical reference or call your study doctor before prescribing any new medicine or discontinuing any medicine.

Version *JUN/2020*

(Next page: Patient Drug Interaction Wallet Card)

PATIENT DRUG INTERACTION WALLET CARD

NIH NATIONAL CANCER INSTITUTE

EMERGENCY INFORMATION

Show this card to all of your healthcare providers. Keep it with you in case you go to the emergency room.

Patient Name:

Diagnosis:

Study Doctor:

Study Doctor Phone #:

NCI Trial #: 10411

Study Drug(S): Rogaratinib (BAY 1163877)

For more information: 1-800-4-CANCER
cancer.gov | clinicaltrials.gov

NIH NATIONAL CANCER INSTITUTE

Tell your doctors **before** you **start** or **stop** any medicines.

Check with your doctor or pharmacist if you need to use an over-the-counter medicine or herbal supplement!

Use caution and avoid the following drugs if possible:

- No grapefruit juice, Seville oranges, or grapefruit can be consumed while on rogaratinib (BAY 1163877)

For more information: 1-800-4-CANCER
cancer.gov | clinicaltrials.gov

NIH NATIONAL CANCER INSTITUTE

NIH NATIONAL CANCER INSTITUTE

DRUG INTERACTIONS

Carry this card with you at all times

Rogaratinib (BAY 1163877) interacts with specific liver enzymes called CYP3A4, and transport proteins P-gp, BCRP, MATE1, and MATE2K and must be used very carefully with other medicines.

Your healthcare providers should be aware of any medicines that are strong inhibitors and inducers of CYP3A4, and sensitive substrates/narrow therapeutic index drugs of CYP3A4, P-gp, BCRP, MATE1, and MATE2K.

Avoid concomitant administration with strong inhibitors and inducers of CYP3A4, and sensitive substrates/narrow therapeutic index drugs of CYP3A4, P-gp, BCRP, MATE1, and MATE2K.

Use caution with strong inhibitors and inducers of P-gp.

Before prescribing new medicines, your health care provider should check a **frequently-updated medical reference** for a **list of drugs to avoid** or contact your study doctor.

Version JUN/2020

For more information: 1-800-4-CANCER
cancer.gov | clinicaltrials.gov

For more information: 1-800-4-CANCER
cancer.gov | clinicaltrials.gov

APPENDIX D TISSUE BIOPSY VERIFICATION

A copy of the diagnostic pathology report must be shipped with all tissue specimens sent to the EET Biobank.

If the *corresponding* pathology report is not available for the biopsy, then a copy of the radiology report or operative report from the biopsy procedure <u>and</u> the diagnostic pathology report must be sent to the EET Biobank. A completed copy of this appendix (i.e., Tissue Biopsy Verification) must also be submitted to the EET Biobank.

Note: If this information is not provided with the biopsy specimen, then it will not be accepted by the EET Biobank.

Please have the Clinician* responsible for signing out this patient's case complete the following:

ETCTN Universal Patient ID: ____

ETCTN Patient Study ID: ____

Date of Procedure (mm/dd/yyyy): ____

Tissue Type (circle one): Primary Metastatic

Time point (circle one): Pre-Treatment Progression

Site Tissue Taken From: ____

Diagnosis: ____

I agree that this tissue may be released for research purposes only and that the release of this tissue will not have any impact on the patient's care.

________________________________ ____________________
Clinician Signature Date

____
Clinician Printed Name

*Note: For the purposes of this form, Clinician could include the Nurse Practitioner, Registered Nurse, Pathologist, Radiologist, Interventional Radiologist, Surgeon, Oncologist, Internist, or other medical professional responsible for the patient's care.

Version: 1
Effective Date: 9/2019

APPENDIX E PATIENT'S MEDICATION DIARY

CTEP-assigned Protocol #__10411_
Local Protocol # ___________________

PATIENT'S MEDICATION DIARY

Today's date ________________________ **Agent** **Rogaratinib (BAY 1163877)** _________

Patient Name________________________(*initials acceptable*) **Patient Study ID** ___________________________________

INSTRUCTIONS TO THE PATIENT:

1. Complete one form for each month.
2. You will take ___ tablets each day, ___ in the morning and ___ in the evening. You should take the tablets with 8 oz. water, with or without food. Swallow whole. Do not chew.
3. If you miss a dose, you can take the dose if it is within 3 hours of the time you usually take the dose. If it is has been more than 3 hours since the usual time, skip the dose and take your next dose at the usual time. Vomited doses should not be replaced.
4. Record the date, the number of tablets you took, and when you took them.
5. If you have any comments or notice any side effects, please record them in the Comments column.
6. Please return the forms to your physician when you go for your next appointment.

Day	Date	Time of morning dose	# of tablets taken	Time of evening dose	# of tablets taken	Comments
1						
2						
3						
4						
5						
6						
7						
8						
9						
10						
11						
12						
13						
14						
15						
16						
17						
18						
19						
20						
21						
22						
23						
24						
25						
26						
27						
28						

Patient's Signature____

Physician's Office will complete this section:

1. Date patient started protocol treatment____

2. Date patient was removed from study____
3. Patient's planned total daily dose____
4. Total number of tablets taken this month ____
5. Physician/Nurse/Data Manager's Signature ____

APPENDIX F PATIENT'S PHARMACOKINETIC WORKSHEET

CTEP-assigned Protocol #__10411_

Local Protocol # ___________________

Study Sample Collection Log

Patient Initials (First_Middle_Last):	Patient Study ID:	Site Name:

Pharmacokinetic (PK) Sample Collection

RogaratinibPharmacokinetic (PK) Sample collection

At each time point, ~**4 mL** of peripheral blood will be collected in a **purple-topped (EDTA)**, mix by inversion, and place sample immediately on ice after collection; samples must be processed within 30 minutes. After sample processing, store plasma samples at -70°C or below until shipment or transfer to Johns Hopkins Analytical Pharmacology Core Laboratory. *See Section 5.5.2.2. of the protocol for more specific processing instructions and shipping instructions. At the time of sample transfer, the dosing information must be transferred also.*

Rogaratinib				
**Protocol Sample and Time Point**	**Actual Sample Collection Date**	**Actual Time Sample Collected (24 hr clock)**	**Sample Freeze Time (24 hr clock)**	**Comments**
Pre-Study/Prior to Cycle 1 Day 1 (first Rogaratinib dose): Date/time of Rogaratinib administered: __________________________ RogaratinibDose (mg):___________				
pre Rogaratinib				
Cycle 1 Day 15: Date/time of Rogaratinib administered: __________________________ Rogaratinib Dose (mg):__________Compliance: Did the patient take all doses through Cycle 1 Day 14? Y or N				
pre Rogaratinib				
Cycle 2 Day 1: Date/time of Rogaratinib administered: __________________________ Rogaratinib Dose (mg):__________Compliance: Did the patient take all during Cycle 1? Y or N				
pre Rogaratinib				
Cycle 2 Day 15: Date/time of Rogaratinib administered: __________________________ Rogaratinib Dose (mg):__________Compliance: Did the patient take all doses through Cycle 2 Day 14? Y or N				
pre Rogaratinib				
Cycle 3 Day 1: Date/time of Rogaratinib administered: __________________________ Rogaratinib Dose (mg):__________Compliance: Did the patient take all during Cycle 2? Y or N				
pre Rogaratinib				
Progression: Last Date/time of Rogaratinib administered: __________________________ Rogaratinib Dose (mg):__________Compliance: Was the patient compliant for the last cycle? Y or N				
pre Rogaratinib				
**Pre-treatment Samples at Clinic Visits after a Dose Reduction: Date/time of Rogaratinib administered:** __________________________ **Current Rogaratinib Dose (mg):**__________ **Date of Dose Reduction** ____________				
**Dose Reduction** pre Rogaratinib				
