# Supplementary material for: Fibroblast growth factor receptor inhibition for succinate dehydrogenase-deficient gastrointestinal stromal tumors: a phase 2 trial
Source: Nat Med. 2026 May 26;32(6):2191–200. doi: 10.1038/s41591-026-04376-9 (PMC13279270; doi:10.1038/s41591-026-04376-9)
Supplement: Supplementary file 2 — Reporting Summary [file 41591_2026_4376_MOESM2_ESM.pdf]

nature portfolio

Corresponding author(s): Suzanne George, MD
Bradley Bernstein, MD,PhD

Last updated by author(s): Mar 16, 2026

Reporting Summary

Nature Portfolio wishes to improve the reproducibility of the work that we publish. This form provides structure for consistency and transparency in reporting. For further information on Nature Portfolio policies, see our Editorial Policies and the Editorial Policy Checklist.

Statistics

For all statistical analyses, confirm that the following items are present in the figure legend, table legend, main text, or Methods section.

n/a	Confirmed	
☐	☒	The exact sample size (*n*) for each experimental group/condition, given as a discrete number and unit of measurement
☐	☒	A statement on whether measurements were taken from distinct samples or whether the same sample was measured repeatedly
☐	☒	The statistical test(s) used AND whether they are one- or two-sided<br>*Only common tests should be described solely by name; describe more complex techniques in the Methods section.*
☒	☐	A description of all covariates tested
☒	☐	A description of any assumptions or corrections, such as tests of normality and adjustment for multiple comparisons
☐	☒	A full description of the statistical parameters including central tendency (e.g. means) or other basic estimates (e.g. regression coefficient) AND variation (e.g. standard deviation) or associated estimates of uncertainty (e.g. confidence intervals)
☐	☒	For null hypothesis testing, the test statistic (e.g. *F*, *t*, *r*) with confidence intervals, effect sizes, degrees of freedom and *P* value noted<br>*Give P values as exact values whenever suitable.*
☒	☐	For Bayesian analysis, information on the choice of priors and Markov chain Monte Carlo settings
☒	☐	For hierarchical and complex designs, identification of the appropriate level for tests and full reporting of outcomes
☒	☐	Estimates of effect sizes (e.g. Cohen's *d*, Pearson's *r*), indicating how they were calculated

Our web collection on statistics for biologists contains articles on many of the points above.

Software and code

Policy information about availability of computer code

Data collection	Clinical data: iMedidata platform (imedidata.com), SAS On Demand<br>Correlative data: GraphPad PRISM 10.3.1 (analyze and generate PDX figures), MuTect2 software (mutation identification), STAR (gene expression analysis), RSEM and DESeq (normalized counts), MoCha RNAseq bioinformatics pipeline, Studylog software version 4.6.1.3.
Data analysis	Clinical data: Data analysis was performed using R version 4.5.0<br>Correlative data: BWA (sequence alignment), Sequenza (copy number calls), WinNonlin (Certara), RStudio 2024.12.1, R package lmerTest version 3.2-0.

For manuscripts utilizing custom algorithms or software that are central to the research but not yet described in published literature, software must be made available to editors and reviewers. We strongly encourage code deposition in a community repository (e.g. GitHub). See the Nature Portfolio guidelines for submitting code & software for further information.

Data

Policy information about availability of data

All manuscripts must include a data availability statement. This statement should provide the following information, where applicable:

- Accession codes, unique identifiers, or web links for publicly available datasets
- A description of any restrictions on data availability
- For clinical datasets or third party data, please ensure that the statement adheres to our policy

The study protocol can be found in the Supplementary Information. Original RNA-seq and whole exome sequencing data generated from tumor specimens for patients on this clinical trial have been deposited in the database of Genotypes and Phenotypes (dbGaP) under accession phs004352. Identifiable patient data cannot be openly available due to confidentiality, but de-identified data requests may be directed to the Corresponding Authors (suzanne_george@dfci.harvard.edu and bradley_bernstein@dfci.harvard.edu). Requests will be reviewed for compliance with confidentiality restrictions with an estimated response time within 3 weeks.

Research involving human participants, their data, or biological material

Policy information about studies with human participants or human data. See also policy information about sex, gender (identity/presentation), and sexual orientation and race, ethnicity and racism.

Reporting on sex and gender	Sex- and gender-based analyses were not planned a priori and the findings do not apply exclusively to one sex or gender. Sex was reported by the study team into the central database. Data were not disaggregated based on sex and gender. Sex of participants (in aggregate) was reported as part of Patient Characteristics.
Reporting on race, ethnicity, or other socially relevant groupings	The study does not report on race, ethnicity, or other socially relevant categorization variables.
Population characteristics	Patients had locally advanced or unresectable succinate-dehydrogenase gastrointestinal stromal tumor. Patients were 18 years or older, with a median age of 52 (range: 22-88), and 54% of patients were female. 67% of patients were previously treated with systemic therapy.<br><br>The study did not categorize humans by race, ethnicity, national or social origin, gender identity, sexual orientation, religion, political or other beliefs, (dis)ability, socio-economic status, or other socially constructed or socially relevant groupings. We report sex, age, and disease status as patient characteristics, but do not categorize based on these characteristics.
Recruitment	Participants were recruited for the study based on their diagnosis, and were either self referred, or referred by their treating physician to the study at one of the study sites. All patients provided written informed consent before enrollment. As the study included participants ages 18 and older, there could be an age-related bias in outcomes. Patients were not paid for participation in the study. As patients were required to have an Eastern Cooperative Oncology Group Performance Status (ECOG) of 0-2 and adequate organ and bone marrow function, the study population may be in better health than some patients with the same diagnosis, which could bias the results of responses to and tolerance of the study drug.
Ethics oversight	The protocol, informed consent and subsequent amendments were reviewed and approved by the Central IRB of the NCI, with additional approvals at each site as required by local requirements.

Note that full information on the approval of the study protocol must also be provided in the manuscript.

Field-specific reporting

Please select the one below that is the best fit for your research. If you are not sure, read the appropriate sections before making your selection.

☒ Life sciences ☐ Behavioural & social sciences ☐ Ecological, evolutionary & environmental sciences

For a reference copy of the document with all sections, see nature.com/documents/nr-reporting-summary-flat.pdf

Life sciences study design

All studies must disclose on these points even when the disclosure is negative.

Sample size	The trial was designed as a Simon two-stage optimal design, targeting at least a 20% improvement in objective response rate, from a historical control rate of 5% to 25% or higher, with parameters including a one-sided type-1 error of 10%, and power of 90%. The two-stage structure required one or more responses in the first 9 patients to proceed to the second stage, serving as an early futility checkpoint to halt the study if the treatment showed no early signs of activity. If this initial threshold was met, enrollment would continue to a total accrual of 24 patients in the second stage. The final efficacy endpoint required responses in 3 or more patients out of the total 24 enrolled patients to declare the trial positive. The actual power achieved by this setting was 90.3%, with an actual type-1 error of 9.3% and a probability of stopping at the 1st stage equal to 63%.
Data exclusions	No data were excluded from the analyses.

Replication	Replication was not feasible for data from this phase 2 trial as the clinical investigations were performed only once.
Randomization	Randomization was not performed in this study.
Blinding	Blinding was not performed in this single-arm study.

Reporting for specific materials, systems and methods

We require information from authors about some types of materials, experimental systems and methods used in many studies. Here, indicate whether each material, system or method listed is relevant to your study. If you are not sure if a list item applies to your research, read the appropriate section before selecting a response.

Materials & experimental systems

n/a	Involved in the study	
☒	☐	Antibodies
☒	☐	Eukaryotic cell lines
☒	☐	Palaeontology and archaeology
☐	☒	Animals and other organisms
☐	☒	Clinical data
☒	☐	Dual use research of concern
☒	☐	Plants

Methods

n/a	Involved in the study	
☒	☐	ChIP-seq
☒	☐	Flow cytometry
☒	☐	MRI-based neuroimaging

Animals and other research organisms

Policy information about studies involving animals; ARRIVE guidelines recommended for reporting animal research, and Sex and Gender in Research

Laboratory animals	For efficacy studies, tumor fragments were implanted into 8-week-old female NOD Cg-Prkdcscid Il2rgtm1Wjl/SzJ (NSG) mice purchased from The Jackson Laboratory (Bar Harbor, ME).
Wild animals	N/A
Reporting on sex	No sex- or gender-based analyses were performed.
Field-collected samples	N/A
Ethics oversight	All animal experiments were conducted at Dana-Farber Cancer Institute with the approval of the Institutional Animal Care and Use Committee in an AAALAC accredited vivarium.

Note that full information on the approval of the study protocol must also be provided in the manuscript.

Clinical data

Policy information about clinical studies

All manuscripts should comply with the ICMJE guidelines for publication of clinical research and a completed CONSORT checklist must be included with all submissions.

Clinical trial registration	Clinicaltrials.gov identifier: NCT04595747
Study protocol	The full trial protocol has been provided and can be found in the Supplementary Information.
Data collection	From May 2021 to August 2023, 24 eligible adult patients were enrolled from 11 centers in the United States.
Outcomes	The primary endpoint was confirmed objective response rate, using radiographic response by RECIST v1.1. Secondary endpoints were progression-free survival and safety and tolerability as assessed per the Common Terminology Criteria for Adverse Events (CTCAE) v5.0. Exploratory objectives were to evaluate serial measurements of FGF3 and FGF4 and FGFR in serial biopsies, to perform whole exome sequencing in serial biopsies, and to explore rogaratinib with pharmacodynamic effects.

Plants

Seed stocks	N/A
Novel plant genotypes	N/A
Authentication	N/A
