# Supplementary figures and images for: Fibroblast growth factor receptor inhibition for succinate dehydrogenase-deficient gastrointestinal stromal tumors: a phase 2 trial
Source: Nat Med. 2026 May 26;32(6):2191–200. doi: 10.1038/s41591-026-04376-9 (PMC13279270; doi:10.1038/s41591-026-04376-9)

**FGFR1**  
(145kDa/120kDa)

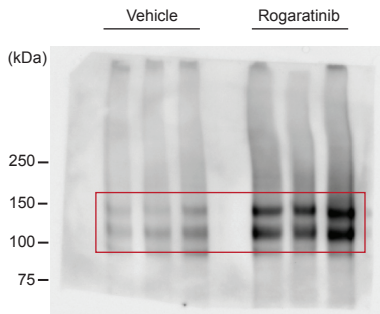

**Actin**  
(42kDa)

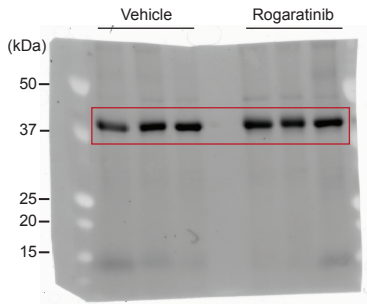

**P-FGFR1**  
(145kDa/120kDa)

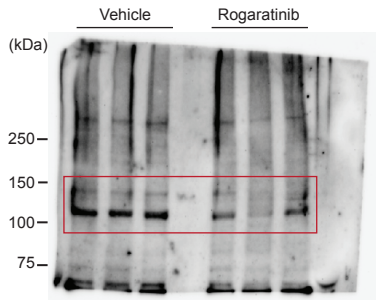

**Actin**  
(42kDa)

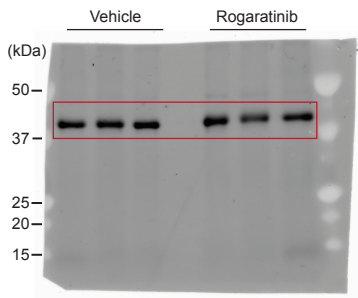

Supplement: Supplementary file 8 — Uncropped gels. [file 41591_2026_4376_MOESM8_ESM.pdf]
